# Synthesis, docking and characterization of some novel 5-(S-alkyl)-1.3.4-thiadiazole-2-carboxamide derivatives as anti-inflammatory and antibacterial agents

**DOI:** 10.1186/s13065-024-01237-9

**Published:** 2024-07-27

**Authors:** Ahmed M. El-Saghier, Asmaa Abdul-Baset, Omer M. El-Hady, Walaa M. Abd El-Raheem, Asmaa M. Kadry

**Affiliations:** 1 Chemistry Department, Faculty of Science, Sohag University, Sohag, 82524 Egypt; 2 Botany and Microbiology Department, Faculty of Science, Sohag University, Sohag, 82524 Egypt

**Keywords:** Carboxamide, Thioxoacetamide, 1,3,4-thiadiazole, Thiohydrazides, Antimicrobial, Anti-inflammatory agent

## Abstract

Because of the great pharmacological and industrial significance of 1,3,4-thiadiazole and its related compounds, researchers are still very interested in them. For this reason, in this study, we looked at ways to create new hybrid compounds containing carboxamide and 1,3,4-thiadiazole moieties. The thioxoacetamide derivatives used to make these compounds were reacted with various alkylated reagents to produce multiple *S*-alkyl groups. Additionally, these compounds were reacted with aldehydes to form novel derivatives known as 5-(substituent)-*N*-phenyl-1,3,4-thiadiazole-2-carboxamide. Here, we used the agar well diffusion method to examine the antibacterial activity of all the produced compounds against a few pathogenic bacteria that were resistant to multiple drugs. Additionally, look into their capacity to lower inflammation through the use of bovine serum albumin in the protein denaturation procedure. The substances were characterized by spectral analysis (IR, ^1^HNMR, ^13^CNMR and Elemental Analysis), and efficient as antibacterial agents against all the tested bacterial strains, except for *Escherichia coli*. Compounds **4a** and **8c** showed the highest level of inhibition zone against Gram-positive bacteria (*Staph. aureus, Bacillus subtilis*) at concentration 0.3, 0.4 and 0.5 mg/ml compared with ciprofloxacin at the same concentrations. The results demonstrated that every compound has significant anti-inflammatory activity. At a concentration of 250 µg/ml, compounds **3a**, **4c** and **8c** had the highest percentage inhibition of protein denaturation when (83.24%, 86.44% and 85.14%, respectively) compared to other compounds and diclofenac sodium as reference drug. Comparing compounds **4c** and **8c** to ciprofloxacin and diclofenac sodium, they showed powerful antibacterial and anti-inflammatory action. Furthermore, an investigation using molecular docking against DHPS from *S. aureus* (PDB ID: 6CLV) showed a strong connection with the intended protein and an elevated docking score, making it a prime candidate for antibiotics.

**Supplementary Information:**

The online version contains supplementary material available at 10.1186/s13065-024-01237-9.

## Background

Microbiological diseases are the most critical issue facing the economy and the world's health [1]. It has recently become more challenging to treat bacterial infections with conventional medicines [2]. Growing concern is being expressed throughout the world over the growth of bacterial resistance to well-known treatments and hospital-acquired illnesses [3]. In actuality, the development of microbial resistance to commercially accessible antibacterial medications is the main cause of illness and mortality [4]. Microbiological disorders that have recently caused a great deal of pain for humans include the epidemic of the plague, diphtheria, cholera, typhoid fever, a respiratory infection, and tuberculosis [5]. Additionally, some recent clinical studies mention the growth in enterococci that are resistant to vancomycin, *Staphylococcus epidermidis*, and methicillin-resistant *Staphylococcus aureus* (MRSA), which are the most prevalent bacterial infections that cause death in the majority wealthy countries [6, 7]. As per the World Health Organisation (WHO), traditional antibiotic therapy typically fails to treat diseases caused by resistant germs, which increases the risk of mortality and lengthens suffering [8]. Therefore, the development of novel antimicrobial drugs that differ from the widely used categories of antibacterial agents is still necessary [9]. Moreover, one potential solution to the problem of overloaded multidrug resistance (MDR) is the development of novel drugs with distinctive mechanisms of action to prevent cross-resistance with currently available therapies [10]. Because of their broad range of biological functions, heterocyclic ring structures in organic compounds continue to garner a lot of research. Numerous synthetic compounds that exhibit appealing biological effects such as antiviral [11], anticancer [12], cytotoxic [13], anticonvulsant [14], antihyperlipedemic [15], anti-inflammatory [16], analgesic [17], antidepressant [18], antioxidant [19], anti-pesticide [20], anti-COVID [21], antileishmanial [22], and antituberculosis [23] properties commonly use the scaffold 1,3,4-thiadiazole.

**Fig. 1:**
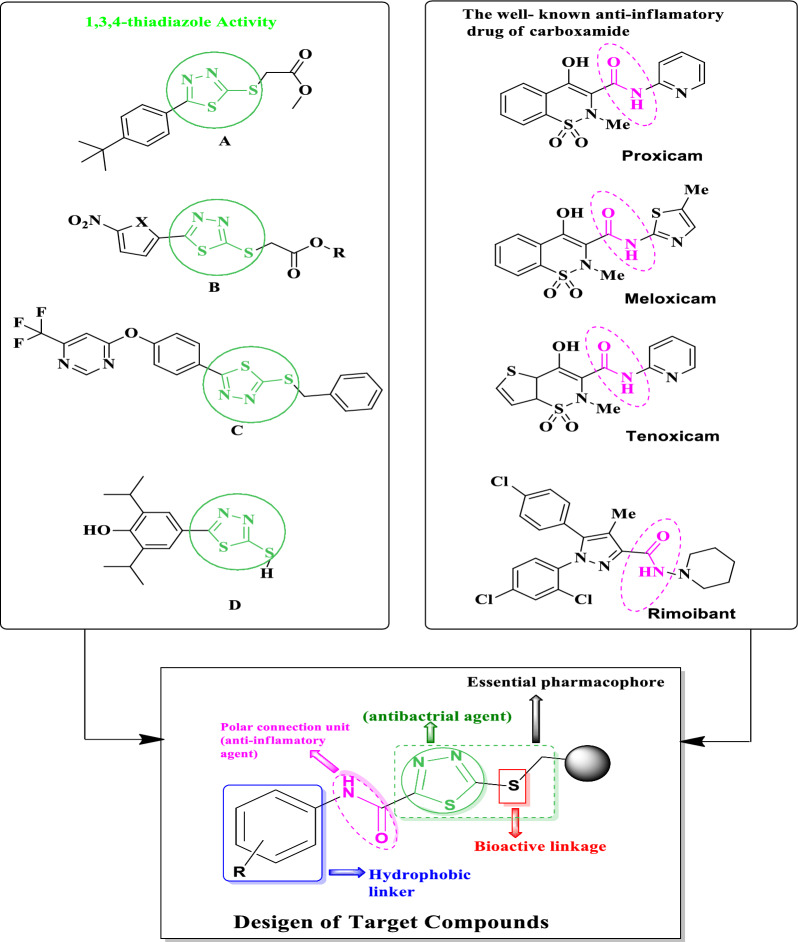
Strategy employed for designing 1.3.4-thiadiazole-2-carboxamide derivatives

Many thiadiazole compounds have found extensive usage in chemotherapeutics as antimicrobial and antibacterial agents [24] that are effective against a wide range of pathogenic bacteria and resistant mycobacterium, such as compounds **A** and **B**. Moreover, mycobacterial activity has been observed to be significantly inhibited by compound **B** (IC_50_ = 0.23 g/ml) [25]. Compound **C** was discovered to be superior to the industry standard (pyrimethanil) when the synthetic 1,3,4-thiadiazole scaffolds were tested using the mycelial growth rate method against a few fungus strains [26]. However, scaffolds **D** have anti-inflammatory activity and demonstrate COX-2 selectivity in the J774A.1 murine macrophage cell line [27]. (Fig. 1). The impressive anti-inflammatory properties of both heterocycles and carboxamide units have been demonstrated. As a result, a lot of research has focused on creating and studying oxicam derivatives as pharmacological agents. The success of the nonsteroidal anti-inflammatory medicines (NSAIDs) piroxicam (Feldene®), meloxicam (Mobic®), and tenoxicam stimulated research in this topic (Fig. 1). Additionally, Rimonabant exerted high activity via the inhibition of COX-2 (inducible) induced at sites of inflammation [28, 29].

As part of our ongoing efforts to produce anti-infective medicines [30–35]. In this study, we design and synthesize several new prototypes containing two pharmacophores, carboxamide and 1,3,4-thiadiazole inside one structural framework using environmentally friendly processes starting with 2-hydrazinyl-*N*-phenyl-2-thioxoacetamide derivatives [36]. We tested their anti-bacterial and anti-inflammatory activity for bioactive compounds.

## Result and discussion

### Chemistry

**Scheme 1:**
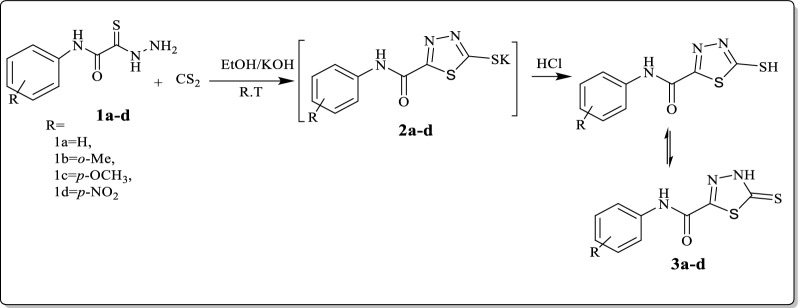
Synthesis of *N*-phenyl-5-thioxo-4,5-dihydro-1,3,4-thiadiazole-2-carboxamide derivatives

As a continuation of our strategy is to determine methods to utilize these molecules as the basis for the synthesis of many different five, six, and seven-membered rings [37–41]. Reaction of thioxoacetamide derivatives **1a–d** with carbon disulfide and potassium hydroxide in ethanol at room temperature considered an efficient method to synthesis potassium 5-(phenylcarbamoyl)-1,3,4-thiadiazole-2-thiolate derivatives (**2a–d)**, which treated with concentrated hydrochloric acid until pH 2–3 to afford novel moiety of 1,3,4-thiadiazole derivatives **3a–d** that can be used as a building block of some new 1,3,4-thiadiazole analogous (Scheme 1). The IR spectrum of compound **3a–d** revealed the disappearance of NH_2_ group. ^1^HNMR for compound **3a** showed new singles at 15.06 for NH_thiadiazole_ group, disappeared by D_2_O, at the same time the peaks for amino group are disappeared. All the compounds show a new peak above 190 ppm in ^13^C NMR which come back to C=S of the formed 1,3,4-thiadiazole rings.

**Scheme 2:**
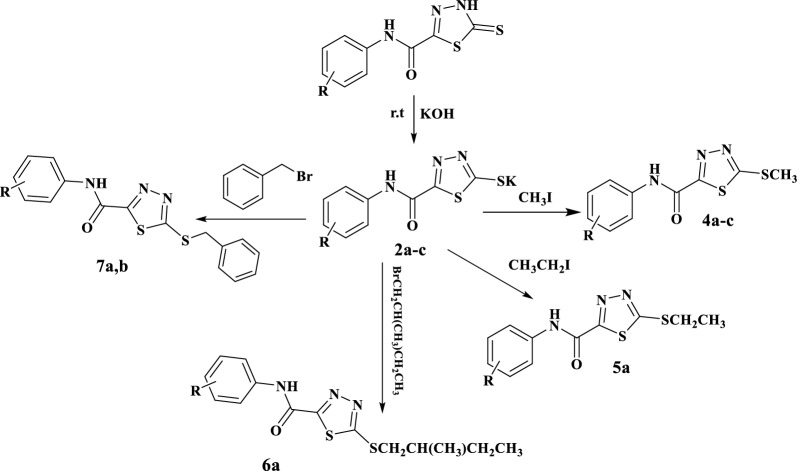
Synthesis of 5-(S-alkyl)-1.3.4-thiadiazole-2-carboxamide derivatives

Moreover, compounds **2a–c** reacted with active halo compounds namely, methyl iodide ethyl iodide, 1-bromo-2-methylbutane and (bromomethyl)benzene at low temperature to give the corresponding S-alkyl derivatives with a substantial output, economical, gentle, straightforward, and environmentally friendly approach that produces suitable behaviors, see Scheme 2. Structures of the recently obtained compounds were verified based upon their IR, ^1^H-NMR, ^13^C-NMR, and elemental analyses. The IR spectra of compounds **4–7** exhibited the presence of broad band: at 3234–3537 cm^−1^ corresponding to NH groups, at 1660–1680 cm^−1^ corresponding to alkyl groups. The ^1^H-NMR spectrum, for example, of compound **4a–c** revealed the presence of a broad band at 10.60–11.03 ppm characterized to NH group, a singlet signal at 2.24–2.84 ppm corresponding to S-alkyl group. ^13^CMR spectrum of compound **4a** revealed the following signals: 165.29, 156.32 (2C, thiadiazole), 173.51 (C=O), four signals at 138.12, 129.18, 125.11, 121.27 ppm for 5C of Aromatic group and singlet signal at 17.37 ppm of methylthiol group.

**Scheme 3:**
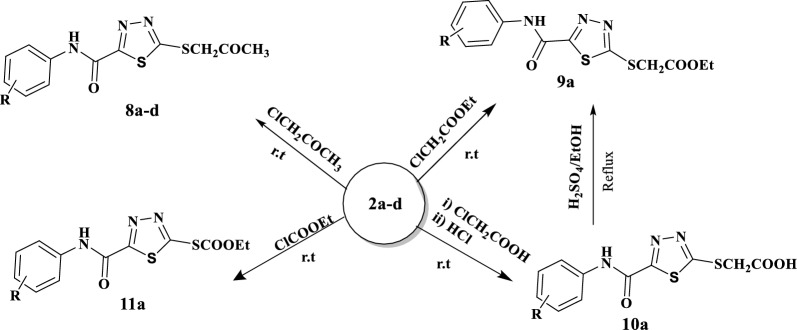
Synthesis of 5-(S-alkyl)-1.3.4-thiadiazole-2-carboxamide derivatives

Nevertheless, alkylation reaction of compounds **2a–d** with chloroacetone, ethyl chloroacetate, chloroacetic acid and ethyl chloroformate, afforded the corresponding 5-(S-alkyl) sulfanyl-1,3,4-thiadiazole-2-carboxamide derivatives **8a–c**, **9a**, **10a** and **11a**, respectively. Additionally, it was easily to synthesis compound **9a** by another way through conversion of acidic group in compound **10a** into ester group in compound **9a**, see Scheme 3. The structures of the obtained 5-(S-alkyl)-1.3.4-thiadiazole-2-carboxamide derivatives **8–11** were distinguished by their spectral and elemental data. For instance, the IR spectrum of compound 9a had peak absorption for the NH group at 3537 cm^−1^ and another distinctive band for the novel C=O group at 1737 cm^−1^. Where the ^1^H-NMR spectrum of this compound showed a singlet signal at 11.05 ppm for NH group which disappeared by D_2_O, a multiples signals between 7.15 and 7.83 ppm for aromatic protons, singlet signal at 4.35 ppm for S–CH_2_– group, quartet signal at 4.20–4.14 ppm and a triplet signal at 1.23–1.20 ppm with coupling constant equals to 7.08 Hz, which could be assigned for CH_2_CH_3_ groups. The signals of ^13^CNMR confirmed the expected structure by appearance of new carbonyl group at 168.08 ppm. Finally, the DEPT-135 obviously distinguished between the –CH_2_– (62.12 ppm) and –CH_3_ (14.56 ppm) of the ethyl chain where, it showed one CH_3_ with a positive phase and two CH_2_ with a negative one.

**Scheme 4:**
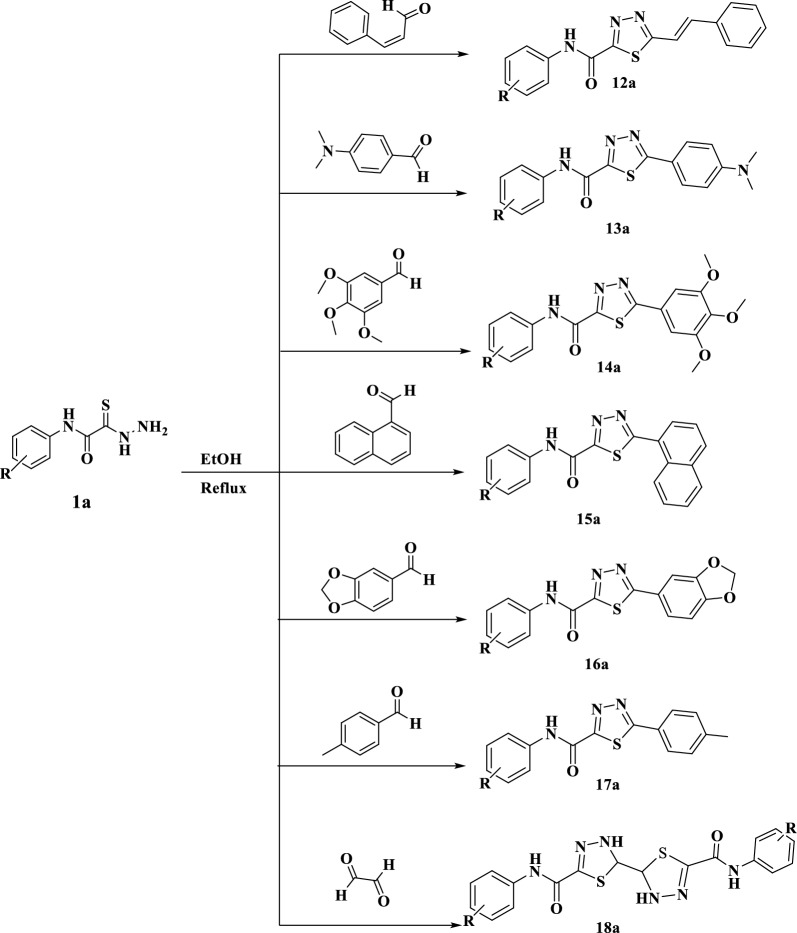
Synthesis of 5-(substituent)-*N*-phenyl-1,3,4-thiadiazole-2-carboxamide derivatives

Reaction of compound **1a** with different aldehydes namely, cinnamaldehyde, *p*-*N, N* dimethylaminobenzaldehyde, 3,4,5-trimethoxy-benzaldehyde, 1-naphthaldehyde, pipreonal, *p* methylbenzaldehyde and glyoxal to afford; 5-(substituent)-*N*-phenyl-1,3,4-thiadiazole-2-carboxamide derivatives **(12a–18a)**, respectively, Scheme 4. IR spectrum of compounds **12a–18a** showed the disappearance of NHNH_2_ group absorption bands. The ^1^H-NMR spectrum for compound **12a** showed signal at 10.70 ppm for NH group (disappeared by D_2_O), between 7.85–7.08 ppm for aromatic protons and 6.68 ppm for the –CH=CH– group. The signals of ^13^C NMR spectrum confirmed the expected structure by appearance of new group signals at 163.20, 158.13 (2-S–C=N) and 120.74, 112.13 ppm for CH=CH– group. The ^1^H-NMR spectrum for compound **13a** showed signal at 11.31 ppm for NH group (disappeared by D_2_O), between 8.23–7.09 ppm for aromatic protons and a new singlet signal at 2.84 ppm for the two methyl groups. The signals of ^13^CNMR spectrum confirmed the expected structure by appearance of new group signals at 171.33 (C=O), 166.60, 162.53 (2C, thiadiazole) and 36.42 ppm (2CH_3_). In the case of compound **18a,** its ^1^H-NMR spectrum showed two signals at 10.19 and 9.04 ppm for NH groups (disappeared by D_2_O), between 7.74–7.06 ppm for aromatic protons and new singlet signal at 5.53 for the two -CH groups. The signals of ^13^CNMR spectrum confirmed the expected structure by appearance of new signal at 159.13 (C=O), 139.04 (C, Thiadiazole), 138.66, 129.05, 124.29, 120.74 for aromatic ring and 76.13 ppm for quaternary carbon atom. Also, its Dept -135 spectrum showed signals at 129.09, 124.31, 120.74 ppm for aromatic ring and 75.98 ppm (CH_thiadiazole_) in the positive phase.

### Biological evaluation

#### Antimicrobial screening

**Fig. 2:**
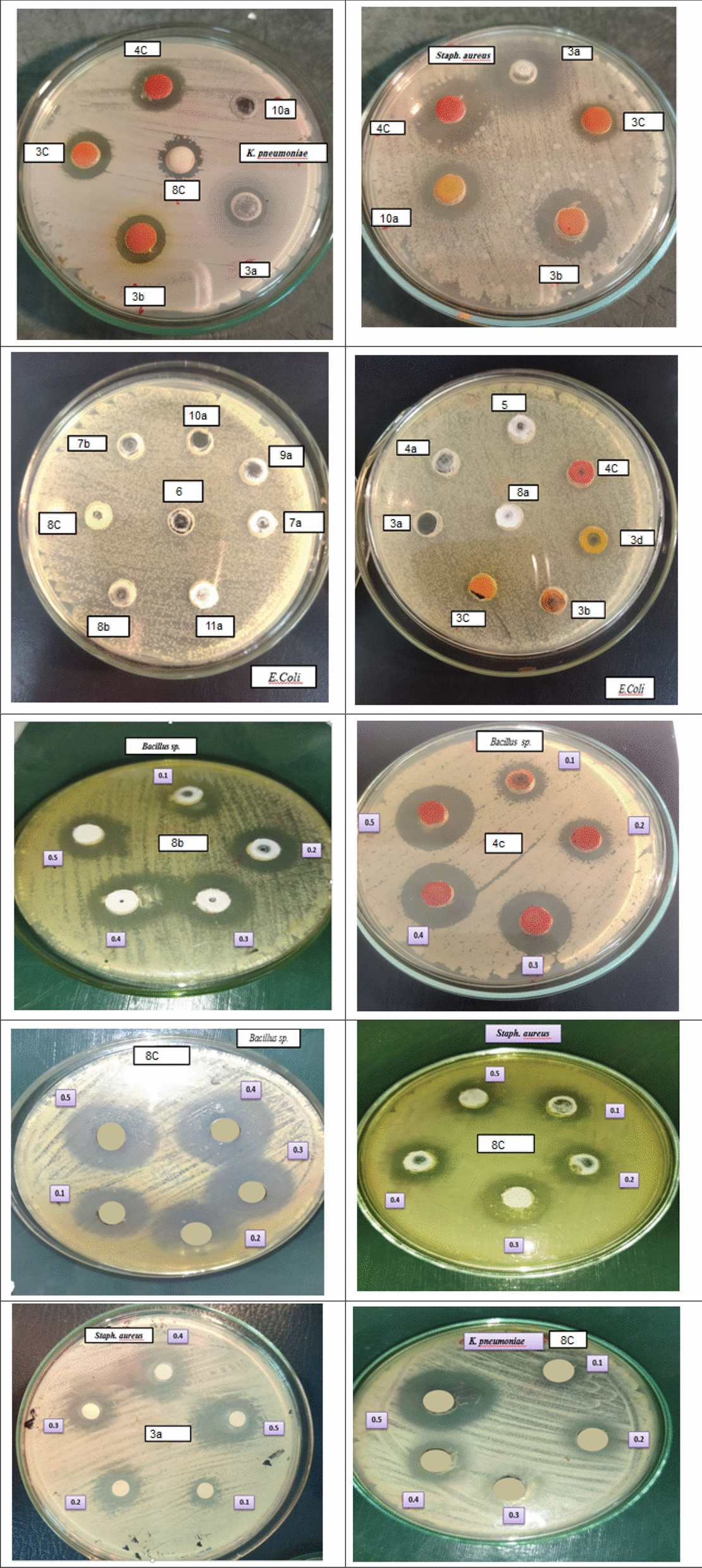

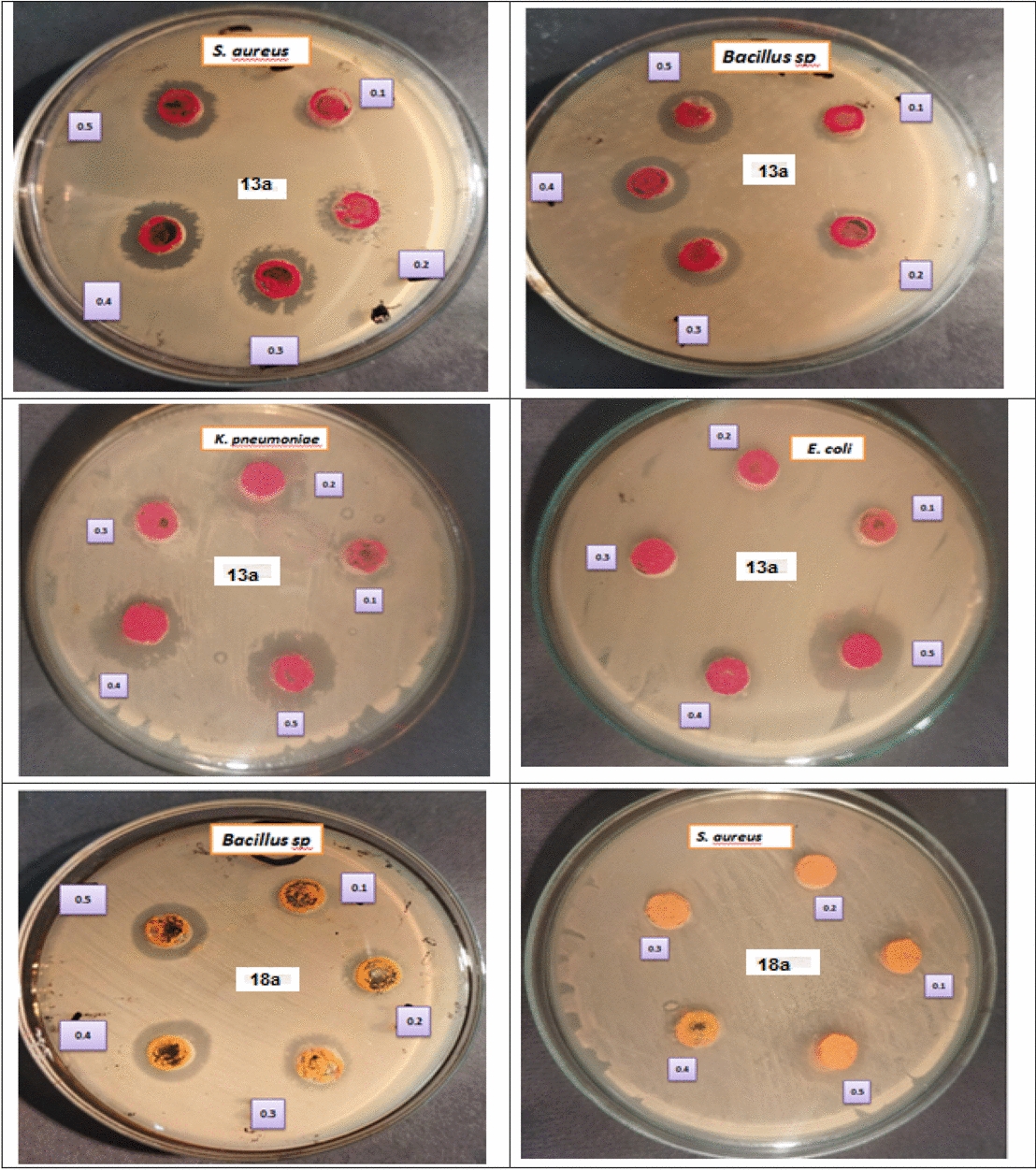
Antibacterial activity of the tested compounds

Antimicrobial activity of the tested compounds was investigated against multidrug pathogenic bacteria. The tested compounds showed potential antibacterial effect against *Staph. aureus, Bacillus subtilis* and* K. pneumonia* and no inhibitory effect against* E. coli*. Ciprofloxacin is used in this investigation as a control. In clinic and hospital settings, ciprofloxacin is a widely used broad-spectrum antibiotic. The closest compounds to ciprofloxacin were **4c** and **8c**, which were more effective against Gram-positive bacteria (*Staph. aureus, Bacillus subtilis*) at concentration 0.3, 0.4 and 0.5 mg/ml. Furthermore, compounds **3a, 4a** and **6a** showed potential antibacterial effect against *Staph. Aureus* and* Bacillus subtilis*, respectively, as shown in (Table S1(supplementary file), Fig. 2).

#### Statistical results of antimicrobial screening

**Fig. 3:**
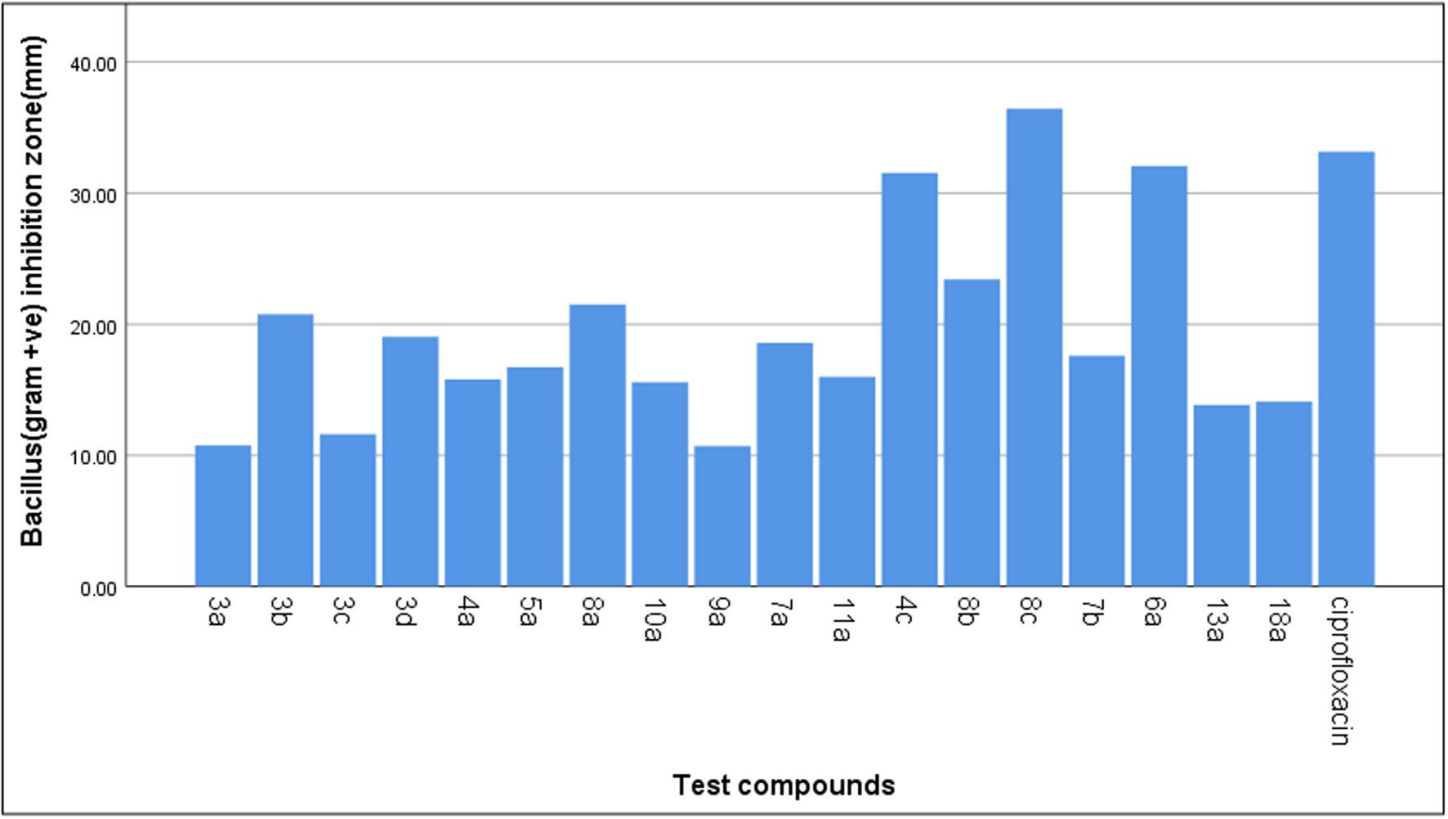
Shows a comparison between test compounds and ciprofloxacin with the size of the inhibition zone of *Bacillus* (gram +ve) strain of bacteria

Nineteen compounds studied with different concentrations on both Gram-positive and Gram-negative bacteria, formed four subsets in accordance with the zone of inhibition values. A one-way ANOVA was conducted to compare the effect of in-vitro antibacterial activity of compounds (Table 1). From Table 1, we have found a statistically significant result. It is observed that the in-vitro antibacterial activity of compounds **19** (Ciprofloxacin), **8c, 6a**, and **4c** significantly different from all other compounds. But Ciprofloxacin is used as standard. It is evident from the ANOVA that the compounds (**8c, 6a**, and **4c**), exhibited significantly high antibacterial activity compare to the all other synthesized tested compounds and also with standard. As shown in Table 1, compound **8c** exhibit significantly high antibacterial activity against *S. aureus* (33.26 ± 4.73) and significantly excellent antibacterial effect against *Bacillus* strain (36.44 ± 4.05) (Fig. 3). Moreover, compound **6a, 4c** had exhibit significantly high antibacterial effect against *Bacillus* strain as mean = 32.66, 31.54 respectively.

**Table 1:** In-vitro antibacterial activity of tested compounds

Bacteria	Test compound	Mean of the size of the inhibition zone (mm)	Std. deviation Deviation	*p-*value*
*S. aureus*	3a	31.55	5.45	0.0001
	3b	29.91	3.15	
	3c	25.17	4.63	
	3d	22.16	4.36	
	4a	30.68	3.02	
	5a	18.87	5.89	
	8a	18.67	4.05	
	10a	14.21	2.75	
	9a	12.65	7.05	
	7a	24.08	3.85	
	11a	21.47	5.92	
	4c	30.41	4.80	
	8b	27.08	5.31	
	8c	33.27	4.73	
	7b	19.79	7.73	
	6a	19.98	5.95	
	13a	4.30	5.54	
	18a	14.34	3.47	
	Ciprofloxacin	43.97	4.28	
*Bacillus *sp.	3a	10.77	7.19	0.0001
	3b	20.76	5.80	
	3c	11.62	6.83	
	3d	19.04	6.80	
	4a	15.80	4.83	
	5a	16.73	5.16	
	8a	21.51	5.95	
	10a	15.59	8.35	
	9a	10.71	5.60	
	7a	18.59	2.37	
	11a	15.99	4.19	
	4c	31.54	4.76	
	8b	23.43	2.61	
	8c	36.43	4.04	
	7b	17.59	4.36	
	6a	32.07	5.04	
	13a	13.85	4.32	
	18a	14.11	2.58	
	Ciprofloxacin	33.15	7.18	
*K. pnemoniae*	3a	15.17	2.43	0.0001
	3b	17.47	3.37	
	3c	14.84	2.29	
	3d	19.89	5.96	
	4a	19.26	6.11	
	5a	20.32	6.38]	
	8a	15.44	8.25	
	10a	0.00	0.00	
	9a	6.53	5.61	
	7a	20.23	2.55	
	11a	11.42	6.05	
	4c	19.24	3.10	
	8b	13.55	8.43	
	8c	26.32	7.10	
	7b	15.40	4.35	
	6a	0.00	0.00	
	13a	0.00	0.00	
	18a	15.24	10.91	
	Ciprofloxacin	38.16	9.46	
*E. coli*	3a	0.00	0.00	0.0001
	3b	0.00	0.00	
	3c	0.60	2.32	
	3d	0.00	0.00	
	4a	0.00	0.00	
	5a	0.00	0.00	
	8a	0.00	0.00	
	10a	0.00	0.00	
	9a	0.00	0.00	
	7a	0.00	0.00	
	11a	0.00	0.00	
	4c	4.25	5.49	
	8b	0.00	0.00	
	8c	6.02	5.27	
	7b	0.00	0.00	
	6a	0.00	0.00	
	13a	0.00	0.00	
	18a	0.00	0.00	
	Ciprofloxacin	45.02	11.25	

* *p* < 0.05 (significant), *p* < 0.01 (highly significant), *p* < 0.001 (very highly significant), NS: Non significant *p* > 0.05

#### Anti-inflammatory activity of the tested compounds

Proteins eliminate their tertiary and secondary structures when exposed to an external stressor or substance, such as a powerful base or acid, a highly concentrated inorganic salt, an organic solvent, or heating. This process is referred to as denaturation. The expected process of denaturation is a modification in electrostatic, hydrogen, hydrophobic, and disulphide coupling. There is a dose-dependent capacity of certain anti-inflammatory medications to avoid denaturation of proteins brought about by heating [42]. In this study all compounds were shown to have strong anti-inflammatory action by employing a protein denaturation inhibition technique at concentration of 50, 100, 150, 200 and 250 µg/ml in a concentration-dependent manner (Table 2). In comparison to other compounds, the compounds **3a**, **4c** and **8c** showed the highest levels of inhibition at concentrations of 250 µg/ml with percentage inhibition 83.24%, 86.44% and 85.14%, respectively. At the same concentrations, compounds **3b** and **8b** exhibited significant anti-inflammatory activity with percentage inhibition 81.99% and 80.99%. These substances could therefore be a viable substitute for agents that have anti-inflammatory properties. Hence, it could be a valuable medicinal ingredient for the treatment of bacterial infections and inflammation.

**Table 2:** Anti-denaturation activity of the tested compounds and positive control

Concentrations µg/ml	Test compound	Mean of inhibition %	Std. deviation	*p*-value*
50	3a	63.19	0.00	0.0001
	3b	59.25	0.00	
	3c	58.44	0.00	
	3d	58.63	0.00	
	4a	30.60	0.00	
	5a	2.76	0.00	
	8a	1.39	0.00	
	10a	2.12	0.00	
	9a	4.76	0.00	
	7a	35.22	0.00	
	11a	34.72	0.00	
	4c	64.13	0.00	
	8b	59.75	0.00	
	8c	65.23	0.00	
	7b	32.72	0.00	
	6a	30.60	0.0	
	13a	35.22	0.0	
	18a	6.76	0.0	
	Diclofenac sodium	70.85	0.0	
100	3a	73.46	0.0	0.0001
	3b	68.42	0.0	
	3c	67.33	0.0	
	3d	68.45	0.0	
	4a	35.15	0.0	
	5a	9.32	0.0	
	8a	6.37	0.0	
	10a	8.54	0.0	
	9a	9.78	0.0	
	7a	40.78	0.0	
	11a	41.15	0.0	
	4c	74.40	0.0	
	8b	68.84	0.0	
	8c	74.46	0.0	
	7b	40.66	0.0	
	6a	39.15	0.0	
	13a	40.78	0.0	
	18a	10.72	0.0	
	Diclofenac sodium	79.55	0.0	
150	3a	78.44	0.0	0.0001
	3b	75.14	0.0	
	3c	72.50	0.0	
	3d	73.80	0.0	
	4a	41.31	0.0	
	5a	16.87	0.0	
	8a	11.42	0.0	
	10a	14.37	0.0	
	9a	18.47	0.0	
	7a	44.32	0.0	
	11a	46.31	0.0	
	4c	77.64	0.0	
	8b	74.14	0.0	
	8c	78.64	0.0	
	7b	45.52	0.0	
	6a	43.31	0.0	
	13a	48.30	0.0	
	18a	17.47	0.0	
	Diclofenac sodium	82.64	0.0	
200	3a	81.72		0.0001
	3b	78.00	0.0	
	3c	74.66	0.0	
	3d	74.43	0.0	
	4a	46.65	0.0	
	5a	22.93	0.0	
	8a	16.62	0.0	
	10a	20.93	0.0	
	9a	25.33	0.0	
	7a	58.00	0.0	
	11a	52.65	0.0	
	4c	82.02	0.0	
	8b	78.56	0.0	
	8c	82.72	0.0	
	7b	52.43	0.0	
	6a	49.65	0.0	
	13a	62.21	0.0	
	18a	25.63	0.0	
	Diclofenac sodium	85.46	0.0	
250	3a	83.24	0.0	0.0001
	3b	81.99	0.0	
	3c	79.65	0.0	
	3d	78.00	0.0	
	4a	51.43	0.0	
	5a	29.12	0.0	
	8a	21.86	0.0	
	10a	23.12	0.0	
	9a	29.76	0.0	
	7a	64.62	0.0	
	11a	60.43	0.0	
	4c	85.14	0.0	
	8b	80.99	0.0	
	8c	86.44	0.0	
	7b	60.77	0.0	
	6a	55.43	0.0	
	13a	70.62	0.0	
	18a	33.76	0.0	
	Diclofenac sodium	97.85	0.0	

* *p* < 0.05 (significant), *p* < 0.01 (highly significant), *p* < 0.001 (very highly significant), NS: Non significant *p* > 0.05

#### Statistical results of anti-inflammatory activity

**Fig. 4:**
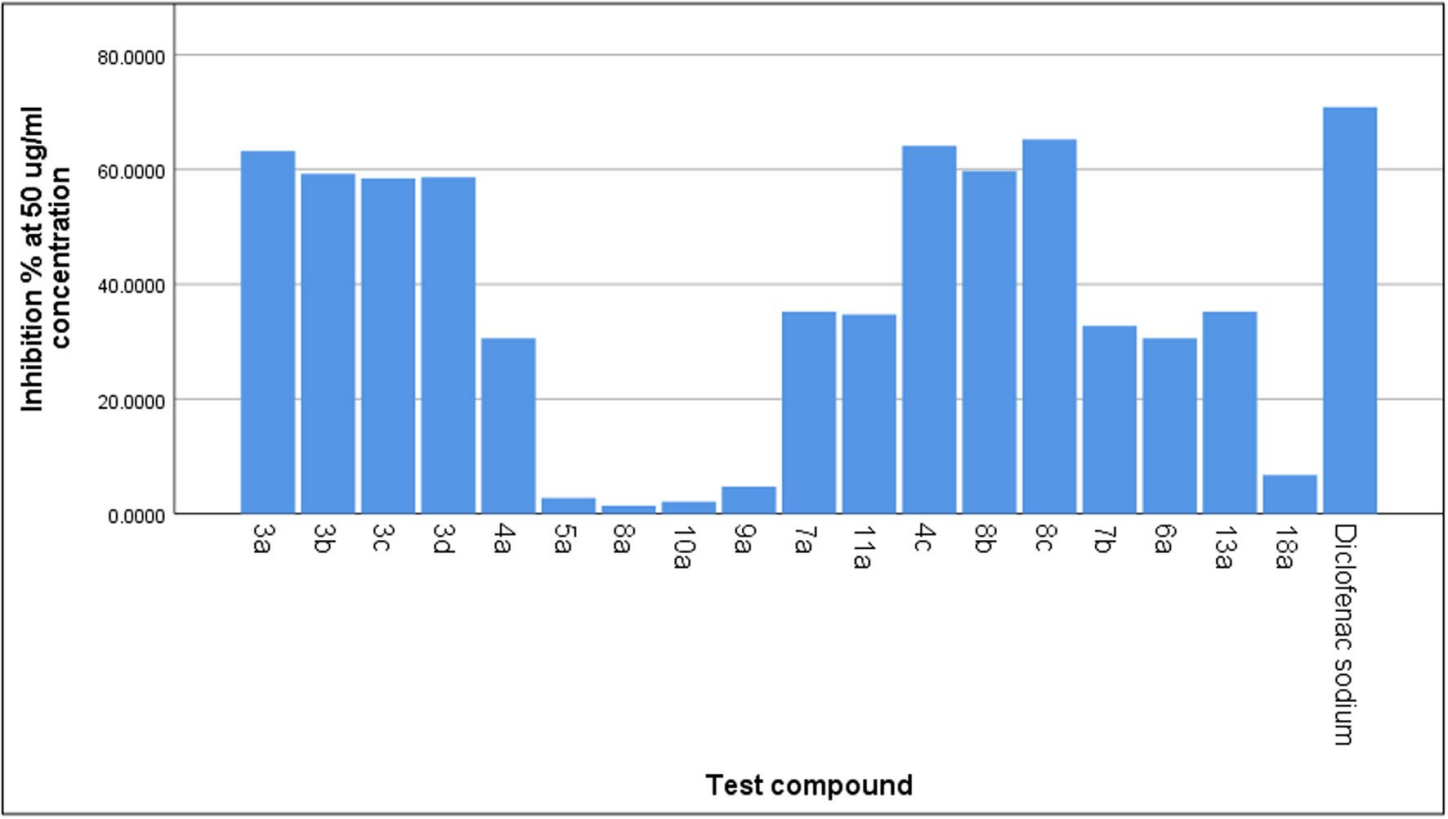
Shows a comparison between test compounds with 50 µg/ml concentration with % of inhibition

All synthesized compounds were screened for in-vitro anti-inflammatory activity by inhibition of protein denaturation method using diclofenac as a standard drug. From Table 2, we have found a statistically significant result in all concentration (50, 100, 150, 250 µg /ml) in comparison to different test compounds. It is evident from the ANOVA that the compounds **3a**, **4c, 8c, 3b** and **8b**, exhibited significantly high anti-inflammatory compare to the all-other synthesized tested compounds. Compound **8c** showed significant effect mean 65.23 compering with 70.85 for Diclofenac sodium.at concentrations of 50 µg/ml as shown in Fig. 4.

## Conclusion

Synthesis, characterization, and investigation of some 1,3,4-thiadiazole derivatives which prepared from thioxoacetamide derivatives were studied, their reactions with some alkyl halides to make alkylation reaction and with some aldehydes to form novel 5-(substituent)-*N*-phenyl-1,3,4-thiadiazole-2-carboxamide derivatives were investigated. Finally, we studied the possibility of 1,3,4-dihydrothiadiazole derivatives as antimicrobial potential on some multidrug-resistant pathogenic bacteria. Gram-positive and Gram-negative bacteria are both targets of antimicrobial action using the agar well diffusion method then screening data is subjected to statistical analysis using one way ANOVA technique. The compounds exhibited antibacterial efficacy against all tested bacterial strains except *Escherichia coli*. Also, the result revealed that all compounds possessed potent significant anti-inflammatory activity. In deep study, compounds **4c** and **8c** possess significant antimicrobial and anti-inflammatory activity as compared to ciprofloxacin and diclofenac sodium. Additionally, a study employing molecular docking against DHPS from *S. aureus* (PDB ID: 6CLV) found that it is a great option for antibiotics since it is used by nearly all bacterial strains to synthesize nucleic acids. The molecular docking study exhibited positive interaction with the target protein and a high docking score especially for compounds **3a**, **4c**, **8d** and **18a**. According to the study's findings, the substances in question have strong antibacterial and anti-inflammatory properties. The overall results of this study can be considered as very promising in the perspective of new antimicrobial drugs, especially when the medical importance of tested microorganisms is considered. However, pharmacological and toxicological studies, will be necessary to confirm this hypothesis.

## Methods/Experimental

### Chemistry

Thin layer chromatography (TLC) was employed to track all reactions utilizing percolated dishes of silica gel G/UV-254 with a 0.25 mm thickness (Merck 60F254) and UV light (254 nm/365 nm) enable visualization. The uncorrected Kofeler melting point instrument was used to record all melting points. On an FT-IR spectrophotometer, KBr pellets were used to analyses IR spectra. At Sohag University, spectral characterizations of the compounds, Bruker Avance III at 400 MHz and 100 MHz for ^1^H and ^13^CNMR (DMSO-d_6_, δ ppm), respectively were used. Tetramethylsilane (TMS) was selected as the standard for internal measurement and its chemical shifts (δ) were expressed in parts per million (ppm). TMS (= 0 ppm) or DMSO (= 39.51 ppm) were employed as internal standards for ^13^C NMR. A Perkin-Elmer CHN analyzer model provided elemental analyses as shown in supplementary file.

#### General synthesis of N-phenyl-5-thioxo-4,5-dihydro-1,3,4-thiadiazole-2-carboxamide derivatives (3a–d)

2-Hydrazino-*N*-Phenyl-2-thioxoacetamide **(1a–d)** (0.01 mmol), potassium hydroxide (0.03 mmol), carbon disulfide (0.03 mmol) was stirring in ethanol at room temperature for 6 h., then was poured in 20 ml distilled water. Concentrated hydrochloric acid was added until pH 2–3, precipitated formed crystallized with ethanol, see Figure (S1–S10).

*N*-phenyl-5-thioxo-4,5-dihydro-1,3,4-thiadiazole-2-carboxamide (**3a**): White crystals, yield 93%, mp. 180–182 °C; FT-IR (ATR) *δ*_max_: 3345, 3191 (2NH str.), 3104 (CH_arom_ str.), 1678 (C=O str.), 1659 (C=N str.), 1236 (C=S str.); ^1^H NMR: *δ* 15.06 (s, H, NH_thiadiazole_, exchangeable by D_2_O), 10.79 (s, H, NH, exchangeable by D_2_O), 7.77–7.14 (m, 5H, ArH) ppm; ^13^C NMR: *δ* 190.78 (C=S), 157.25 (C=O), 155.34, 137.91 (2C, Thiadiazole), 129.20, 125.23, 121.34 ppm (C of Arom.). Anal. Calcd. for C_9_H_7_N_3_OS_2_ (237.30): C, 45.55; H, 2.91; N, 17.71; S, 27.02% Found: C, 45.65; H, 2.81; N, 17.61; S, 27.12%.

5-Thioxo-*N*-(o-tolyl)-4,5-dihydro-1,3,4-thiadiazole-2-carboxamide (**3b**): Orange crystals, yield 91%, mp. 177–179 °C; FT-IR (ATR) *δ*_max_: 3342, 3311 (2NH str.), 3057 (CH_arom_ str.), 1681 (C=O str.), 1648 (C=N str.), 1222 (C=S str.); ^1^H NMR: *δ* 15.06 (s, H, NH_thiadiazole_), 10.79 (s, H, NH), 7.39–7.19 (m, 4H, ArH), 2.24 ppm (s, 3H, CH_3_); ^13^C NMR: *δ* 190.55 (C=S), 157.25 (C=O), 155.15, 137.91 (2C, Thiadiazole), 134.62, 132.10, 129.20, 125.23, 121.34 (Arom.), 18.54 ppm (CH_3_). Anal. Calcd. for C_10_H_9_N_3_OS_2_ (251.33): C, 47.79; H, 3.61; N, 16.72; S, 25.52% Found: C, 47.35; H, 3.95; N, 16.52; S, 25.31%.

*N*-(4-methoxyphenyl)-5-thioxo-4,5-dihydro-1,3,4-thiadiazole-2-carboxamide (**3c**): Orange crystals, yield 88%, mp. 193–195 °C; FT-IR (ATR) *δ*_max_: 3348, 3316 (2NH str.), 3132 (CH_arom_ str.), 1675 (C=O str.), 1659 (C=N str.), 1236 (C=S str.); ^1^H NMR: *δ* 15.06 (s, H, NH_thiadiazole_), 10.79 (s, H, NH), 7.77–7.35 (dd, 4H, ArH, *J* = 8.08Hz), 4.18 (s, 3H, OCH_3_) ppm; ^13^C NMR: *δ* 201.67 (C=S), 170.85 (C=O), 165.93 (C, Thiadiazole) 137.94, 129.25, 125.29, 121.31 (Arom.), 54.21 **(**OCH_3_**)** ppm.Anal. Calcd. for C_10_H_9_N_3_O_2_S_2_ (267.33): C, 44.93; H, 3.39; N, 15.72; S, 23.99% Found: C, 44.57; H, 3.75; N, 16.30; S, 23.58%.

*N*-(4-nitrophenyl)-5-thioxo-4,5-dihydro-1,3,4-thiadiazole-2-carboxamide (**3d**): Orange crystals, yield 70%, mp. 201–203 °C; FT-IR (ATR) *δ*_max_: 3351, 3327 (2NH str.), 3136 (CH_arom_ str.), 1678 (C=O str.), 1666 (C=N str.), 1350, 1555 (NO_2_ str.), 1225 (C=S str.); ^1^H NMR: *δ* 15.01 (s, H, NH_thiadiazole_), 10.79 (s, H, NH), 7.77–7.35 ppm (dd, 4H, ArH, *J* = 8.08Hz) ppm; ^13^C NM: *δ* 190.78 (C=S), 157.25 (C=O), 155.15 (C, Thiadiazole) 144.07, 129.25, 129.20, 125.23, 121.34 ppm (Arom.) Anal. Calcd. for C_9_H_6_N_4_O_3_S_2_ (282.30): C, 38.29; H, 2.14; N, 19.85; S, 22.72% Found: C, 38.17; H, 2.24; N, 19.15; S, 22.52%.

#### General synthesis of 5-(S-alkyl)-1.3.4-thiadiazole-2-carboxamide derivatives

A mixture of *N*-phenyl-5-thioxo-4,5-dihydro-1,3,4-thiadiazole-2-carboxamide derivatives **(2a–d)** (0.01 mmol), potassium hydroxide (0.03 mmol) and alkyl halide (0.015 mmol) were added and stirred in ethanol for 2 h. The formed precipitate was collected and crystallized from ethanol.

5-(Methylthio)-*N*-phenyl-1,3,4-thiadiazole-2-carboxamide (**4a**): White crystals, yield 97%, mp. 160–162 °C; FT-IR (ATR) *δ*_max_: 3381 (NH str.), 3156 (CH_arom_ str.), 2923 (CH_3 alip_ str.), 1673 (C=O str.), 1659 (C=N str.); ^1^H NMR: *δ* 11.03 (s, H, NH), 7.85–7.14 (m, 5H, ArH), 2.83 ppm (s, 3H, SCH_3_); ^13^C NMR: *δ* 173.51 (C=O), 165.29, 156.32 (2C, Thiadiazole), 138.12, 129.18, 125.11, 121.27 (Arom.), 17.37 ppm (SCH_3_). Anal. Calcd. for C_10_H_9_N_3_OS_2_ (251.33): C, 47.79; H, 3.61; N, 16.72; S, 25.52% Found: C, 47.99; H, 3.41; N, 16.42; S, 25.80%.

5-(Methylthio)-*N*-(o-tolyl)-1,3,4-thiadiazole-2-carboxamide (**4b**): White crystals, yield 87%, mp. 155–157 °C; FT-IR (ATR) *δ*_max_: 3383 (NH str.), 3107 (CH_arom_ str.), 2988, 2963 (CH_3_ str.), 1679 (C=O str.), 1609 (C=N str.); ^1^H NMR: *δ* 10.60 (s, H, NH), 7.39–7.19 (m, 4H, ArH), 2.30 (s, 3H, CH_3_ Arom), 2.24 (s, 3H, SCH_3_) ppm; ^13^C NMR: *δ* 170.71 (C=O), 165.69, 156.44 (2C, Thiadiazole), 135.25, 134.08, 130.93, 127.11, 126.64, 125.23 (Arom.), 28.93 (SCH_3_), 25.28**)** ppm (CH_3_ Arom.). Anal. Calcd. for C_11_H_11_N_3_OS_2_ (265.35): C, 49.79; H, 4.18; N, 15.84; S, 24.17% Found: C, 49.63; H, 4.22; N, 15.76; S, 24.16%.

*N*-(4-methoxyphenyl)-5-(methylthio)-1,3,4-thiadiazole-2-carboxamide (**4c**): Orange crystals, yield 66%, mp. 198–200 °C; FT-IR (ATR) *δ*_max_: 3379 (NH str.), 3251 (CH_arom_ str.), 2996, 2985 (CH_3alip_ str.), 1675 (C=O str.), 1605 (C=N str.); ^1^H NMR: *δ* 11.07 (s, H, NH), 7.83–7.38 (dd, 4H, ArH, *J*=8.08Hz), 4.04 (s, 3H, OCH_3_), 2.31 ppm (s, 3H, SCH_3_); ^13^C NMR: *δ* 170.85, 165.93, 155.15, 137.94, 129.25, 125.29, 121.31 Arom, 54.21 **(**OCH_3_**)**, 24.64 ppm **(**SCH_3_**)**. Anal. Calcd. for C_11_H_11_N_3_O_2_S_2_ (281.35): C, 46.96; H, 3.94; N, 14.93; S, 22.79% Found: C, 46.77; H, 3.89; N, 15.03; S, 22.64%**.**

5-(Ethylthio)-*N*-phenyl-1,3,4-thiadiazole-2-carboxamide (**5a**): White crystals, yield 73%, mp. 157–159 °C; FT-IR (ATR) *δ*_max_: 3336.85 (NH str.), 3061 (CH_arom_ str.), 2979–2870 (CH_2_CH_3alip_ str.), 1670 (C=O_amidic_ str.),1599 (C=N str.); ^1^H NMR: *δ* 11.04 (s, H, NH), 7.84–7.15 (m, 5H, ArH), 3.41, 3.40, 3.38, 3.36 (q, 2H, SCH_2_, *J* = 6.6 Hz), 1.45, 1.43, 1.41 ppm (t, 3H, CH_3_, *J* = 6.6 Hz); ^13^C NM: *δ* 171.47(C=O), 165.37, 156.31 (2C, Thiadiazole), 138.06, 131.87, 125.18, 121.28 (Arom.), 29.18 (CH_2_), 14.64 ppm (CH_3_). Dept -135 NMR; 129.35,124.90,121.31 Arom, 29.36, 14.57 ppm. Anal. Calcd. for C_11_H_11_N_3_OS_2_ (265.03): C, 49.79; H, 4.18; N, 15.84; S, 24.17% Found: C, 49.39; H, 4.18; N, 15.98; S, 24.03%.

5-((2-Methylbutyl) thio)-*N*-phenyl-1,3,4-thiadiazole-2-carboxamide (**6a**): White crystals, yield 84%, mp. 150–152 °C; FT-IR (ATR) *δ*_max_: 3340 (NH_amidic_ str.), 3061 (CH_arom_ str.), 2961–2869 (CH_aliphatic_ str.), 1667 (C=O_amidic_ str.), 1599 (C=N str.); ^1^H NMR: *δ* 11.04 (s, H, NH), 7.82–7.10 (m, 5H, ArH), 3.33, 3.31 (d, 2H,SCH_2_), 1.62, 1.60, 1.59, 1.53, 1.50 (m, 3H, (CH(CH_3_)CH_2_)), 0.87, 0.86, 0.82 ppm (t, 6H, CH_3_)CH_2_CH_3_); ^13^C NMR: *δ* 171.90 (C=O), 165.44, 156.25 (2C, Thiadiazole), 138.06, 129.15, 125.11, 121.21 (Arom.), 44.50, 37.88, 32.79, 27.40, 22.40 ppm. Anal. Calcd. for C_14_H_17_N_3_OS_2_ (307.43): C, 54.69; H, 5.57; N, 13.67; S, 20.86% Found: C, 54.46; H, 5.77; N, 13.58; S, 20.73%.

5-(Benzylthio)-*N*-phenyl-1,3,4-thiadiazole-2-carboxamide (**7a**): White crystals, yield 95%, mp. 237–239 °C; FT-IR (ATR) *δ*_max_: 3223 (NH str.), 3056 (CH_arom_ str.), 2958 (CH_2alip_ str.), 1667 (C=O_amidic_ str.), 1625 (C=N str.); ^1^H NMR *δ* 11.05 (s, H, NH), 7.83–7.15(m, 10H, ArH), 4.67(s, 2H, SCH_2_) ppm; ^13^C NMR *δ* 171.19 (C=O), 165.87,156.26 (2C, Thiadiazole), 138.03, 136.40, 129.66, 129.25, 129.11, 128.32, 125.23, 121.25 (Arom.), 38.109 ppm (CH_2_). Dept-135 NMR; 129.61, 129.25, 129.11, 128.32, 125.28, 121.31 (Arom.), 38.20 ppm (CH_2_). Anal. Calcd. for C_16_H_13_N_3_OS_2_ (327.42): C, 58.69; H, 4.00; N, 12.83; S, 19.59% Found: C, 58.66; H, 4.28; N, 12.19; S, 19.89%.

5-(Benzylthio)-*N*-(o-tolyl)-1,3,4-thiadiazole-2-carboxamide (**7b**): White crystals, yield 78%, mp. 211–213 °C; FT-IR (ATR) *δ*_max_: 3345 (NH str.), 3105 (CH_arom_ str.), 2971 (CH_3_ str.), 1662 (C=O str.), 1605 (C=N str.); ^1^H NMR: *δ* 11.05 (s, H, NH), 7.83–7.15 (m, 9H, ArH), 4.67(s, 2H, SCH_2_), 1.21 ppm (s, 3H, CH3); ^13^C NMR: *δ* 171.19 (C=O), 165.87, 156.26 (2C, Thiadiazole), 140.98, 136.40, 133.74, 129.25, 129.11, 128.32, 125.23, 121.25 (Arom.), 38.10 (CH_2_), 23.09 ppm (CH_3_). Anal. Calcd. for C_17_H_15_N_3_OS_2_ (341.45): C, 59.80; H, 4.43; N, 12.31; S, 18.78% Found: C, 59.87; H, 4.33; N, 12.37; S, 18.54%.

5-((2-Oxopropyl) thio)-*N*-phenyl-1,3,4-thiadiazole-2-carboxamide (**8a**): White crystals, yield 93.5%, mp. 217–219 °C; FT-IR (ATR) *δ*_max_: 3332 (NH str.), 3060 (CH_arom_ str.), 2917 (CH_3 alip_ str.), 1710 (C=O str.) 1661 (C=O_amidic_ str.), 1622 (C=N str.); ^1^H NMR: *δ* 11.03 (s, H, NH), 7.83–7.17 (m, 5H, ArH), 4.52 (s, 2H, SCH_2_), 2.31 ppm (s, 3H, CH_3_); ^13^C NMR: *δ* 201.67 (C=O), 170.85(C=O_amidic_), 165.93, 1 56.31 (2C, Thiadiazole), 137.94, 129.25, 125.29, 121.06, 44.64 (CH_2_), 29.18 ppm (CH_3_), Dept-135; 129.34, 125.19, 121.39 (Arom.), 44.59 (SCH_2_), 29.02 ppm (CH_3_). Anal. Calcd. for C_12_H_11_N3O_2_S_2_ (293.36): C, 49.13; H, 3.78; N, 14.32; S, 21.86% Found: C, 49.53; H, 3.68; N, 14.39; S, 21.69%.

5-((2-Oxopropyl) thio)-*N*-(o-tolyl)-1,3,4-thiadiazole-2-carboxamide (**8b**): White powder, yield 81.5%, mp. 205–207 °C; FT-IR (ATR) *δ*_max_: 3325 (NH str.), 3060 (CH_arom_ str.), 2957(CH_3alip_ str.), 1713 (C=O str.), 1667 (C=O_amidic_ str.), 1606 (C=N str.); ^1^H NMR: *δ* 10.61 (s, H, NH), 7.39–7.19 (m, 4H, ArH), 4.52 (s, 2H, SCH_2_), 2.30 (s, 3H, COCH_3_), 2.24 (s, 3H, CH_3_ ArH) ppm; ^13^C NMR: *δ* 201.57 (C=O), 170.71 (C=O_amidic_), 165.69, 156.44, (2C, Thiadiazole), 135.85, 130.93, 127.11, 126.66, 126.64 (Arom.), 44.64 (SCH_2_), 28.93 (COCH_3_), 18.10 ppm (CH_3_ Arom**)**. Anal. Calcd. for C_13_H_13_N_3_O_2_S_2_ (307.39): C, 50.79; H, 4.26; N, 13.67; S, 20.86% Found: C, 50.33; H, 4.87; N, 13.47; S,20.71%.

*N*-(4-methoxyphenyl)-5-((2-oxopropyl) thio)-1,3,4-thiadiazole-2-carboxamide (**8c**): White crystals, yield 72%, mp. 267–269 °C; FT-IR (ATR) *δ*_max_: 3343 (NH), 3067 (CH_arom_), 2949–2854 (CH_2_CH_3alip_), 1710 (C=O), 1661 (C=O_amidic_), 1622 (C=N str.); ^1^H NMR: *δ* 10.07 (s, H, NH), 7.83–7.38 (dd, 4H, ArH, *J* = 8.08 Hz), 4.52 (s, 2H, SCH_2_), 4.09 (s,3H, COCH_3_)), 2.31 ppm (s, 3H, OCH_3_); ^13^C NMR:*δ* 201.67 (C=O), 170.85 (C=O_amidic_), 165.93, 156.31 (2C, Thiadiazole), 137.94, 129.25, 125.29, 121.31 (Arom.), 57.13 **(**OCH_3_**)**, 44.64 (SCH_2_), 29.35 ppm (COCH_3_). Anal. Calcd. for C_13_H_13_N_3_O_3_S_2_ (323.39): C, 48.28; H, 4.05; N, 12.99; S, 19.83% Found: C, 48.65; H, 4.35; N, 12.78; S, 19.48%.

*N*-(4-nitrophenyl)-5-((2-oxopropyl) thio)-1,3,4-thiadiazole-2-carboxamide (**8d**): White crystals, yield 66%, mp. 280–282 °C; FT-IR (ATR) *δ*_max_: 3345 (NH str.), 3191 (CH_arom_ str.), 3104–2921 (CH_2_, CH_3alip_ str.), 1762 (C=O str.), 1678 (C=O_amidic_ str.), 1659 (C=N str.), 1536,1341 (NO_2_ str.); ^1^H NMR: *δ* 11.15 (s, H, NH), 7.89–7.44 (dd, 4H, ArH, *J* = 8.08 Hz), 4.67 (s, 2H, SCH_2_), 2.15 ppm (s, 3H, CH_3_); ^13^C NMR:*δ* 202.02 (C=O), 171.89 (C=O_amidic_), 166.54, 157.13 (2C, Thiadiazole), 138.11, 129.66, 125.51, 121.82 (Arom.), 44.69 (SCH_2_), 29.67 ppm (COCH_3_). Anal. Calcd. for C_12_H_10_N_4_O_4_S_2_ (338.36): C, 42.60; H, 2.98; N, 16.56; S, 18.95% Found: C, 42.63; H, 2.95; N, 16.59; S, 18.94%.

Ethyl 2-((5-(phenylcarbamoyl)-1,3,4-thiadiazol-2-yl) thio) acetate (**9a**): White crystals, yield 64%, mp. 133–13; FT-IR (ATR) *δ*_max_: 3537 (NH_amidic_ str.), 3142 (CH_arom_ str.), 2983–2905 (CH_2_, CH_3alip_ str.), 1737.94 (C=O str.), 1664.28 (C=O_amidic_ str.), 1605 (C=N str.); ^1^H NMR: *δ* 11.05 (s, H, NH), 7.83–7.15 (m, 5H, ArH), 4.35 (s, 2H, SCH_2_), 4.20, 4.18, 4.16, 4.14 (q, 2H, CH_2_, *J* = 7.08 Hz), 1.23, 1.22, 1.20 ppm (s, 3H, CH_3_, *J* = 7.08 Hz); ^13^C NMR: *δ* 170.42 (C=O), 168.08, (C=O _amidic_), 166.38, 156.55 (2C, Thiadiazole), 137.87, 128.85, 125.61, 121.2 (Arom.), 3,62.19, 36.23, 14.34 ppm (CH_3_). Dept-135; 129.31, 125.21, 121.40 (Arom.), 62.12, (SCH_2_), 35.67 (COOCH_2_), 14.56 ppm (CH_3_). Anal. Calcd. for C_13_H_13_N_3_O_3_S_2_ (323.39): C, 48.28; H, 4.05; N, 12.99; S, 19.83% Found: C, 48.50; H, 4.17; N, 12.58; S, 19.48%.

2-((5-(Phenylcarbamoyl)-1,3,4-thiadiazol-2-yl)thio)acetic acid (**10a**): White crystals, yield 90%, mp. 199–201 °C; FT-IR (ATR) *δ*_max_: 3322 (NH_amidic_ str.), 3104 (br OH str.), 3061 (CH_arom_ str.), 2979–2926 (CH_2_CH_3alip_ str.), 1721 (C = O str.) 1665 (C=O_amidic_ str.), 1599 (C=N str.); ^1^H NMR: *δ* 15.11 (s, H, COOH), 11.04 (s, H, NH), 7.81 -7.18, (m, 5H, ArH), 4.52 (s, 2H, SCH_2_) ppm; ^13^C NMR: *δ* 170.63 (C=O), 169.39 (C=O_amidic_), 165.95, 156.33 (2C, Thiadiazole), 137.86, 129.28, 125.36, 121.35 (Arom.), 36.36 ppm (CH_2_). Dept-135; 129.32, 125.50, 121.40 (CH Arom), 36.27 ppm (CH_2_). Anal. Calcd. for C_11_H_9_N_3_O_3_S_2_ (295.34): C, 44.73; H, 3.07; N, 14.23; S, 21.71% Found: C, 44.33; H, 3.37; N, 14.43; S, 21.53%**.**

O-ethyl *S*-(5-(phenylcarbamoyl)-1,3,4-thiadiazol-2-yl) carbonothioate (**11a**): White powder, yield 53%, mp. 166–168 °C; FT-IR (ATR) *δ*_max_: 3349 (NH_amidic_ str.), 3055 (CH_arom_ str.), 2984–2870 (CH_2_CH_3alip_ str.), 1729.32 (C=O str.) 1682 (C=O_amidic_ str.), 1641 (C=N str.); ^1^H NMR: *δ* 11.23 (s, H, NH), 7.82 -7.15 (m, 5H, ArH), 4.76–4.67 (q, 2H, CH_2_, *J* = 10.88 Hz), 1.05–1.00 ppm (t, 3H, CH_3_, *J* = 10.88 Hz); ^13^C NMR: *δ* 168.75 (C=O), 164.29 (C=O), 158.51, 155.86 (2C,Thiadiazole), 137.87, 129.20, 125.20, 121.35 (Arom.), 56.53 (CH_2_), 18.86 ppm (CH_3_). Anal. Calcd. for C_12_H_11_N_3_O_3_S_2_ (309.36): C, 46.59; H, 3.58; N, 13.58; S, 20.73% Found: C, 46.44; H, 3.81; N, 13.78; S, 20.55%.

#### General synthesis of 5-(substituent)-*N*-phenyl-1,3,4-thiadiazole-2-carboxamide derivatives 12a–18a

A mixture of 2-hydrazinyl-*N*-phenyl-2-thioxoacetamide **(1a)** (1.0 mmol) and an aldehyde namely; cinnamaldehyde, *p*-*N, N* dimethylaminobenzaldehyde, 3,4,5-trimethoxy-benzaldehyde, 1-naphthaldehyde, pipreonal, *p* methylbenzaldehyde and glyoxal (1.0 mmol) was refluxed for 3 h. in acetic acid. The solid product was filtrated and crystallized from ethanol, see Figure (S55–S71).

*N*-phenyl-5-styryl-1,3,4-thiadiazole-2-carboxamide (**12a**): Yellow crystals, yield 71%, mp. 226–228 °C FT-IR (ATR) *δ*_max_: 3328 (NH str.), 3108 (CH_arom_ str.), 3057–2923 (CH=CH_alip_ str.), 1685 (C=O str.), 1599 (C=N str.); ^1^H NMR: *δ* 10.70 (s, H, NH, exchangeable by D_2_O), 7.85–7.08 (m, 10H, ArH), 6.68 (s, 2H, CH=CH) ppm; ^13^C NMR: *δ* 167.81 (C=O), 163.20, 158.13 (2C, Thiadiazole), 144.36, 141.98, 139.27, 139.04, 138.66, 133.38, 129.05, 124.05, 120.74, 112.13 (Arom.) ppm. Anal. Calcd. for C_17_H_13_N_3_OS (307.37): C, 66.43; H, 4.26; N, 13.67; S, 10.43% Found: C, 66.38; H, 4.31; N, 13.61; S, 10.45%.

5-[4-(Dimethylamino) phenyl]-*N*-phenyl-1,3,4-thiadiazole-2-carboxamide (**13a**): Red crystals, yield 91%, mp. 210–212 °C FT-IR (ATR) *δ*_max_: 3327 (NH str.), 3087 (CH_arom_ str.), 2983–2874 (CH_3_ str.), 1664 (C=O str.), 1625 (C=N str.); ^1^H NMR: *δ* 11.31 (s, H, NH_amidic_, exchangeable by D_2_O), 8.23–7.09 (m, 9H, ArH), 2.84 (s, 6H, 2CH_3_); ^13^C NMR: *δ* 171.33 (C=O), 166.60, 162.53 (2C, Thiadiazole), 156.24, 137.00, 135.08, 133.21, 129.07, 125.90, 125.34, 121.20 (Arom.), 36.42 ppm (2CH_3_). Anal. Calcd. for C_17_H_16_N_4_OS (324.40): C, 62.94; H, 4.97; N, 17.27; S, 9.88% Found: C, 62.85; H, 4.98; N, 17.29; S, 9.89%.

*N*-Phenyl-5-(3,4,5-trimethoxyphenyl)-1,3,4-thiadiazole-2-carboxamide (**14a**): White crystals, yield 76%, mp. 199–201 °C FT-IR (ATR) *δ*_max_3278 (NH str.), 3146 (CH_arom_ str.), 2998–2874 (OCH_3_ str.), 1671 (C=O str.), 1625 (C=N str.); ^1^H NMR: *δ* 10.21 (s, H, NH, exchangeable by D_2_O), 7.99–7.12 (m, 7H, ArH), 3.94 (s, 9H, 3OCH_3_) ppm; ^13^C NMR: *δ* 173.54 (C=O), 167.53, 162.81 (2C, Thiadiazole), 151.80, 147.37, 135.70, 130.09, 126.28, 124.31, 122.13, 120.55 (Arom.), 56.56, 48.97 (3OCH_3_) ppm. Anal. Calcd. for C1_8_H_17_N_3_O_4_S (371.41): C, 58.21; H, 4.61; N, 11.31; S, 8.63% Found: C, 58.25; H, 4.62; N, 11.28; S, 8.58%.

5-(Naphthalen-1-yl)-*N*-phenyl-1,3,4-thiadiazole-2-carboxamide (**15a**): Pale yellow crystals, yield 67%, mp. 245–247 °C FT-IR (ATR) *δ*_max_: 3317 (NH str.), 3037–2985 (CH_arom_ str.), 1673 (C=O str.), 1625 (C=N str.); ^1^H NMR: *δ* 10.52 (s, H, NH_amidic_, exchangeable by D_2_O), 7.81–7.09 ppm (m, 12H, ArH) ppm; ^13^C NMR: *δ* 171.96 (C=O), 164.76, 156.81 (2C, Thiadiazole), 135.95, 135.38, 133.68, 132.99, 129.43, 129.21, 129.25, 128.36, 128.21, 128.05, 121.29, 120.61, 119.76, 117.78 (Arom.) ppm. Anal. Calcd. For C_19_H_13_N_3_OS (331.39): C, 68.91; H, 3.93; N, 12.64; S, 9.68% Found: C, 68.86; H, 3.95; N, 12.68; S, 9.66%.

5-(Benzo[d][1,3]dioxol-5-yl)-*N*-phenyl-1,3,4-thiadiazole-2-carboxamide (**16a**): White crystals, yield 74%, 225–227 °C FT-IR (ATR) *δ*_max_: 3330 (NH str.), 3059 (CH_arom_ str.), 2962 (CH_2alip_ str.), 1690 (C=O str.), 1622 (C=N str.); ^1^H NMR: *δ* 10.20 (s, H, NH, exchangeable by D_2_O), 7.75–6.93 (m, 8H, ArH), 6.58 (s, 2H, CH_2_ piprenal) ppm; ^13^C NMR: *δ* 171.05 (C=O), 161.88, 158.45 (2C, Thiadiazole), 147.37, 146.15, 141.08, 139.21, 129.71, 127.85, 125.36, 121.57, 133.05, 111.04 (Arom.), 101.04 (CH_2_ piprenal) ppm. Anal. Calcd. for C_16_H_11_N_3_O_3_S (325.34): C, 59.07; H, 3.41; N, 12.92; S, 9.86% Found: C, 59.20; H, 3.38; N, 12.81; S, 9.84%.

*N*-phenyl-5-(p-tolyl)-1,3,4-thiadiazole-2-carboxamide (**17a**): Pale yellow, yield 77%, mp. 233-235°C FT-IR (ATR) *δ*_max_: 3322 (NH str.), 3125 (CH_arom_ str.), 2976 (CH_3alip_ str.), 1677 (C=O str.), 1625 (C=N str.); ^1^H NMR: *δ* 10.20 (s, H, NH, exchangeable by D_2_O), 7.89–7.13 (m, 9H, ArH), 3.41 (s, 3H, CH_3_) ppm; ^13^C NMR: *δ* 172.26 (C=O), 166.02, 156.66 (2C, Thiadiazole), 153.95, 141.17, 138.14, 129.24, 125.22, 124.85, 121.40, 105.40 (Arom.), 19.02 ppm (CH_3_). Anal. Calcd. For C_16_H_13_N_3_OS (295.36): C, 65.06; H, 4.44; N, 14.23; S, 10.86% Found: C, 64.98; H, 4.46; N, 14.21; S, 10.88%.

*N*, *N*-diphenyl-2,2',3,3'-tetrahydro[2,2'-bi(1,3,4-thiadiazole)]-5,5'-dicarboxamide (18a): Orange crystals, yield 81%, mp. 187–189 °C FT-IR (ATR) *δ*_max_3321, 3255 (2NH str.), 3079 (CH_arom_ str.), 2968 (CH_thiadiazole_ str.), 1693 (C=O str.), 1625 (C=N str.); ^1^H NMR: *δ* 10.19 (s, H, NH_amidic_, exchangeable by D_2_O), 9.04 (s, H, NH_thiadiazole_, exchangeable by D_2_O), 7.74–7.06 (m, 5H, ArH), 5.53 (s, 2H, CH_thiadiazole_) ppm; ^13^C NMR: *δ* 159.13 (C=O), 139.04 (C, Thiadiazole), 138.66, 129.05, 124.29, 120.74 (Arom.), 76.13 ppm (CH_thiadiazole_); Dept-135 NMR; 129.09, 129.25, 124.31, 120.74 (Arom.), 75.98 ppm (CH_thiadiazole_) Anal. Calcd. for C_18_H_16_N_6_O_2_S_2_ (412.49): C, 52.41; H, 3.91; N, 20.37; S, 15.55% Found: C, 52.46; H, 3.90; N, 20.39; S, 15.52%.

### Biological evaluation

#### Antimicrobial screening

According to the antibacterial activity of several compounds was screened using the agar well diffusion method [43]. Ciprofloxacin was utilized to compare the results as a positive control. Dimethylsulfoxide (DMSO) solution (10% v/v) was used as a negative control.

#### In-vitro anti-inflammatory activity (protein denaturation) of the tested compounds

For the test compounds and the reference medication, diclofenac sodium, 0.05 mL of various concentrations (50, 100, 150, 200, and 250 µg/ml) were used, respectively. Then all tubes were combined with 0.45 ml (0.5% w/v) of BSA. The samples were heated for 3 min to maintain a temperature of 57 °C after being incubated at 37 °C for 20 min. Add 2.5 ml of phosphate buffer to the aforementioned solutions after cooling. At 660 nm, a UV–Visible spectrophotometer was used to detect the absorbance. Protein denaturation at 100% is represented by the control. A positive control drug called diclofenac sodium was used to compare the outcomes [44]. Calculations can be made to determine the degree of protein denaturation inhibition.

$$\% {\text{ inhibition of denaturation}} = {1}00 \times \left( {{1} - {\text{A2}}/{\text{A1}}} \right)$$

A2 = Absorbance of the test sample, A1 = Absorbance of control.

### Statistical analysis

Analysis was performed using Statistical Program for Social Science (SPSS) version 26 (Armonk, NY: IBM Crop). The gathering of data was recorded and evaluated on an IBM-compatible computer. One-way ANOVA was used to determine if there was any statistically significant difference. P value ≤ 0.05 was considered significant.

## Molecular docking

To predict the binding style and interactions of the aforementioned drugs with dihydropteroate synthase, molecular docking experiments were carried out to better understand their efficacy (DHPS). This last one is a crucial enzyme in the prokaryotic biosynthesis of folic acid and a crucial cofactor in the pathways that almost all bacterial strains use to synthesize nucleic acids, making it a prime candidate for antibiotics [45, 46]. Thus, crystal structure of DHPS in complex with pterin-sulfonamide conjugates [47]. PDB ID 6CLV, from *S. aureus* organism, was employed as a binding site in molecular docking simulations and downloaded from the RCSB protein data library. The docking results showed strong interactions with high docking scores (S) (more negative) of studied compounds to DHPS from *S. aureus*. The negative values of the calculated docking scores (S) for studied compounds, Table 3, demonstrates that the binding is spontaneous, and the chemicals are suitable for use as drugs [48, 49].

**Fig. 5:**
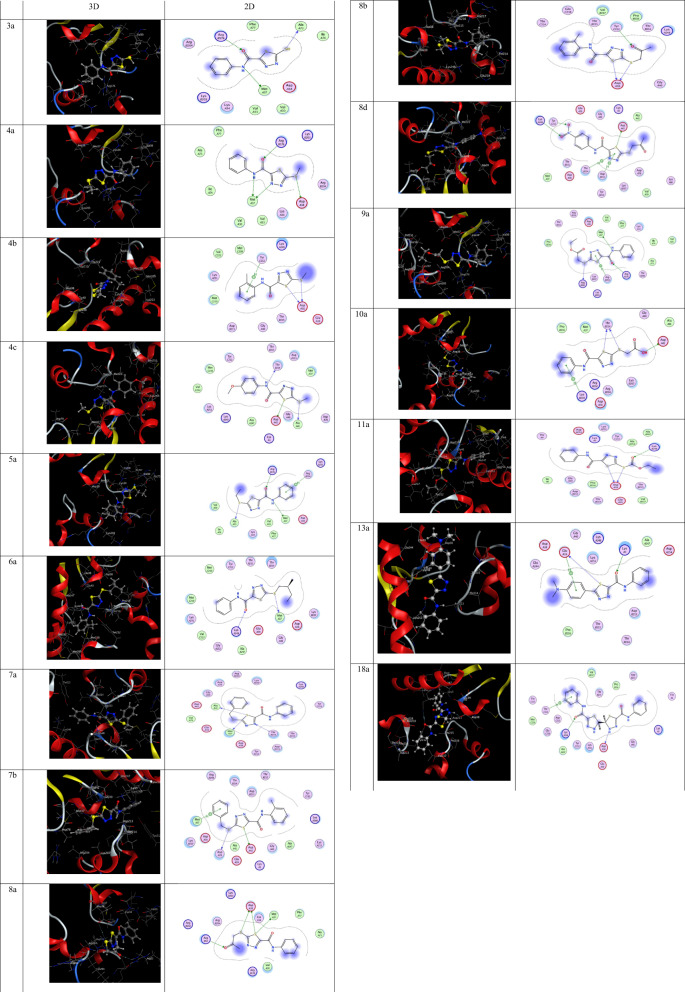
2D and 3D views of the docked compounds with DHPS

The subject compounds had a strong docking score from − 6.821 **(3a)**, − 6.814 **(8d),** − 6.809 **(4c),** and − 6.498 **(4a),** to − 5.560 **(10a),** and − 5.463 **(4b)** Kcal/mol toward the DHPS from *S. aureus* as can be shown from (Table 3). Due to their high docking score, **3a, 8d** and **4c** seem to be the most active. Compound **3a** revealed three hydrogen bonds interactions between N 7 with MET 37, S 15 with ALA 73, and O 9 with ARG 176; and **8d** revealed three hydrogen bonds interactions between S 11 with ASP 42, O 21 with TYR 212, and O 22 with LYS 248; furthermore, **4c** revealed three hydrogen bonds interactions between N 7 with THR 214, S 11 with ASP 42, S 15 with ALA 41. The docking results showed good interactions of the investigated **13a**, and **18a** compounds to DHPS from *S. aureus* (PDB ID: 6CLV). The subject **13a**, and **18a** compounds had good docking scores as − 6.670 kcal/mol, and − 7.380 kcal/mol, respectively. The **13a** revealed one H-donor, one H-acceptor, and one pi-H interactions between S14-GLU39, O10-LYS3, and 6-ring-GLU39, with distance of 3.22, 3.12, and 4.18 Angstrom, respectively, While, **18a** revealed one H-donor, one H-acceptor, and one pi-H interactions between N9-ASP38, O22-LYS248, and 6-ring-THR214, with distance of 2.91, 2.93, and 4.12 Angstrom, respectively (Fig. 5).

**Table 3:** Docking data

	Ligand	Receptor	Interaction	Distance	E (kcal/mol)	S (kcal/mol)
5a	N 7	MET 37	H-donor	3.880	− 1.090	− 5.783
	S 15	ALA 73	H-donor	3.300	− 1.190	
	O 9	ARG 176	H-acceptor	2.430	− 4.100	
	6-ring	ARG 204	pi-H	3.420	− 1.080	
8a	S 11	MET 37	H-donor	3.730	− 1.130	− 5.809
	S 11	ASP 38	H-donor	3.990	− 1.160	
	S 11	ASP 38	H-donor	3.640	− 1.200	
	S 15	ASP 38	H-donor	3.410	− 1.290	
	S 15	ASP 38	H-donor	3.440	− 1.130	
	O 18	ARG 52	H-acceptor	2.750	− 1.340	
10a	S 11	THR 214	H-donor	2.920	− 1.720	− 5.560
	S 15	THR 214	H-donor	3.230	− 1.530	
	O 19	ASP 42	H-donor	2.500	− 5.910	
	6-ring	LYS 203	pi-H	3.330	− 1.700	
9a	N 7	MET 37	H-donor	3.210	− 2.220	− 5.616
	S 15	ARG 202	H-donor	3.530	− 1.510	
	O 9	ARG 176	H-acceptor	2.630	− 2.300	
	5-ring	ARG 204	pi-H	3.880	− 1.380	
	5-ring	ARG 204	pi-H	3.030	− 1.160	
7a	S 11	MET 37	H-donor	2.610	− 1.960	− 6.098
	S 11	ALA 41	H-donor	3.160	− 1.710	
	N 14	THR 214	H-acceptor	2.700	− 2.800	
11a	S 11	ASP 38	H-donor	3.640	− 1.140	− 6.368
	S 15	ASP 38	H-donor	2.730	− 1.390	
	O 18	LYS 248	H-acceptor	3.040	− 1.700	
8b	S 11	ASP 38	H-donor	2.710	− 2.200	− 6.431
	S 15	ASP 38	H-donor	3.010	− 1.600	
	O 19	TYR 212	H-acceptor	2.920	− 1.620	
8d	S 11	ASP 42	H-donor	2.750	− 2.540	− 6.814
	O 21	TYR 212	H-acceptor	2.540	− 1.810	
	O 22	LYS 248	H-acceptor	3.060	− 1.630	
	5-ring	ASP 213	pi-H	3.320	− 1.850	
	5-ring	THR 214	pi-H	3.990	− 1.140	
7b	S 11	ASP 42	H-donor	2.750	− 1.830	− 6.315
	S 15	ASP 78	H-donor	3.950	− 1.510	
	6-ring	MET 37	pi-H	3.690	− 0.600	
4b	S 11	ASP 38	H-donor	2.790	− 1.130	− 5.463
	S 15	ASP 38	H-donor	3.080	− 1.400	
	6-ring	TYR 212	pi-H	3.730	− 1.170	
6a	S 15	MET 37	H-donor	2.320	− 1.140	− 5.817
	O 9	LYS 248	H-acceptor	2.840	− 1.600	
4a	N 7	MET 37	H-donor	3.120	− 2.800	− 6.498
	N 14	MET 37	H-donor	3.160	− 1.130	
	S 15	ASP 38	H-donor	3.560	− 1.810	
	S 15	ASP 38	H-donor	3.570	− 0.740	
	O 9	ARG 176	H-acceptor	2.710	− 1.820	
3a	N 7	MET 37	H-donor	3.860	− 1.350	− 6.821
	S 15	ALA 73	H-donor	3.160	− 1.300	
	O 9	ARG 176	H-acceptor	2.560	− 2.600	
4c	N 7	THR 214	H-donor	2.740	− 1.560	− 6.809
	S 11	ASP 42	H-donor	2.760	− 2.430	
	S 15	ALA 41	H-donor	2.830	− 1.980	
13a	S 14	GLU39	H-donor	3.22	− 0.80	− 6.670
	O 10	LYS3	H-acceptor	3.12	− 1.40	
	6-ring	GLU39	pi-H	4.18	− 0.60	
18a	N 9	ASP38	H-donor	2.91	− 1.60	− 7.380
	O 22	LYS248	H-acceptor	2.93	− 7.60	
	6-ring	THR214	pi-H	4.12	− 0.60

### Supplementary Information

Supplementary Material 1.

## Data Availability

The authors declare that the data supporting the findings of this study are available within the paper and its Supplementary Information files. Should any raw data files be needed in another format they are available from the corresponding author upon reasonable request.
