# Supplementary material for: Synthesis, docking and characterization of some novel 5-(S-alkyl)-1.3.4-thiadiazole-2-carboxamide derivatives as anti-inflammatory and antibacterial agents
Source: BMC Chem. 2024 Jul 27;18(1):138. doi: 10.1186/s13065-024-01237-9 (PMC11282722; doi:10.1186/s13065-024-01237-9)
Supplement: Supplementary file 1 — Supplementary Material 1. [file 13065_2024_1237_MOESM1_ESM.docx]

**Synthesis, Docking and Characterization of Some Novel 5-(S-Alkyl)-1.3.4-thiadiazole-2-carboxamide Derivatives as Anti-inflammatory and Antibacterial Agents**

**Ahmed M. El-Saghier*^1^, Asmaa Abdul-Baset^1^, Omer M. El-Hady^1^, Walaa M. Abd El-Raheem^2^ , Asmaa M. Kadry^1^**

*^1^Chemistry Department, Faculty of Science, Sohag University, Sohag 82524, Egypt*

*^2^Botany and Microbiology Department, Faculty of Science, Sohag University, Sohag 82524, Egypt*

*Corresponding author Email:* [*el.saghier@science.sohag.edu.eg*](mailto:el.saghier@science.sohag.edu.eg)

**Table S1: Antibacterial activity of different concentrations of the tested compounds against the human pathogenic bacteria.**

| Tested compound | Conc.  mg/ml | The size of inhibition zone (diameter in mm) | | | |
| --- | --- | --- | --- | --- | --- |
|  |  | **(Gram +Ve )** | | **(Gram -Ve )** | |
|  |  | ***S. aureus*** | ***Bacillus sp.*** | ***K. pneumoniae*** | ***E. coli*** |
| 3a | 0.1 | 24 | 0 | 12 | 0 |
|  | 0.2 | 28 | 10 | 13 | 0 |
|  | 0.3 | 31 | 12 | 15 | 0 |
|  | 0.4 | 35 | 15 | 17 | 0 |
|  | 0.5 | 39 | 19 | 18 | 0 |
| 3b | 0.1 | 25 | 12 | 13 | 0 |
|  | 0.2 | 28 | 16 | 16 | 0 |
|  | 0.3 | 30 | 22 | 17 | 0 |
|  | 0.4 | 32 | 25 | 18 | 0 |
|  | 0.5 | 34 | 27 | 23 | 0 |
| 3c | 0.1 | 19 | 0 | 12 | 0 |
|  | 0.2 | 22 | 10 | 13 | 0 |
|  | 0.3 | 24 | 12 | 15 | 0 |
|  | 0.4 | 29 | 16 | 15 | 0 |
|  | 0.5 | 31 | 19 | 18 | 9 |
| 3d | 0.1 | 14 | 10 | 12 | 0 |
|  | 0.2 | 21 | 15 | 15 | 0 |
|  | 0.3 | 23 | 16 | 20 | 0 |
|  | 0.4 | 25 | 25 | 25 | 0 |
|  | 0.5 | 26 | 28 | 27 | 0 |
| 4a | 0.1 | 27 | 10 | 11 | 0 |
|  | 0.2 | 28 | 12 | 15 | 0 |
|  | 0.3 | 31 | 16 | 20 | 0 |
|  | 0.4 | 33 | 20 | 25 | 0 |
|  | 0.5 | 35 | 23 | 27 | 0 |
| 5a | 0.1 | 12 | 0 | 0 | 0 |
|  | 0.2 | 15 | 9 | 12 | 0 |
|  | 0.3 | 17 | 11 | 14 | 0 |
|  | 0.4 | 21 | 13 | 17 | 0 |
|  | 0.5 | 23 | 16 | 21 | 0 |
| 8a | 0.1 | 12 | 13 | 0 | 0 |
|  | 0.2 | 17 | 18 | 16 | 0 |
|  | 0.3 | 19 | 20 | 18 | 0 |
|  | 0.4 | 20 | 26 | 20 | 0 |
|  | 0.5 | 24 | 29 | 22 | 0 |
| 10a | 0.1 | 10 | 0 | 0 | 0 |
|  | 0.2 | 12 | 16 | 0 | 0 |
|  | 0.3 | 15 | 19 | 0 | 0 |
|  | 0.4 | 16 | 20 | 0 | 0 |
|  | 0.5 | 17 | 22 | 0 | 0 |
| 9a | 0.1 | 0 | 0 | 0 | 0 |
|  | 0.2 | 12 | 12 | 0 | 0 |
|  | 0.3 | 14 | 14 | 9 | 0 |
|  | 0.4 | 17 | 14 | 11 | 0 |
|  | 0.5 | 19 | 13 | 12 | 0 |
| 7a | 0.1 | 17 | 16 | 16 | 0 |
|  | 0.2 | 23 | 16 | 19 | 0 |
|  | 0.3 | 25 | 18 | 20 | 0 |
|  | 0.4 | 27 | 20 | 22 | 0 |
|  | 0.5 | 27 | 22 | 23 | 1.0 |
| 11a | 0.1 | 13 | 11 | 0 | 0 |
|  | 0.2 | 18 | 12 | 12 | 0 |
|  | 0.3 | 20 | 15 | 14 | 0 |
|  | 0.4 | 26 | 19 | 14 | 0 |
|  | 0.5 | 29 | 22 | 16 | 0 |
| 4c | 0.1 | 22 | 25 | 15 | 0 |
|  | 0.2 | 29 | 27 | 17 | 0 |
|  | 0.3 | 31 | 33 | 19 | 0 |
|  | 0.4 | 34 | 35 | 20 | 9 |
|  | 0.5 | 35 | 37 | 24 | 12 |
| 8b | 0.1 | 19 | 19 | 0 | 0 |
|  | 0.2 | 24 | 22 | 10 | 0 |
|  | 0.3 | 27 | 24 | 14 | 0 |
|  | 0.4 | 30 | 25 | 20 | 0 |
|  | 0.5 | 34 | 26 | 23 | 0 |
| 8c | 0.1 | 27 | 30 | 17 | 0 |
|  | 0.2 | 29 | 34 | 20 | 0 |
|  | 0.3 | 33 | 37 | 26 | 8 |
|  | 0.4 | 38 | 39 | 31 | 10 |
|  | 0.5 | 39 | 41 | 36 | 12 |
| 7b | 0.1 | 9 | 10 | 9 | 0 |
|  | 0.2 | 14 | 16 | 12 | 0 |
|  | 0.3 | 20 | 19 | 16 | 0 |
|  | 0.4 | 26 | 20 | 20 | 0 |
|  | 0.5 | 29 | 22 | 19 | 0 |
| 6a | 0.1 | 12 | 24 | 0 | 0 |
|  | 0.2 | 15 | 29 | 0 | 0 |
|  | 0.3 | 20 | 33 | 0 | 0 |
|  | 0.4 | 25 | 35 | 0 | 0 |
|  | 0.5 | 27 | 38 | 0 | 0 |
| 13a | 0.1 | 0 | 9 | 0 | 0 |
|  | 0.2 | 0 | 10 | 0 | 0 |
|  | 0.3 | 0 | 12 | 0 | 0 |
|  | 0.4 | 9 | 18 | 0 | 0 |
|  | 0.5 | 12 | 19 | 0 | 0 |
| 18a | 0.1 | 9 | 10 | 9 | 0 |
|  | 0.2 | 11 | 12 | 12 | 0 |
|  | 0.3 | 16 | 15 | 10 | 0 |
|  | 0.4 | 18 | 15 | 23 | 0 |
|  | 0.5 | 16 | 17 | 30 | 27 |
| Ciprofloxacin | **0.1** | **39** | **25** | **27** | **31** |
|  | **0.2** | **40** | **28** | **30** | **37** |
|  | **0.3** | **43** | **31** | **37** | **42** |
|  | **0.4** | **48** | **36** | **44** | **53** |
|  | **0.5** | **49** | **45** | **52** | **61** |

**Spectrum data of new synthesized compounds (3-18):**

***N*-phenyl-5-thioxo-4,5-dihydro-1,3,4-thiadiazole-2-carboxamide (3a)***:*

White crystals, yield 93%, mp. 180-182 ºC; FT-IR (ATR) δmax: 3345, 3191 (2NH str.), 3104 (CH_arom_ str.), 1678 (C=O str.), 1659 (C=N str.), 1236 (C=S str.); 1H NMR: δ 15.06 (s, H, NHthiadiazole, exchangeable by D_2_O), 10.79 (s, H, NH, exchangeable by D_2_O), 7.77-7.14 (m, 5H, ArH) ppm; ^13^C NMR: *δ* 190.78 (C=S), 157.25 (C=O), 155.34, 137.91 (2C, Thiadiazole), 129.20, 125.23, 121.34 ppm (Arom.). Anal. Calcd. for C_9_H_7_N_3_OS_2_ (237.30): C, 45.55; H, 2.91; N, 17.71; S, 27.02% Found: C, 45.65; H, 2.81; N, 17.61; S, 27.12%.


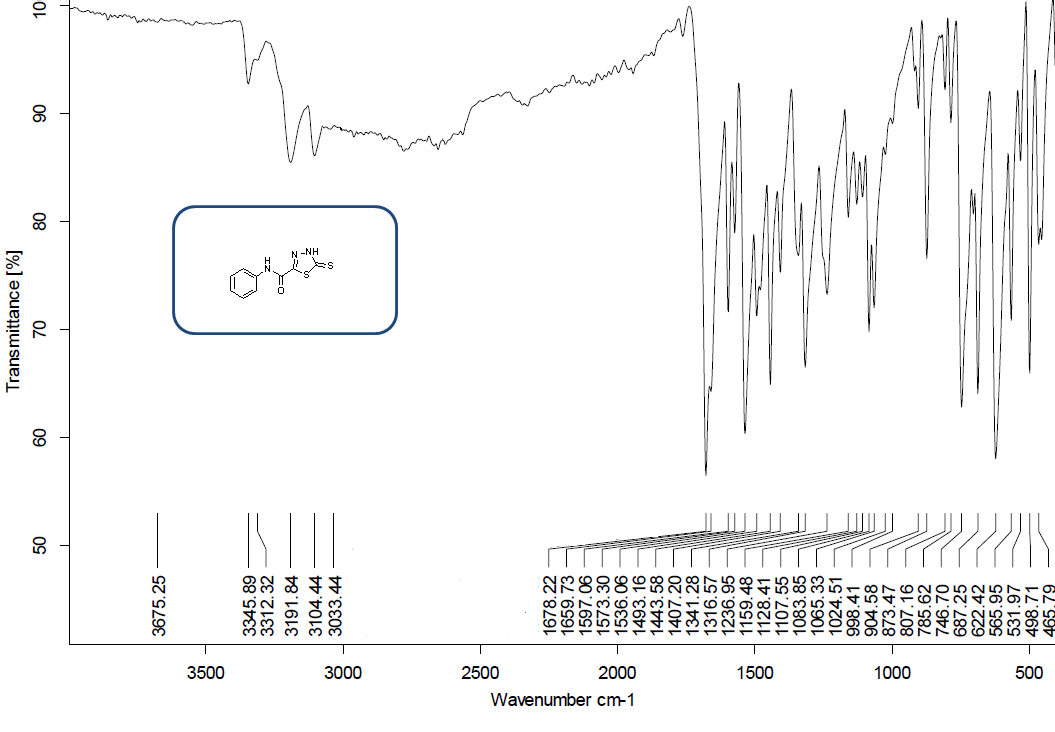


Figure S1


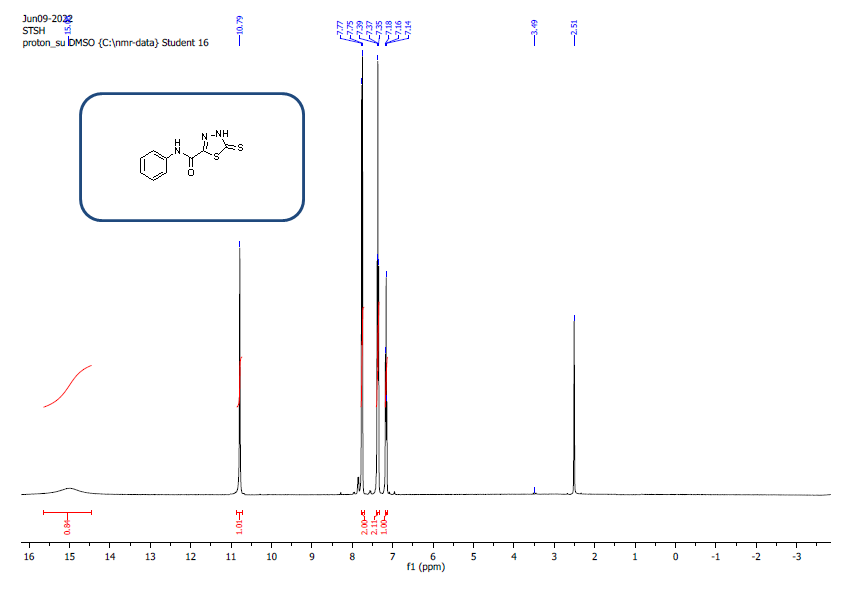


Figure S2


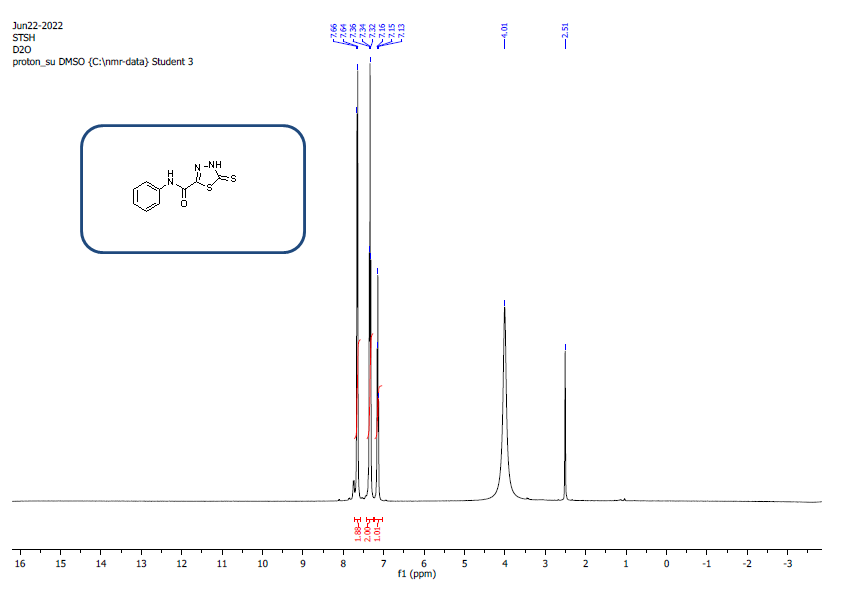


Figure S3


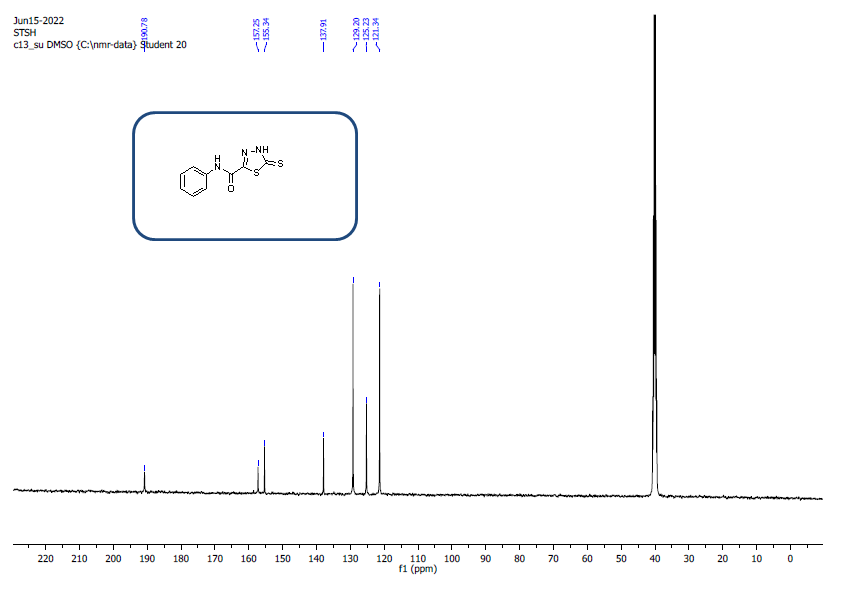


Figure S4

**5-Thioxo-*N*-(o-tolyl)-4,5-dihydro-1,3,4-thiadiazole-2-carboxamide (3b):**

Orange crystals, yield 91%, mp. 177-179 ºC; FT-IR (ATR) *δ*_max_: 3342, 3311 (2NH str.), 3057 (CH_arom_ str.), 1681.12 (C=O str.), 1648 (C=N str.), 1222 (C=S str.); 1H NMR: δ 15.06 (s, H, NH_thiadiazole_), 10.79 (s, H, NH), 7.39-7.19 (m, 4H, ArH), 2.24 ppm (s, 3H, CH3); 13C NMR: δ 190.55 (C=S), 157.25 (C=O), 155.15, 137.91 (2C, Thiadiazole), 134.62, 132.10, 129.20, 125.23, 121.34 (Arom.), 18.54 ppm (CH_3_). Anal. Calcd. for C_10_H_9_N_3_OS_2_ (251.33): C, 47.79; H, 3.61; N, 16.72; S, 25.52% Found: C, 47.35; H, 3.95; N, 16.52; S, 25.31%.


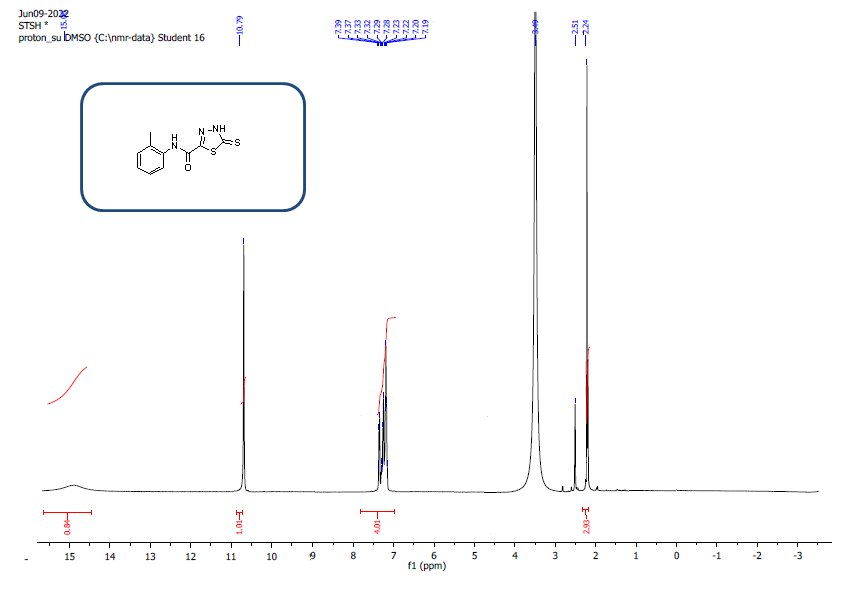


Figure S5


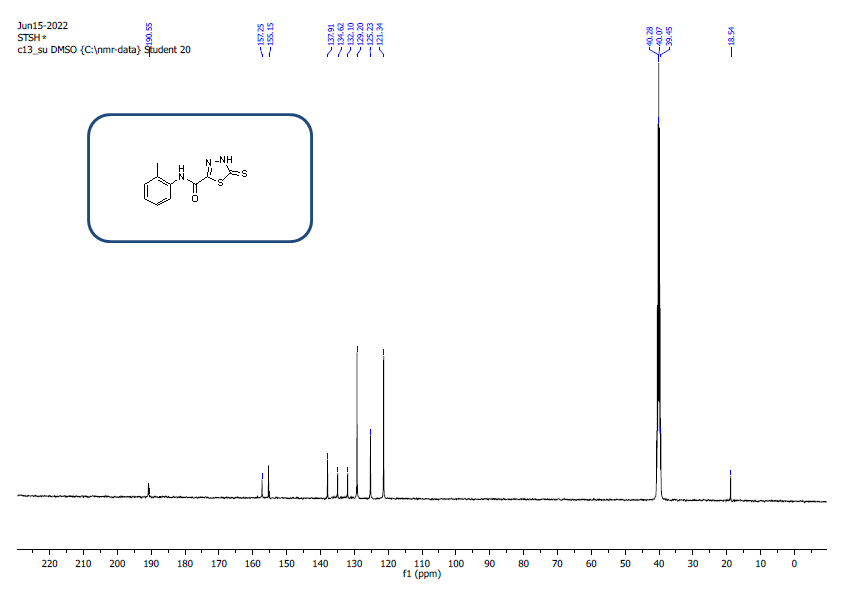


Figure S6

***N*-(4-methoxyphenyl)-5-thioxo-4,5-dihydro-1,3,4-thiadiazole-2-carboxamide (3c):**

Orange crystals, yield 88%, mp. 193-195 ºC; FT-IR (ATR) *δ*_max_: 3348, 3316 (2NH str.), 3132 (CH_arom_ str.), 1675 (C=O str.), 1659 (C=N str.), 1236 (C=S str.); ^1^H NMR: *δ* 15.06 (s, H, NH_thiadiazole_), 10.79 (s, H, NH), 7.77-7.35 (dd, 4H, ArH, *J*= 8.08Hz), 4.18 (s, 3H, OCH_3_) ppm; ^13^C NMR: *δ* 201.67 (C=S), 170.85 (C=O), 165.93 (C, Thiadiazole) 137.94, 129.25, 125.29, 121.31 (Arom.), 54.21 **(**OCH_3_**)** ppm.Anal. Calcd. for C_10_H_9_N_3_O_2_S_2_ (267.33): C, 44.93; H, 3.39; N, 15.72; S, 23.99% Found: C, 44.57; H, 3.75; N, 16.30; S, 23.58%.

**
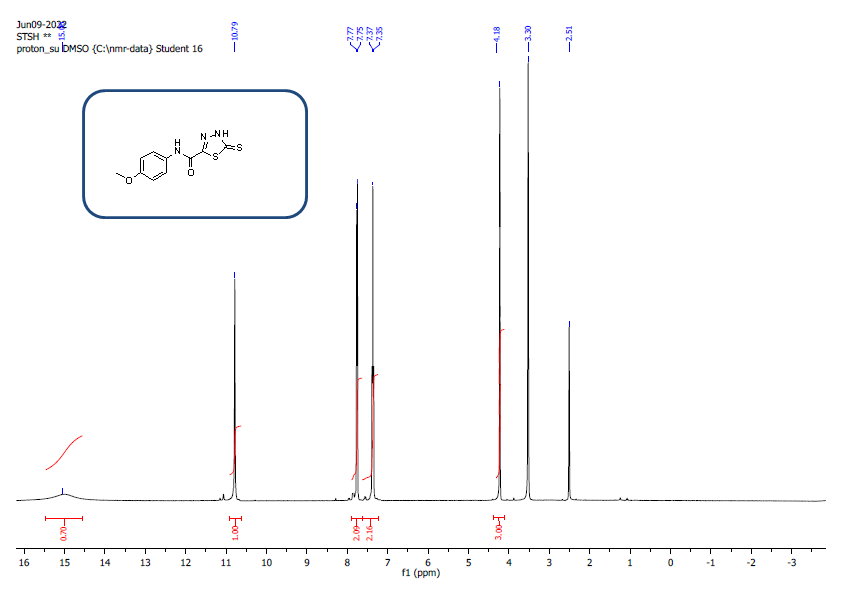
**

Figure S7

**
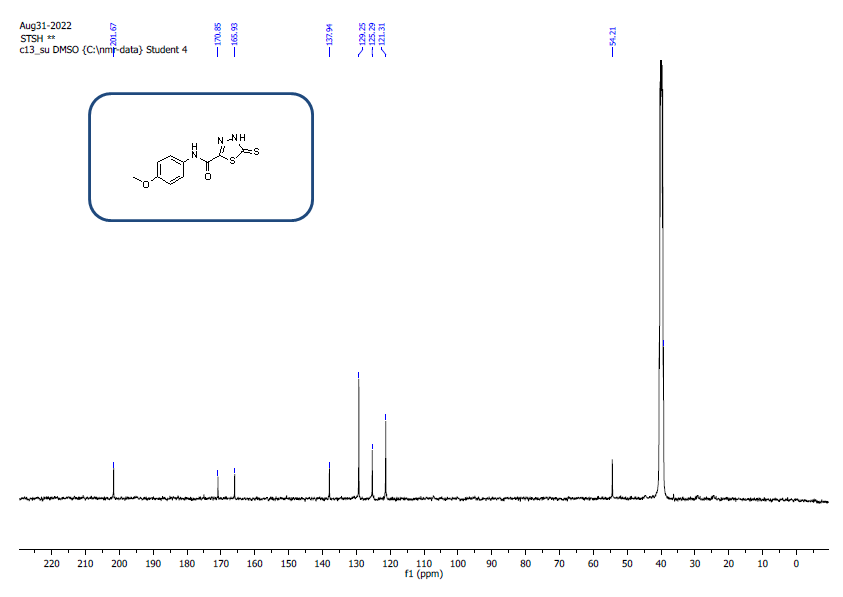
**

Figure S8

***N*-(4-nitrophenyl)-5-thioxo-4,5-dihydro-1,3,4-thiadiazole-2-carboxamide (3d):**

Orange crystals, yield 70%, mp. 201-203 ºC; FT-IR (ATR) *δ*_max_: 3351, 3327 (2NH str.), 3136 (CH_arom_ str.), 1678 (C=O str.), 1666 (C=N str.), 1350, 1555 (NO_2_ str.), 1225 (C=S str.); ^1^H NMR: *δ* 15.01 (s, H, NH_thiadiazole_), 10.79 (s, H, NH), 7.77-7.35 ppm (dd, 4H, ArH, *J*= 8.08Hz); ^13^C NM: *δ* 190.78 (C=S), 157.25 (C=O), 155.15 (C, Thiadiazole) 144.07, 129.25, 129.20, 125.23, 121.34 ppm (Arom.) Anal. Calcd. for C_9_H_6_N_4_O_3_S_2_ (282.30): C, 38.29; H, 2.14; N, 19.85; S, 22.72% Found: C, 38.17; H, 2.24; N, 19.15; S, 22.52%.


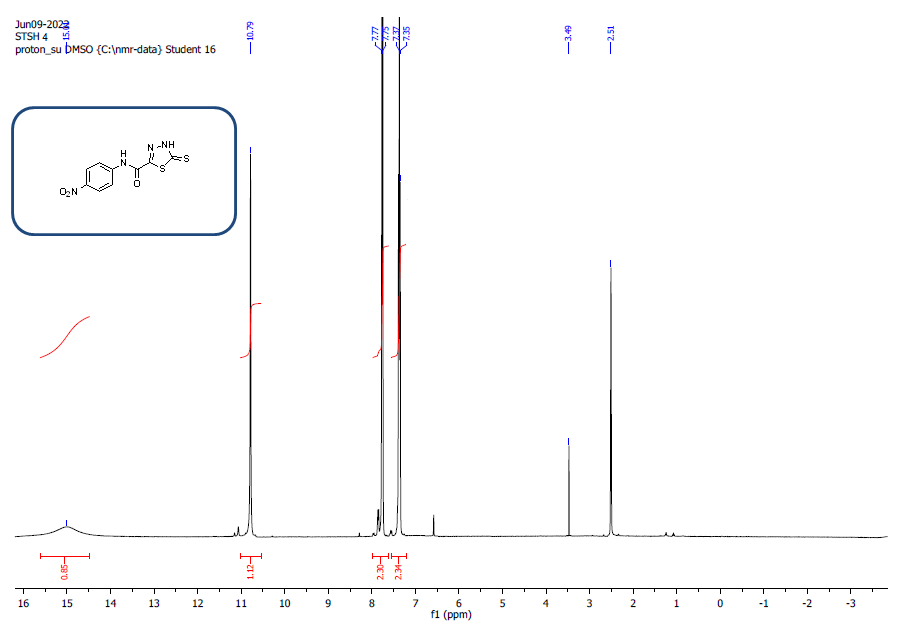


Figure S9


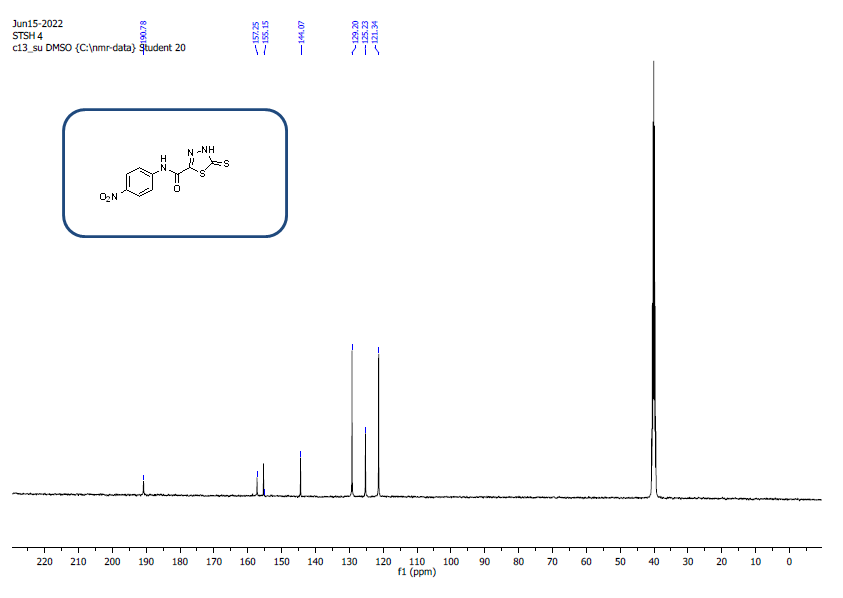


Figure S10

**5-(Methylthio)-*N*-phenyl-1,3,4-thiadiazole-2-carboxamide (4a):**

White crystals, yield 97%, mp. 160-162 ºC; FT-IR (ATR) *δ*_max_: 3381 (NH str.), 3156 (CH_arom_ str.), 2923 (CH_3 alip_ str.), 1673 (C=O str.), 1659 (C=N str.); ^1^H NMR: *δ* 11.03 (s, H, NH), 7.85-7.14 (m, 5H, ArH), 2.83 ppm (s, 3H, SCH_3_); ^13^C NMR: *δ* 173.51 (C=O), 165.29, 156.32 (2C, Thiadiazole), 138.12, 129.18, 125.11, 121.27 (Arom.), 17.37 ppm (SCH_3_). Anal. Calcd. for C_10_H_9_N_3_OS_2_ (251.33): C, 47.79; H, 3.61; N, 16.72; S, 25.52% Found: C, 47.99; H, 3.41; N, 16.42; S, 25.80%**.**

**
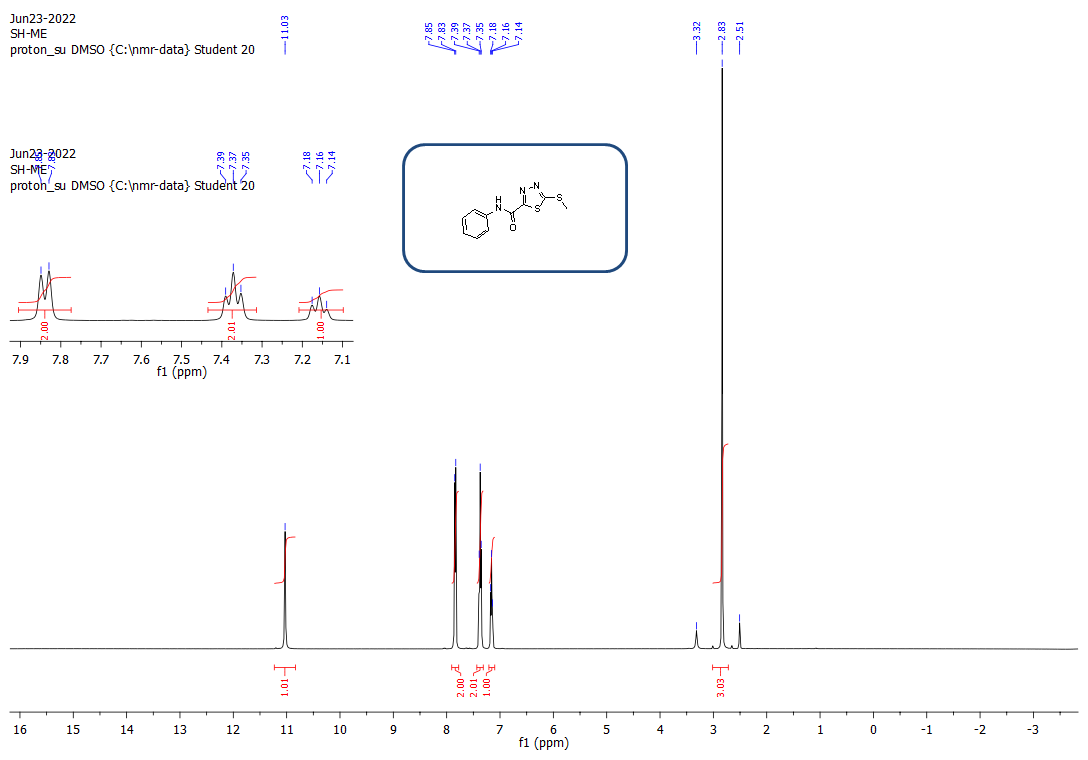
**

Figure S11**
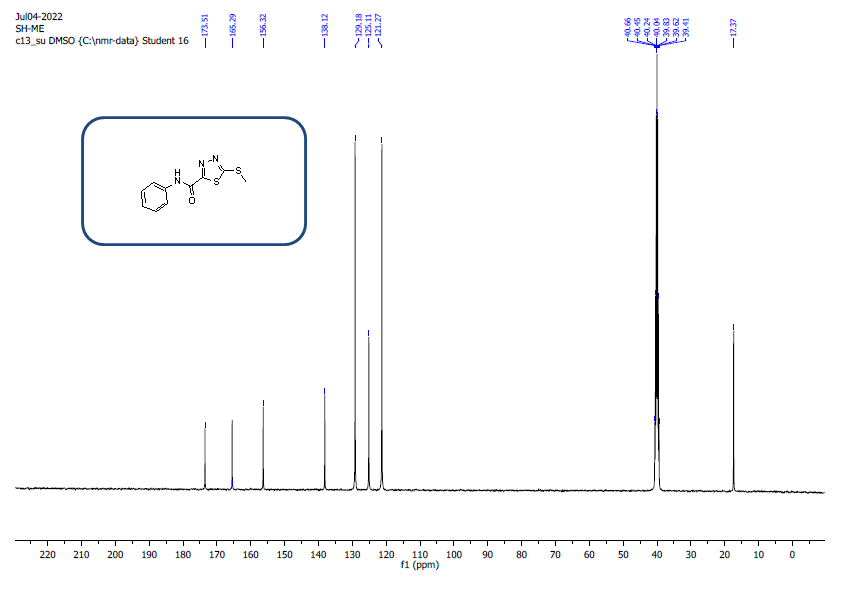
**

Figure S12

**5-(Methylthio)-N-(o-tolyl)-1,3,4-thiadiazole-2-carboxamide (4b):**

White crystals, yield 87%, mp. 155-157 ºC; FT-IR (ATR) *δ*_max_: 3383 (NH str.), 3107 (CH_arom_ str.), 2988, 2963 (CH_3_ str.), 1679 (C=O str.), 1609 (C=N str.); ^1^H NMR: *δ* 10.60 (s, H, NH), 7.39-7.19 (m, 4H, ArH ), 2.30 (s, 3H, CH_3_ Arom), 2.24 (s, 3H, SCH_3_) ppm; ^13^C NMR: *δ* 170.71 (C=O), 165.69, 156.44 (2C, Thiadiazole), 135.25, 134.08, 130.93, 127.11, 126.64, 125.23 (Arom.), 28.93 (SCH_3_), 25.28 **)** ppm (CH_3_ Arom.). Anal. Calcd. for C_11_H_11_N_3_OS_2_ (265.35): C, 49.79; H, 4.18; N, 15.84; S, 24.17% Found: C, 49.63; H, 4.22; N, 15.76; S, 24.16%.

**
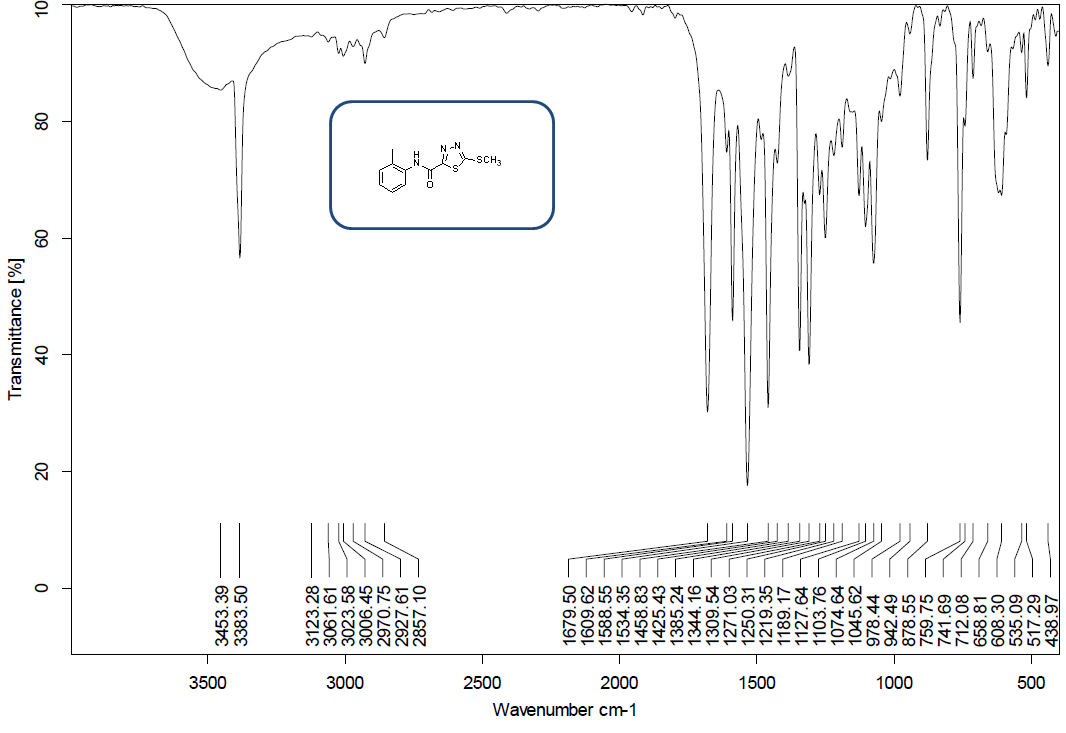
**

Figure S13

**
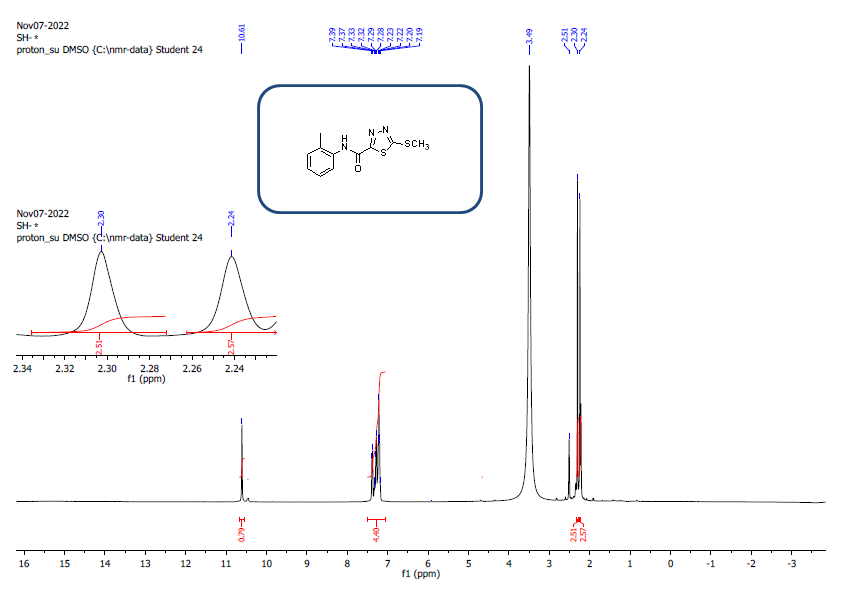
**

Figure S14

***N*-(4-methoxyphenyl)-5-(methylthio)-1,3,4-thiadiazole-2-carboxamide (4c):**

Orange crystals, yield 66%, mp. 198-200 ºC; FT-IR (ATR) *δ*_max_: 3379 (NH str.), 3251 (CH_arom_ str.), 2996, 2985 (CH_3alip_ str.), 1675 (C=O str.), 1605 (C=N str.); ^1^H NMR: *δ* 11.07 (s, H, NH), 7.83-7.38 (dd, 4H, ArH, *J*= 8.08Hz), 4.04 (s, 3H, SCH_3_), 2.31 ppm (s, 3H, SCH_3_); ^13^C NMR: *δ* 170.85, 165.93, 155.15, 137.94, 129.25, 125.29, 121.31 Arom, 54.21 **(**OCH_3_**)**, 24.64 ppm **(**SCH_3_**)**. Anal. Calcd. for C_11_H_11_N_3_O_2_S_2_ (281.35): C, 46.96; H, 3.94; N, 14.93; S, 22.79% Found: C, 46.77; H, 3.89; N, 15.03; S, 22.64%**.**

**
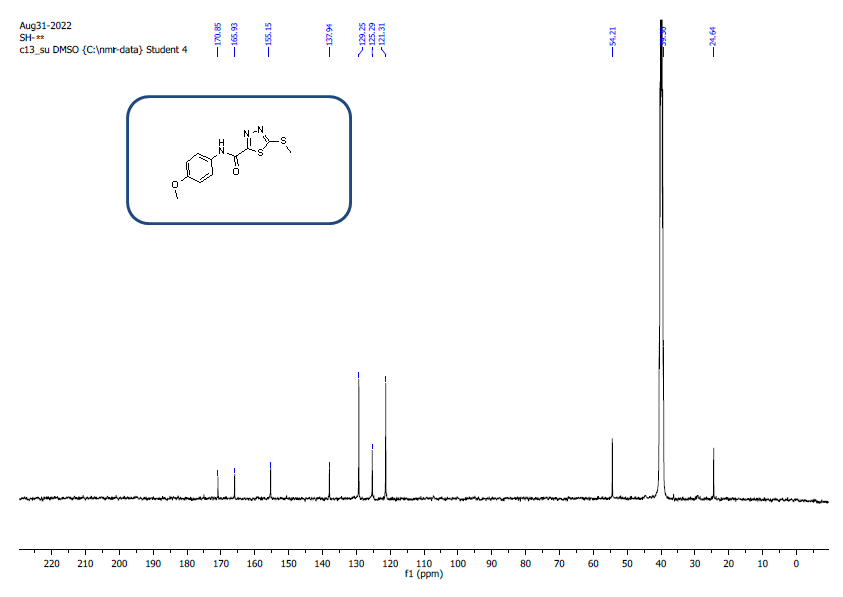
**

Figure S **15
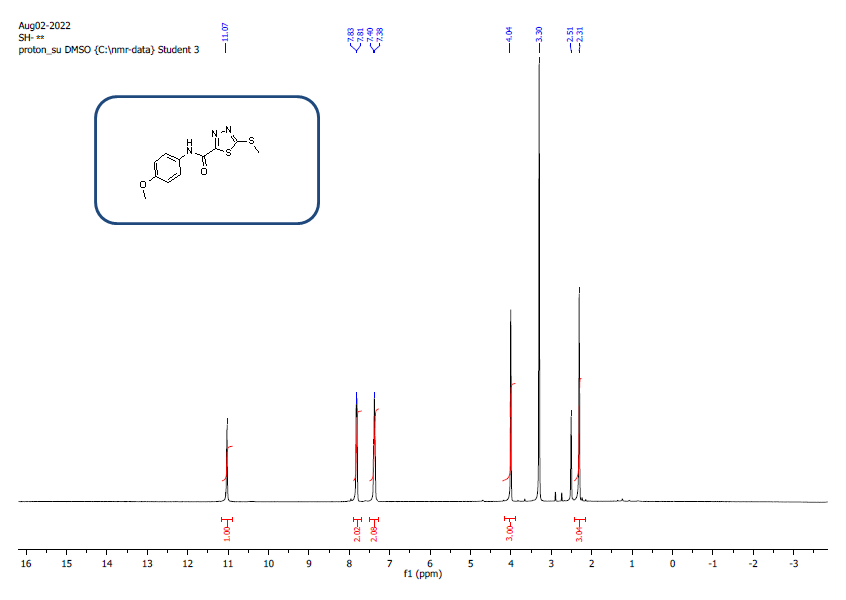
**

Figure S16

**5-(Ethylthio)-N-phenyl-1,3,4-thiadiazole-2-carboxamide (5a):**

White crystals, yield 73%, mp. 157-159 ºC; FT-IR (ATR) *δ*_max_: 3336.85 (NH str.), 3061 (CH_arom_ str.), 2979-2870 (CH_2_CH_3alip_ str.), 1670 (C=O_amidic_ str.),1599 (C=N str.); ^1^H NMR: *δ* 11.04 (s, H, NH), 7.84-7.15 (m, 5H, ArH ), 3.41, 3.40, 3.38, 3.36 (q, 2H, SCH_2_, *J*= 6.6 Hz), 1.45, 1.43, 1.41 ppm (t, 3H, CH_3_, *J* = 6.6 Hz); ^13^C NM: *δ* 171.47 (C=O), 165.37, 156.31 (2C, Thiadiazole), 138.06, 131.87, 125.18, 121.28 (Arom.), 29.18 (CH_2_), 14.64 ppm (CH_3_). Dept -135 NMR; 129.35,124.90,121.31 Arom, 29.36, 14.57 ppm. Anal. Calcd. for C_11_H_11_N_3_OS_2_ (265.03): C, 49.79; H, 4.18; N, 15.84; S, 24.17% Found: C, 49.39; H, 4.18; N, 15.98; S, 24.03%.


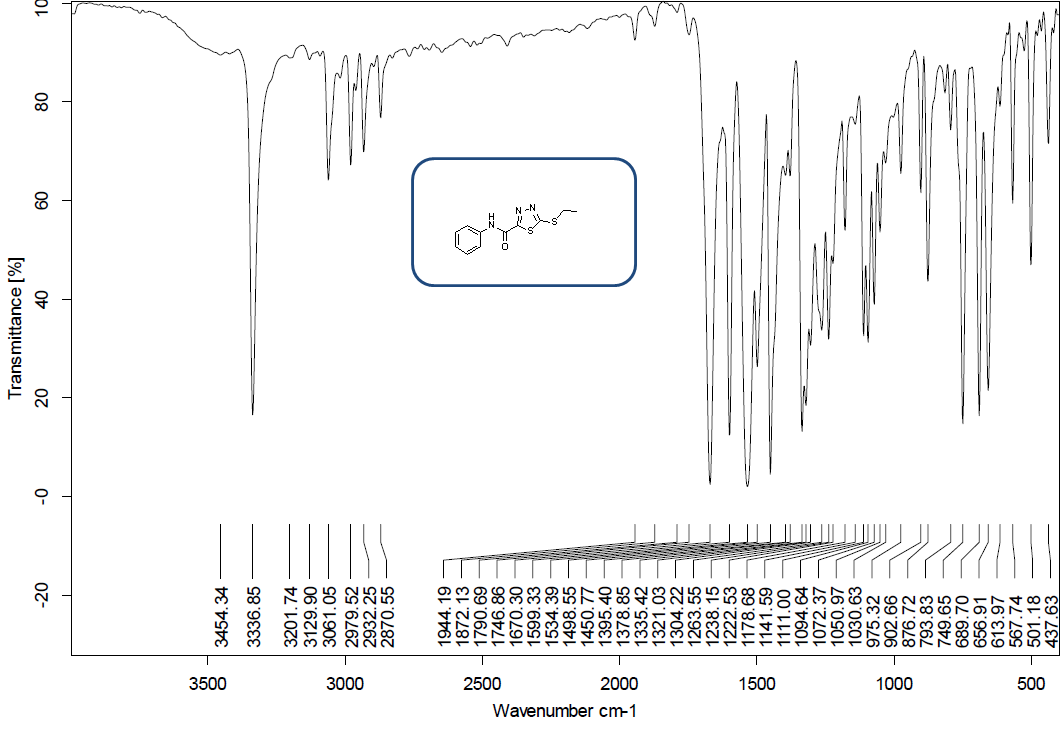


Figure S17

**
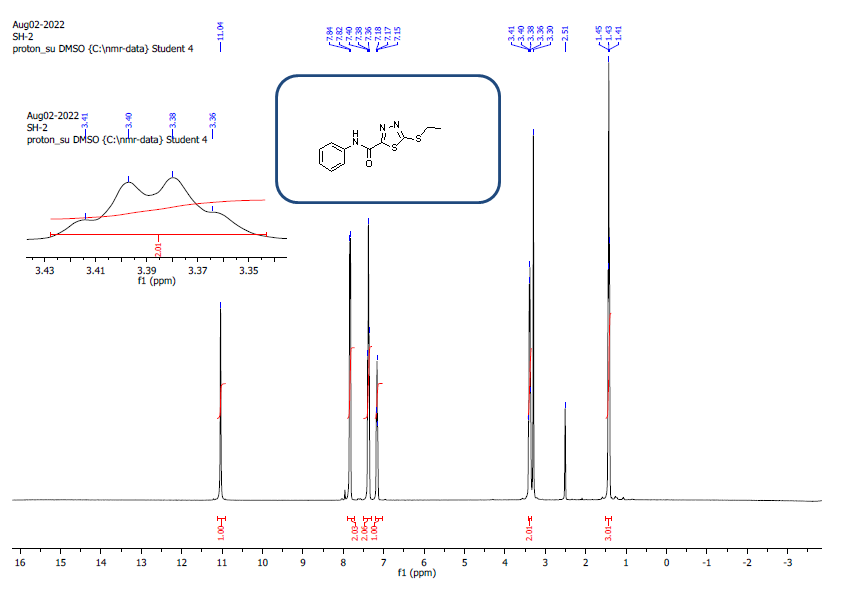
**

Figure S18

**
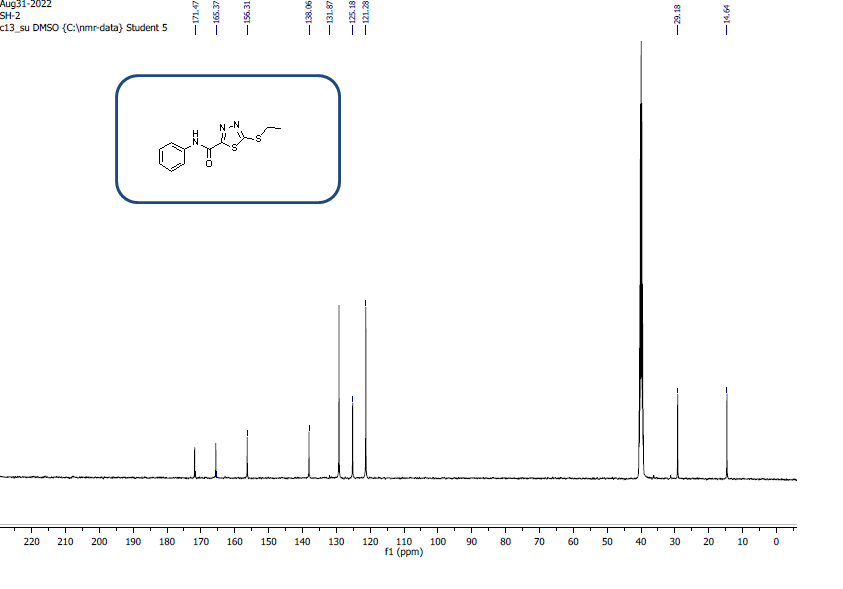
**

Figure S19

**
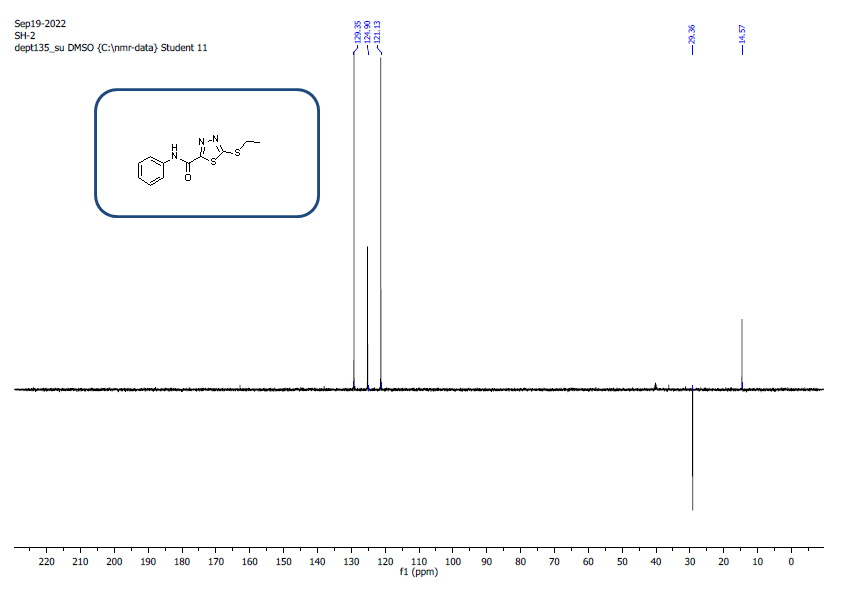
**

Figure S20

**5-((2-Methylbutyl)thio)-N-phenyl-1,3,4-thiadiazole-2-carboxamide (6a):**

White crystals, yield 84%, mp. 150-152 ºC; FT-IR (ATR) *δ*_max_: 3340 (NH_amidic_ str.), 3061 (CH_arom_ str.), 2961-2869 (CH_aliphatic_ str.), 1667 (C=O_amidic_ str.), 1599 (C=N str.); ^1^H NMR: *δ* 11.04 (s, H, NH), 7.82-7.10 (m, 5H, ArH), 3.33, 3.31 (d, 2H ,SCH_2_), 1.62, 1.60, 1.59, 1.53, 1.50 (m, 3H, (CH(CH_3_)CH_2_)), 0.87, 0.86, 0.82 ppm (t, 6H, CH_3_)CH_2_CH_3_); ^13^C NMR: *δ* 171.90 (C=O), 165.44, 156.25 (2C, Thiadiazole), 138.06, 129.15, 125.11, 121.21 (Arom.), 44.50, 37.88, 32.79, 27.40, 22.40 ppm. Anal. Calcd. for C_14_H_17_N_3_OS_2_ (307.43): C, 54.69; H, 5.57; N, 13.67; S, 20.86% Found: C, 54.46; H, 5.77; N, 13.58; S, 20.73%.


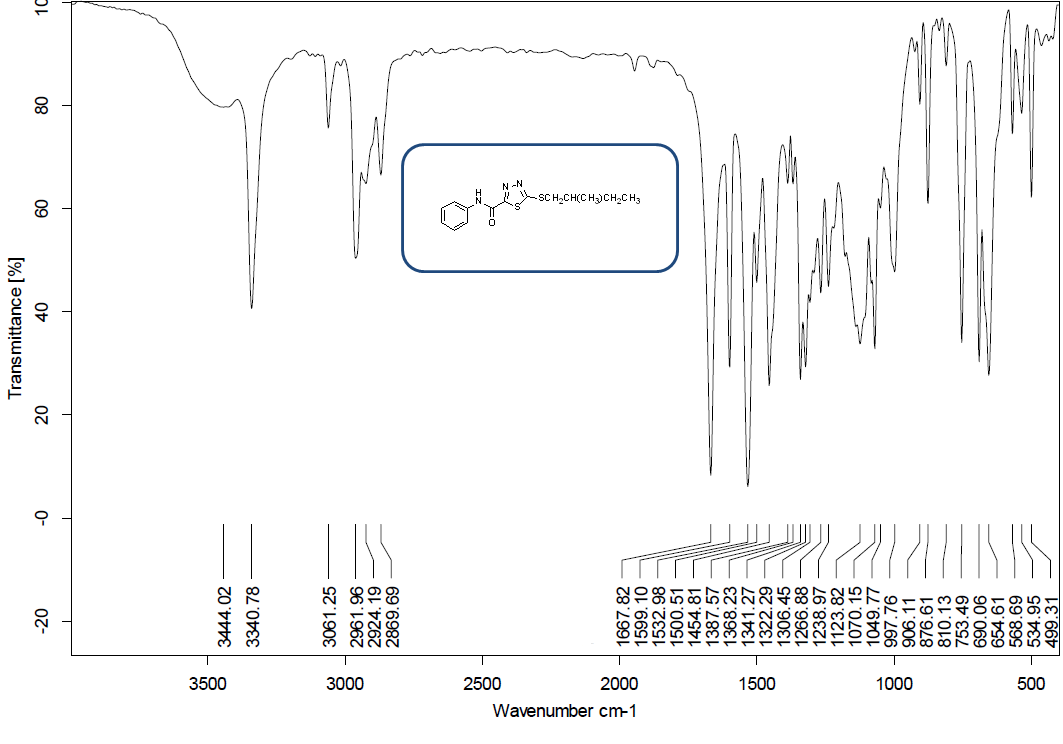


Figure S 21
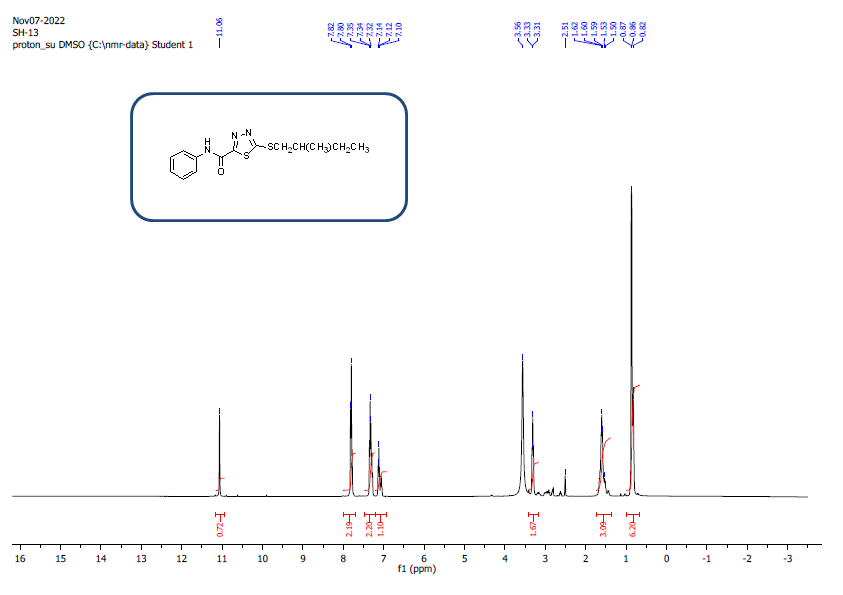


Figure S 22
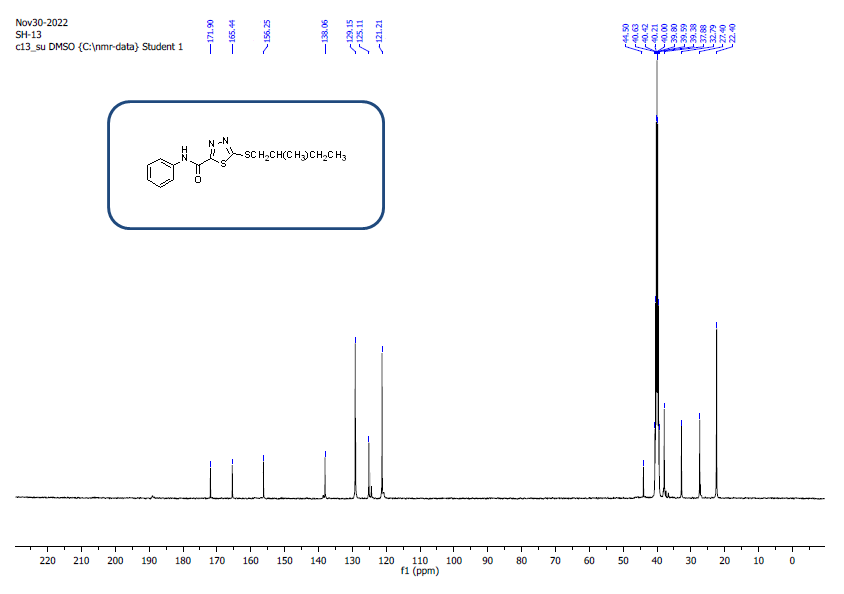
 Figure S23

**5-(Benzylthio)-N-phenyl-1,3,4-thiadiazole-2-carboxamide (7a):**

White crystals, yield 95%, mp. 237-239 ºC; FT-IR (ATR) *δ*_max_: 3223 (NH str.), 3056 (CH_arom_ str.), 2958 (CH_2alip_ str.), 1667 (C=O_amidic_ str.), 1625 (C=N str.); ^1^H NMR *δ* 11.05 (s, H, NH), 7.83-7.15(m, 10H, ArH), 4.67(s, 2H, SCH_2_) ppm; ^13^C NMR *δ* 171.19 (C=O), 165.87,156.26 (2C, Thiadiazole), 138.03, 136.40, 129.66, 129.25, 129.11, 128.32, 125.23, 121.25 (Arom.), 38.109 ppm (CH_2_). Dept-135 NMR; 129.61, 129.25, 129.11, 128.32, 125.28, 121.31 (Arom.), 38.20 ppm (CH_2_). Anal. Calcd. for C_16_H_13_N_3_OS_2_ (327.42): C, 58.69; H, 4.00; N, 12.83; S, 19.59% Found: C, 58.66; H, 4.28; N, 12.19; S, 19.89%.

**
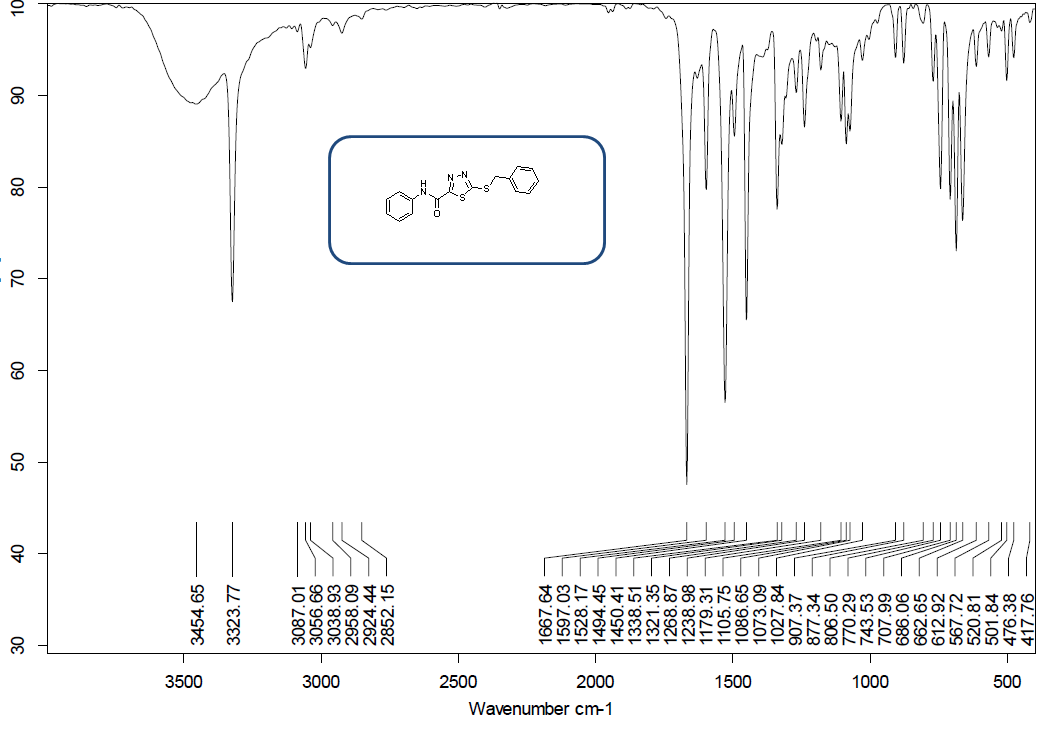
**

Figure S24

**
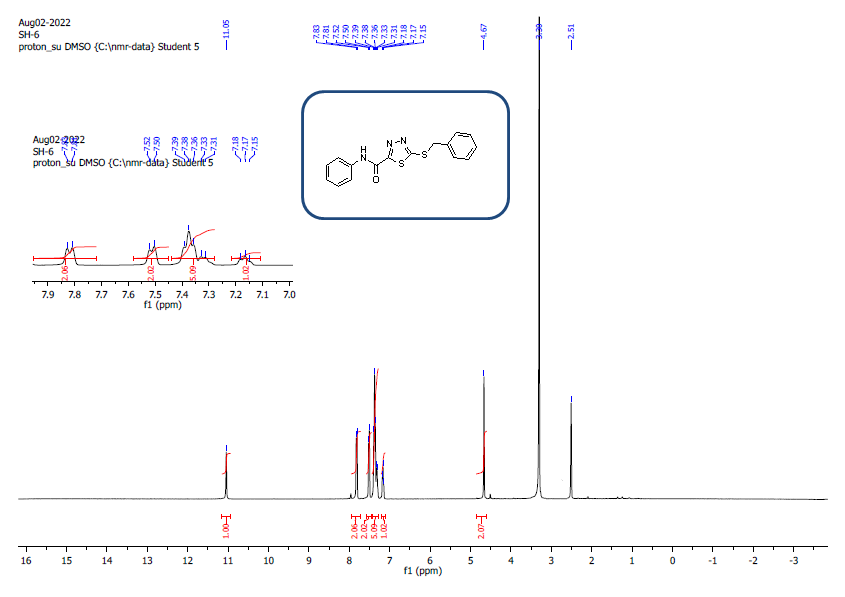
**

Figure S **25**

**
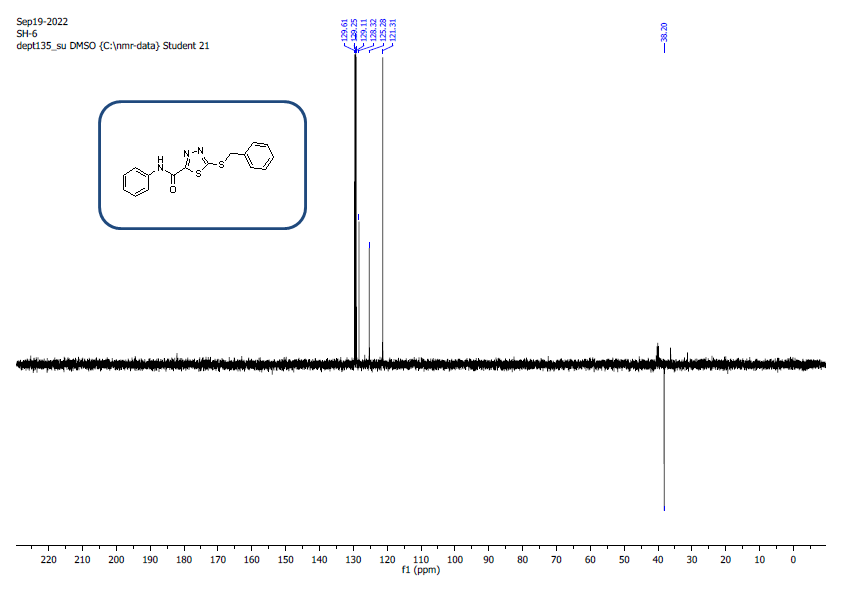
**

Figure S **26**

**
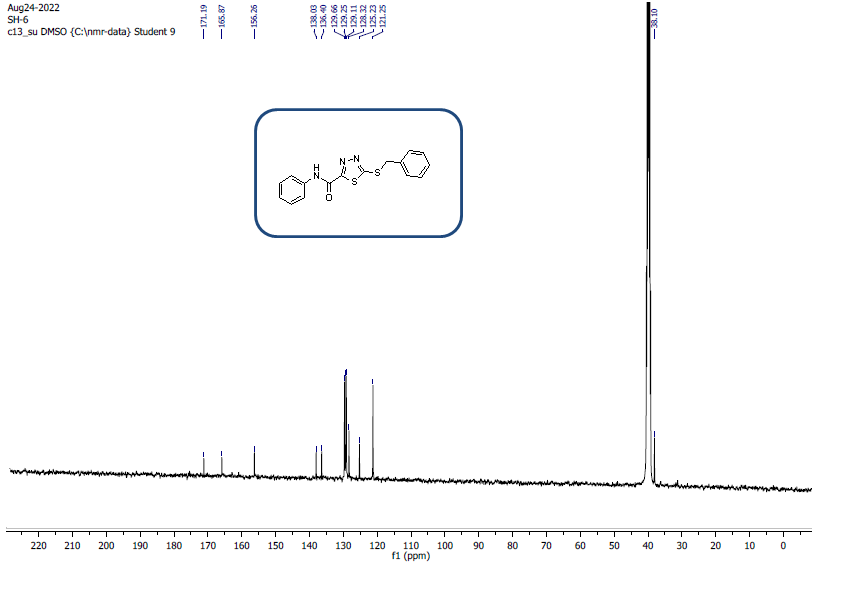
**

Figure S27

**5-(Benzylthio)-N-(o-tolyl)-1,3,4-thiadiazole-2-carboxamide (7b)***:*

White crystals, yield 78%, mp. 211-213 ºC; FT-IR (ATR) *δ*_max_: 3345 (NH str.), 3105 (CH_arom_ str.), 2971 (CH_3_ str.), 1662 (C=O str.), 1605 (C=N str.); ^1^H NMR: *δ* 11.05 (s, H, NH), 7.83-7.15 (m, 9H, ArH ), 4.67 (s, 2H, SCH_2_), 1.21 ppm (s, 3H, CH_3_); ^13^C NMR: *δ* 171.19 (C=O), 165.87, 156.26 (2C, Thiadiazole), 140.98, 136.40, 133.74, 129.25, 129.11, 128.32, 125.23, 121.25 (Arom.), 38.10 (CH_2_), 23.09 ppm (CH_3_). Anal. Calcd. for C_17_H_15_N_3_OS_2_ (341.45): C, 59.80; H, 4.43; N, 12.31; S, 18.78% Found: C, 59.87; H, 4.33; N, 12.37; S, 18.54%.

**
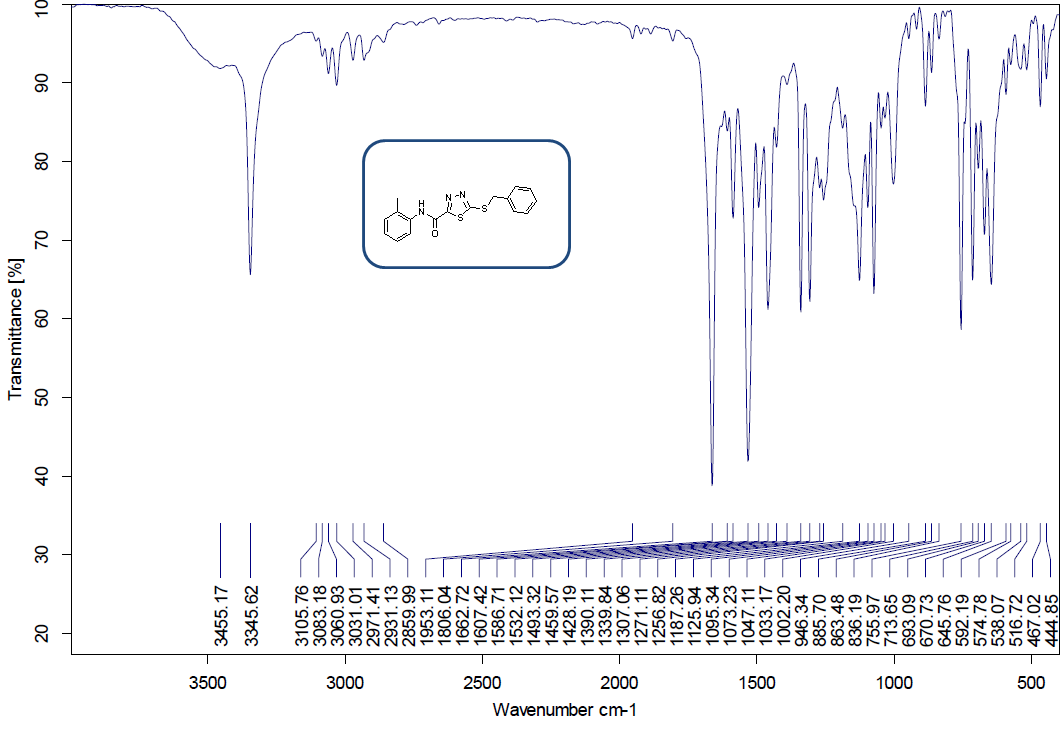
**

Figure S 28


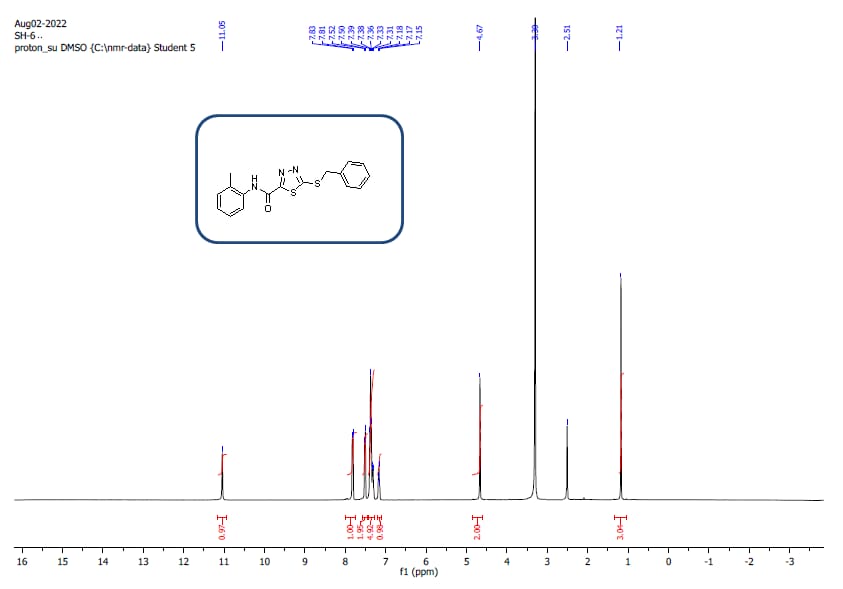


Figure S 29


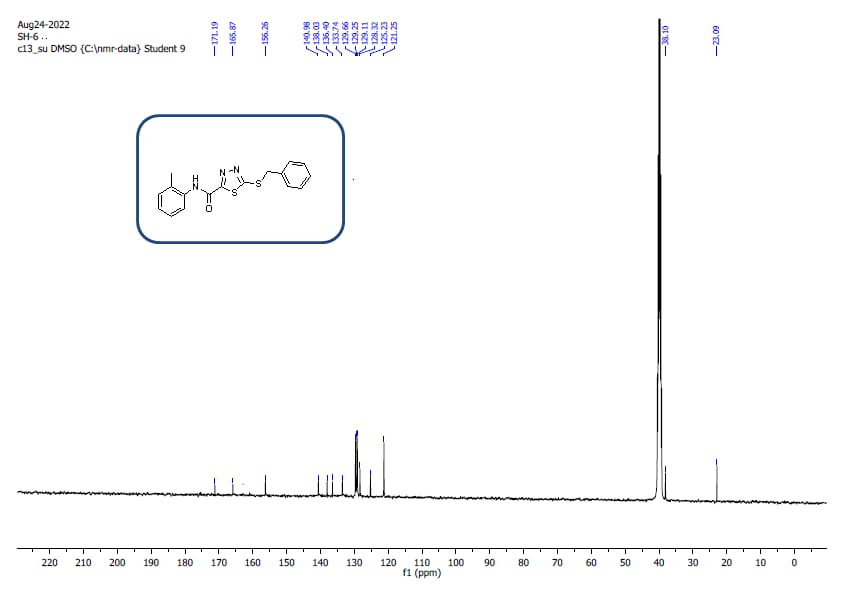


Figure S 30

**5-((2-Oxopropyl)thio)-N-phenyl-1,3,4-thiadiazole-2-carboxamide (8a):**

White crystals, yield 93.5%, mp. 217-219 ºC; FT-IR (ATR) *δ*_max_: 3332 (NH str.), 3060 (CH_arom_ str.), 2917 (CH_3 alip_ str.), 1710 (C=O str.) 1661 (C=O_amidic_ str.), 1622 (C=N str.); ^1^H NMR: *δ* 11.03 (s, H, NH), 7.83-7.17 (m, 5H, ArH), 4.52 (s, 2H, SCH_2_), 2.31 ppm (s, 3H, CH_3_); ^13^C NMR: *δ* 201.67 (C=O), 170.85 (C=O_amidic_), 165.93, 156.31 (2C, Thiadiazole), 137.94, 129.25, 125.29, 121.06, 44.64 (CH_2_), 29.18 ppm (CH_3_), Dept-135; 129.34, 125.19, 121.39 (Arom.), 44.59 (SCH_2_), 29.02 ppm (CH_3_). Anal. Calcd. for C_12_H_11_N3O_2_S_2_ (293.36): C, 49.13; H, 3.78; N, 14.32; S, 21.86% Found: C, 49.53; H, 3.68; N, 14.39; S, 21.69%.

**
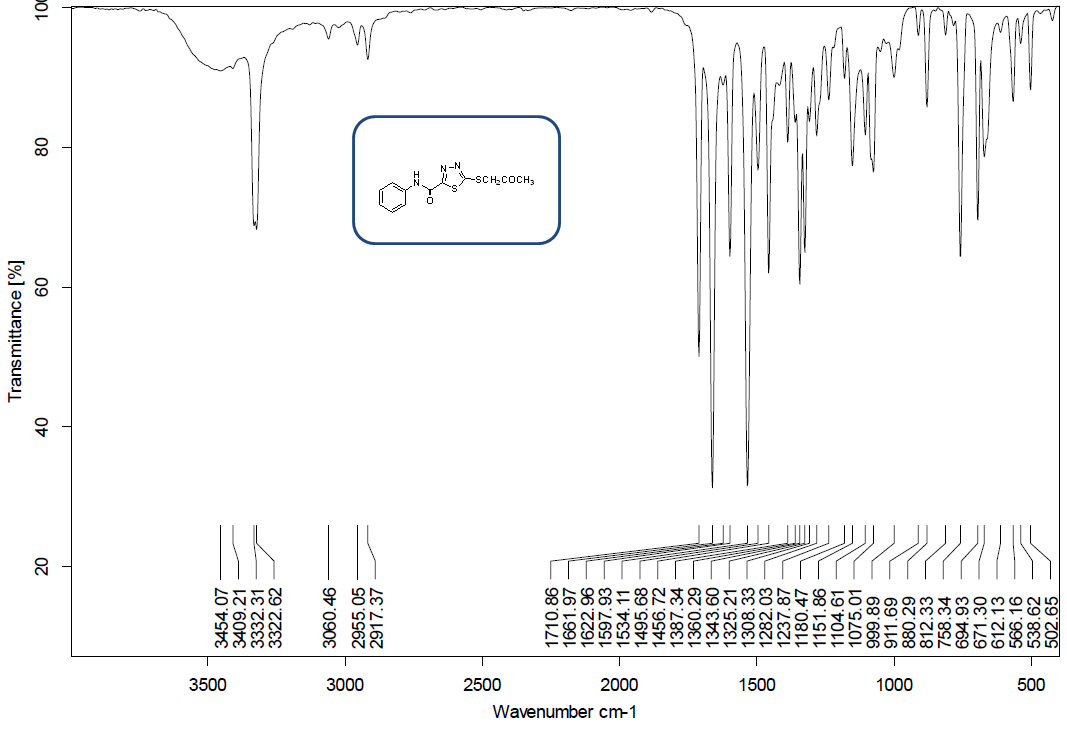
**

Figure S 31

**
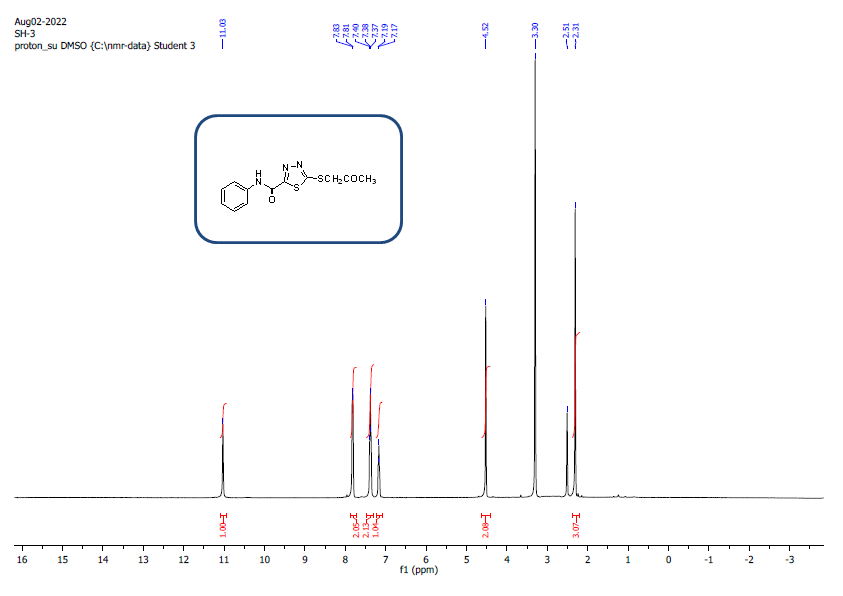
**

Figure S **32**

**
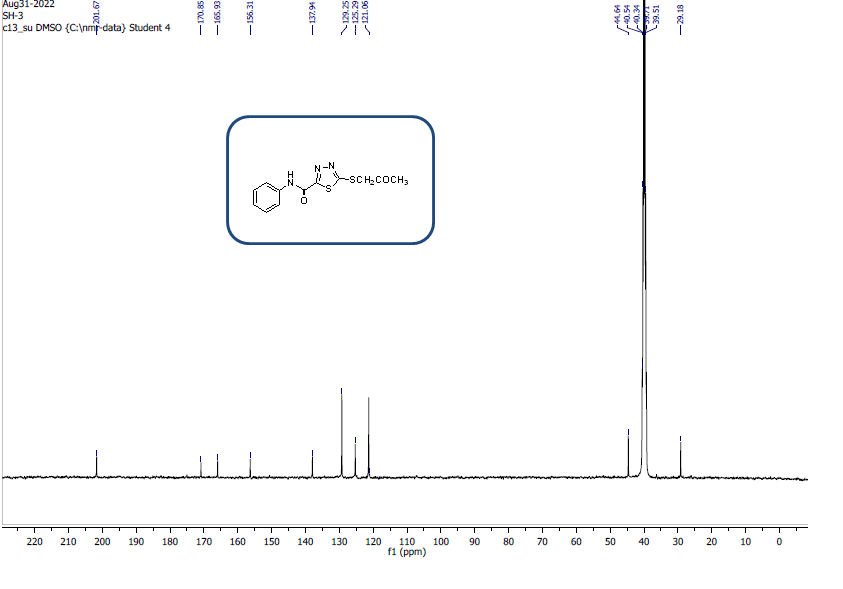
**

Figure S 33

**
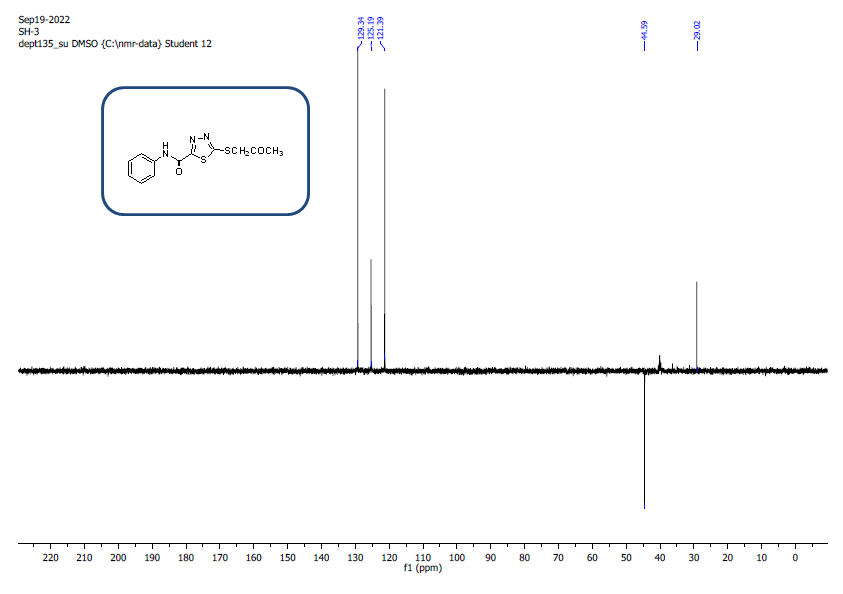
**

Figure S 34

**5-((2-Oxopropyl)thio)-N-(o-tolyl)-1,3,4-thiadiazole-2-carboxamide (8b):**

White powder, yield 81.5%, mp. 205-207 ºC; FT-IR (ATR) *δ*_max_: 3325 (NH str.), 3060 (CH_arom_ str.), 2957(CH_3alip_ str.), 1713 (C=O str.), 1667 (C=O_amidic_ str.), 1606 (C=N str.); ^1^H NMR: *δ* 10.61 (s, H, NH), 7.39-7.19 (m, 4H, ArH ), 4.52 (s, 2H, SCH_2_), 2.30 (s, 3H, COCH_3_), 2.24 (s, 3H, CH_3_ ArH) ppm; ^13^C NMR: *δ* 201.57 (C=O), 170.71 (C=O_amidic_), 165.69, 156.44, (2C, Thiadiazole), 135.85, 130.93, 127.11, 126.66, 126.64 (Arom.), 44.64 (SCH_2_), 28.93 (COCH_3_), 18.10 ppm (CH_3_ Arom**)**. Anal. Calcd. for C_13_H_13_N_3_O_2_S_2_ (307.39): C, 50.79; H, 4.26; N, 13.67; S, 20.86% Found: C, 50.33; H, 4.87; N, 13.47; S,20.71%.

**
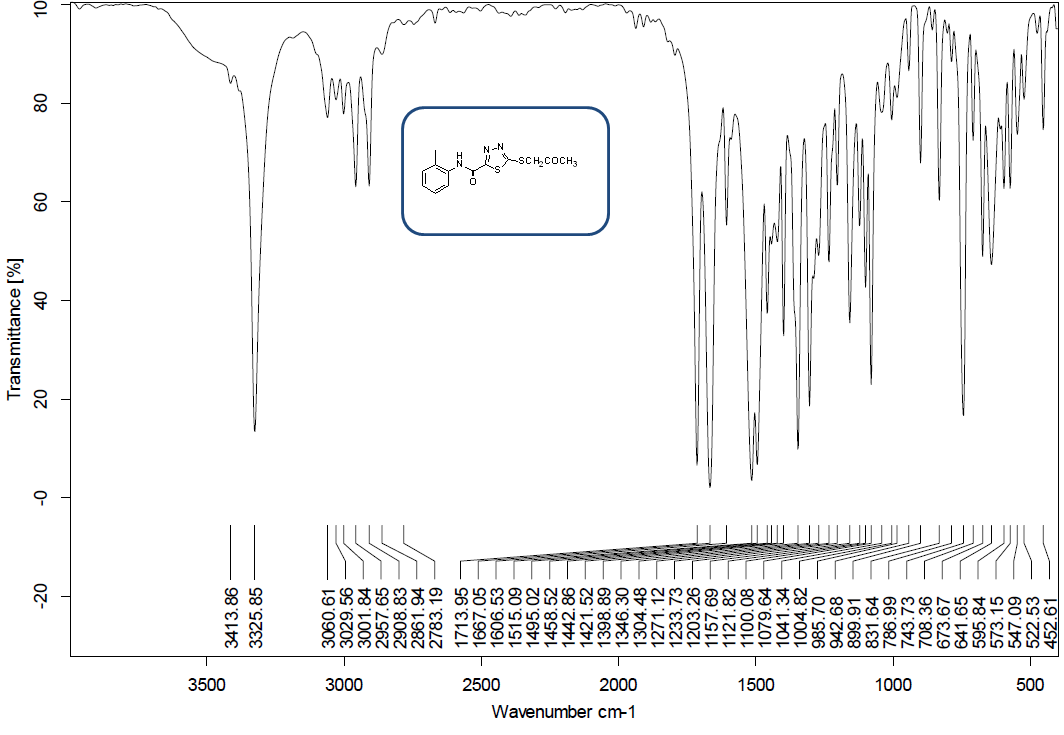
**

Figure S 35

**
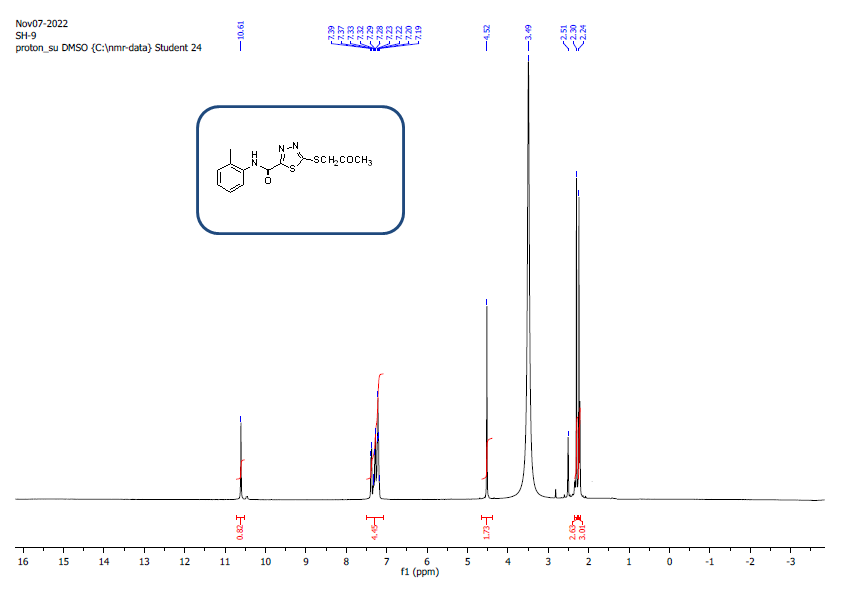
**

Figure S **36**

**
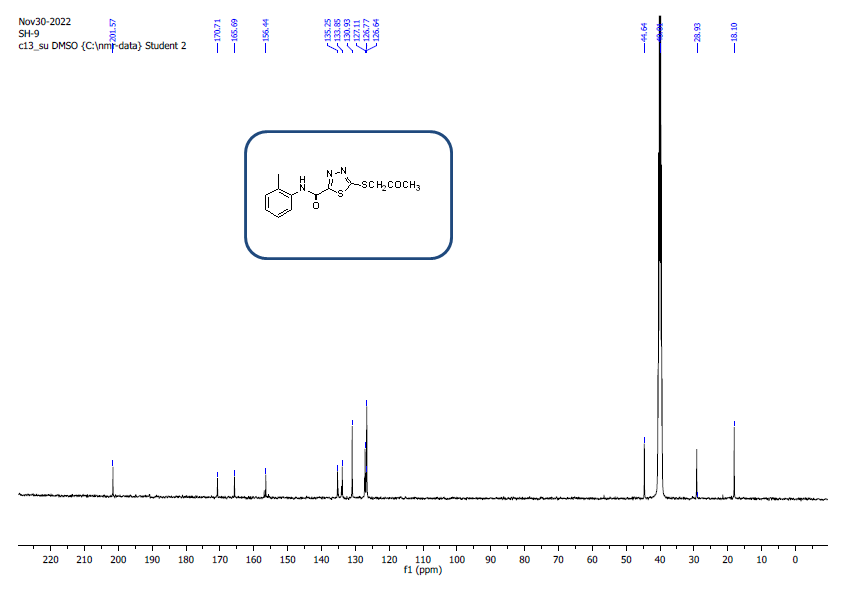
**

Figure S 37

***N*-(4-methoxyphenyl)-5-((2-oxopropyl)thio)-1,3,4-thiadiazole-2-carboxamide (8c):**

White crystals, yield 72%, mp. 267-269 ºC; FT-IR (ATR) *δ*_max_: 3343 (NH), 3067 (CH_arom_), 2949-2854 (CH_2_CH_3alip_), 1710 (C=O), 1661 (C=O_amidic_), 1622 (C=N str.); ^1^H NMR: *δ* 10.07 (s, H, NH), 7.83-7.38 (dd, 4H, ArH, *J* = 8.08Hz), 4.52 (s, 2H, SCH_2_), 4.09 (s,3H, COCH_3_)), 2.31 ppm (s, 3H, OCH_3_); ^13^C NMR:*δ* 201.67 (C=O), 170.85 (C=O_amidic_), 165.93, 156.31 (2C, Thiadiazole), 137.94, 129.25, 125.29, 121.31 (Arom.), 57.13 **(**OCH_3_**)**, 44.64 (SCH_2_), 29.35 ppm (COCH_3_). Anal. Calcd. for C_13_H_13_N_3_O_3_S_2_ (323.39): C, 48.28; H, 4.05; N, 12.99; S, 19.83% Found: C, 48.65; H, 4.35; N, 12.78; S, 19.48%.


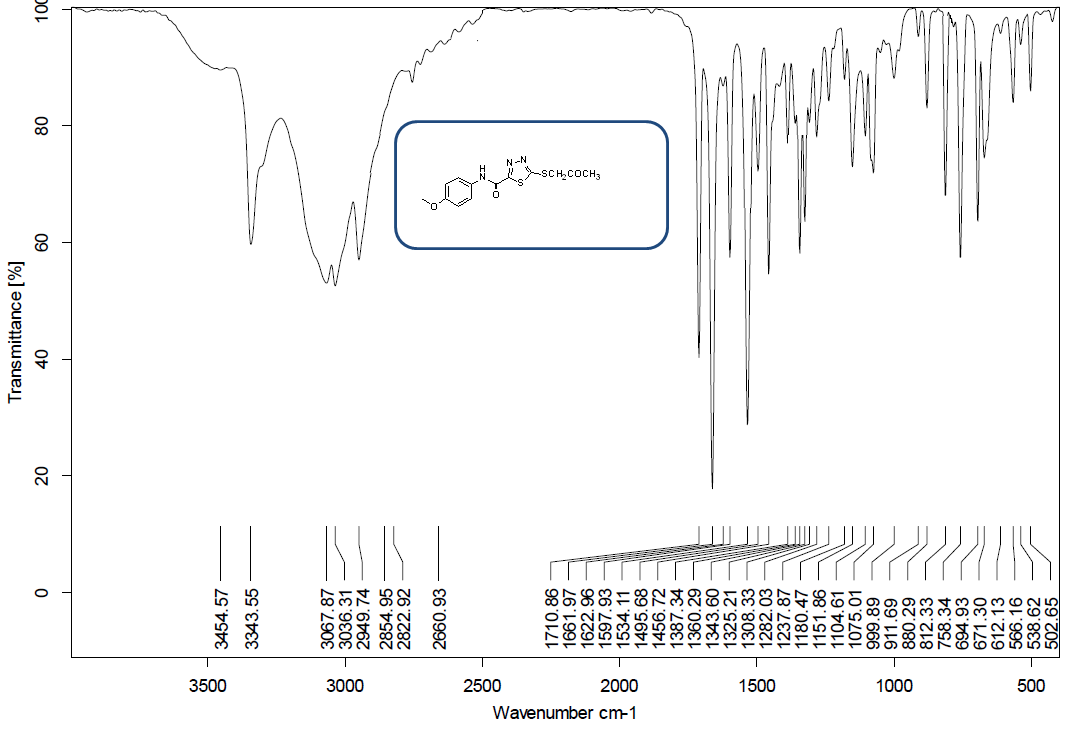


Figure S 38

**
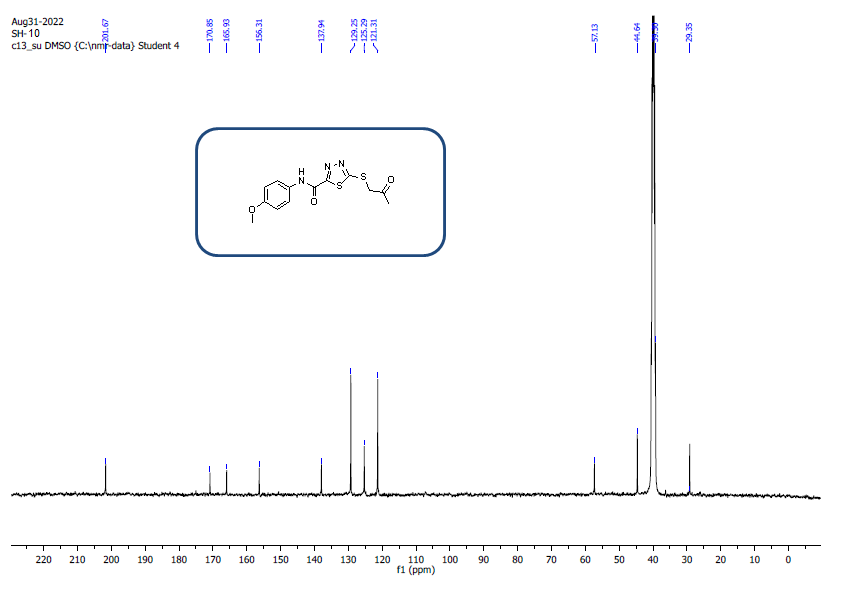
**

Figure S 39

**
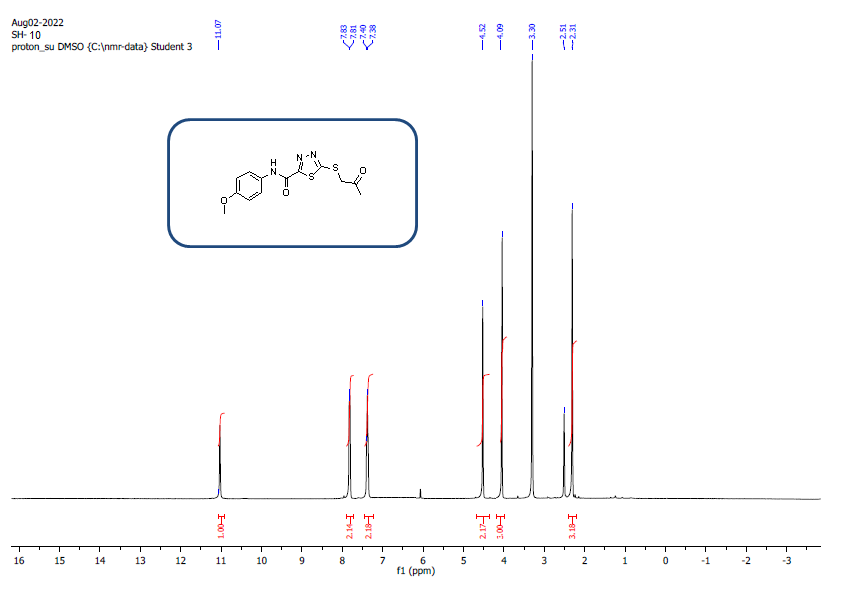
**

Figure S 40

***N*-(4-nitrophenyl)-5-((2-oxopropyl) thio)-1,3,4-thiadiazole-2-carboxamide (8d):** White crystals, yield 66%, mp. 280-282 ºC; FT-IR (ATR) *δ*_max_: 3345 (NH str.), 3191 (CH_arom_ str.), 3104-2921 (CH_2_, CH_3alip_ str.), 1762 (C=O str.), 1678 (C=O_amidic_ str.), 1659 (C=N str.), 1536,1341 (NO_2_ str.); ^1^H NMR: *δ* 11.15 (s, H, NH), 7.89-7.44 (dd, 4H, ArH, *J* = 8.08Hz), 4.67 (s, 2H, SCH_2_), 2.15 ppm (s, 3H, CH_3_),; ^13^C NMR:*δ* 202.02 (C=O), 171.89 (C=O_amidic_), 166.54, 157.13 (2C, Thiadiazole), 138.11, 129.66, 125.51, 121.82 (Arom.), 44.69 (SCH_2_), 29.67 ppm (COCH_3_). Anal. Calcd. for C_12_H_10_N_4_O_4_S_2_ (338.36): C, 42.60; H, 2.98; N, 16.56; S, 18.95% Found: C, 42.63; H, 2.95; N, 16.59; S, 18.94%.

**
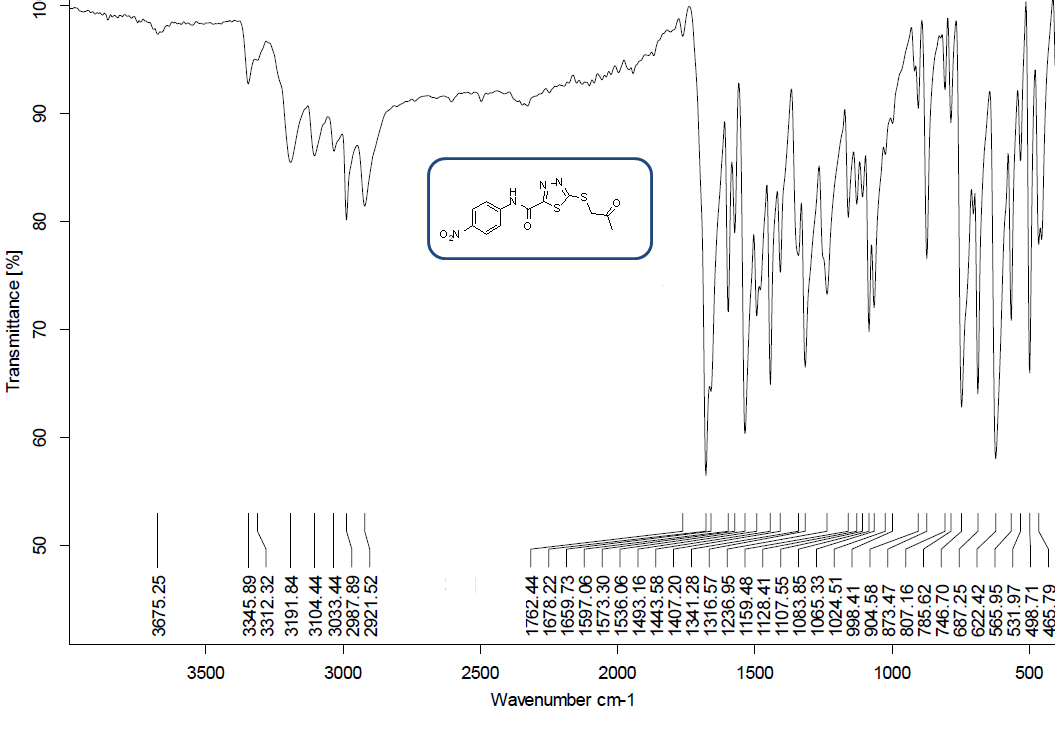
**

Figure S 41

**
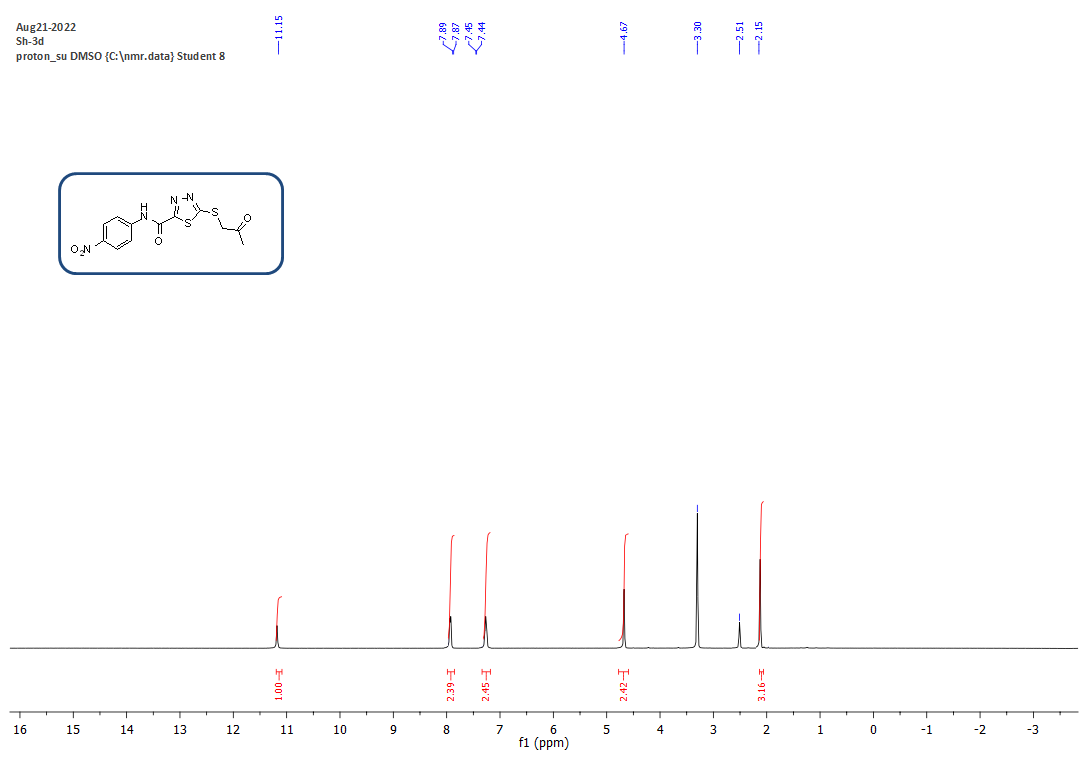
**

Figure S 42 **
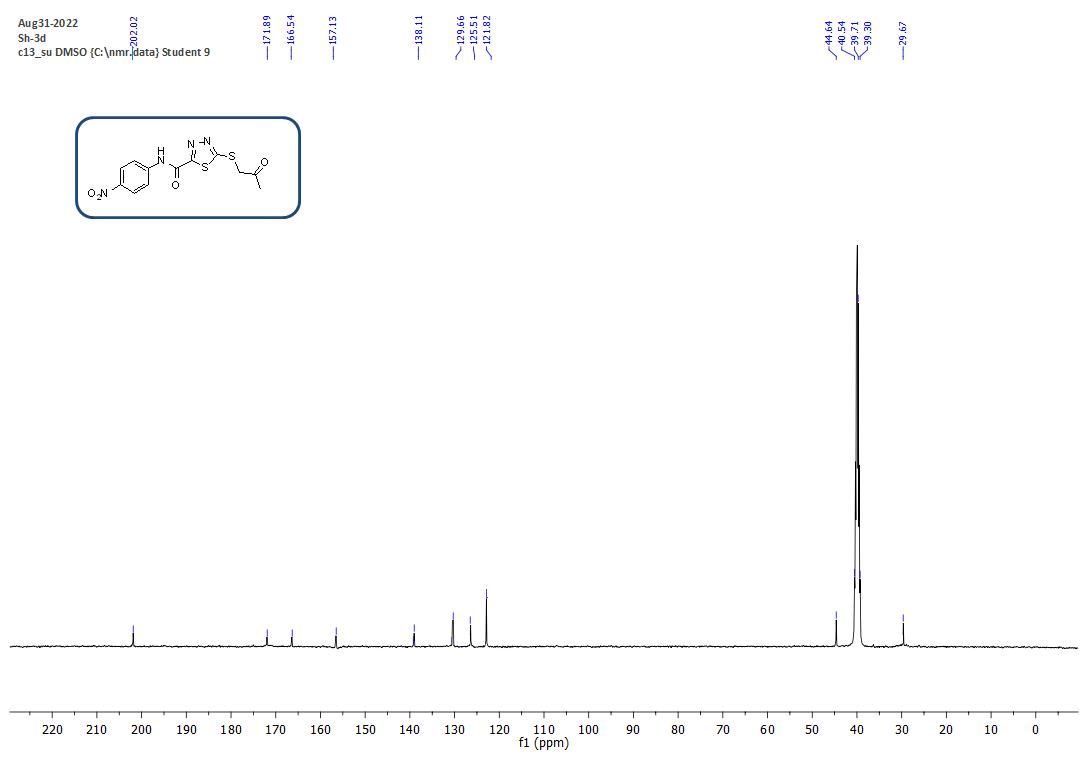
**

Figure S 43

**Ethyl 2-((5-(phenylcarbamoyl)-1,3,4-thiadiazol-2-yl)thio)acetate (9a):**

White crystals, yield 64%, mp. 133-13; FT-IR (ATR) *δ* _max_: 3537 (NH_amidic_ str.), 3142 (CH_arom_ str.), 2983-2905 (CH_2_, CH_3alip_ str.), 1737.94 (C=O str.), 1664.28 (C=O_amidic_ str.), 1605 (C=N str.); ^1^H NMR: *δ* 11.05 (s, H, NH), 7.83-7.15 (m, 5H, ArH ), 4.35 (s, 2H, SCH_2_), 4.20, 4.18, 4.16, 4.14 (q, 2H, CH_2_, *J* =7.08Hz), 1.23, 1.22, 1.20 ppm (s, 3H, CH_3_, *J* =7.08Hz); ^13^C NMR: *δ* 170.42 (C=O), 168.08, (C=O _amidic_), 166.38, 156.55 (2C, Thiadiazole), 137.87, 128.85, 125.61, 121.2 (Arom.), 3,62.19, 36.23, 14.34 ppm (CH_3_). Dept-135; 129.31, 125.21, 121.40 (Arom.), 62.12, (SCH_2_), 35.67 (COOCH_2_), 14.56 ppm (CH_3_). Anal. Calcd. for C_13_H_13_N_3_O_3_S_2_ (323.39): C, 48.28; H, 4.05; N, 12.99; S, 19.83% Found: C, 48.50; H, 4.17; N, 12.58; S, 19.48%.

**
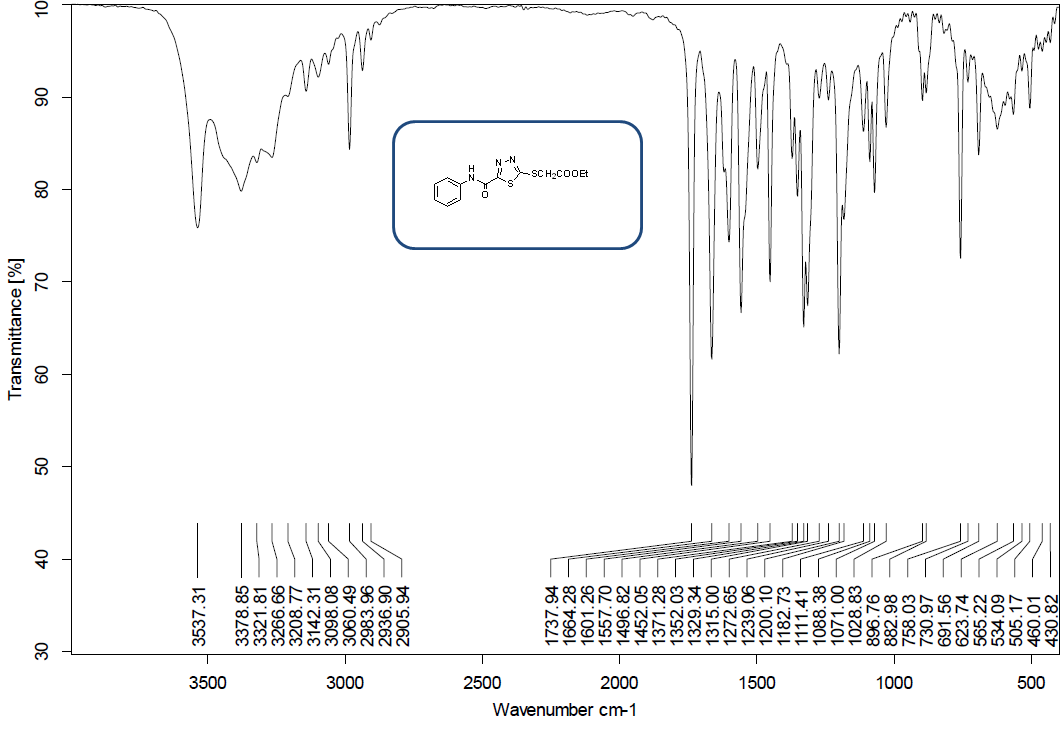
**

Figure S 44

**
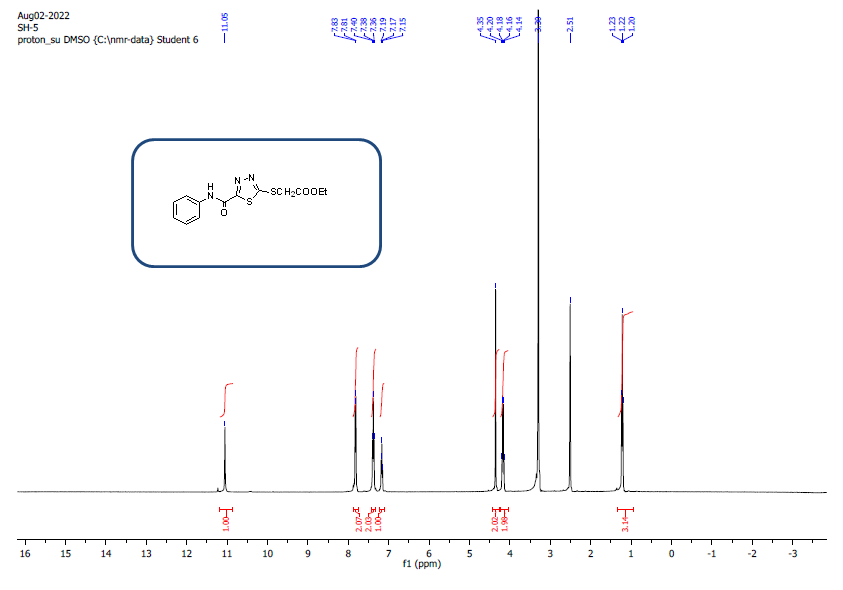
**

Figure S 45

**
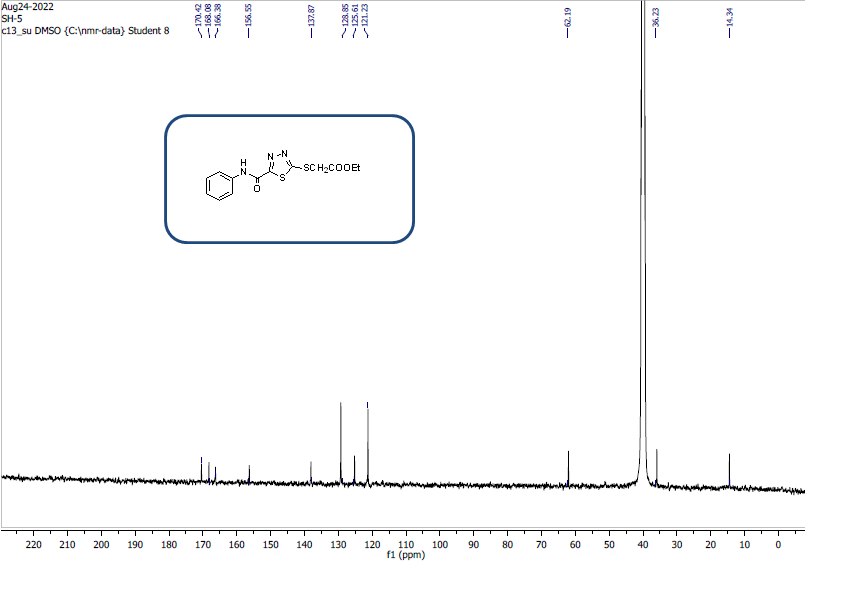
**

Figure S 46

**
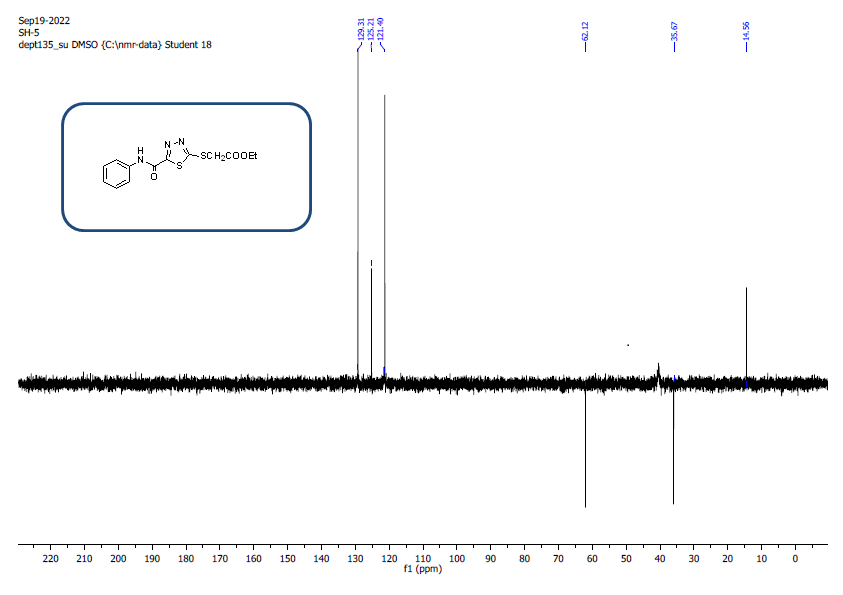
**

Figure S 47

**2-((5-(Phenylcarbamoyl)-1,3,4-thiadiazol-2-yl)thio)acetic acid (10a):**

White crystals, yield 90%, mp. 199-201 ºC; FT-IR (ATR) *δ*_max_: 3322 (NH_amidic_ str.), 3104 (br OH str.), 3061 (CH_arom_ str.), 2979-2926 (CH_2_CH_3alip_ str.), 1721 (C=O str.) 1665 (C=O_amidic_ str.), 1599 (C=N str.); ^1^H NMR: *δ* 15.11 (s, H, COOH), 11.04 (s, H, NH), 7.81 -7.18, (m, 5H, ArH ), 4.52 (s, 2H, SCH_2_) ppm; ^13^C NMR: *δ* 170.63 (C=O), 169.39 (C=O_amidic_), 165.95, 156.33 (2C, Thiadiazole), 137.86, 129.28, 125.36, 121.35 (Arom.), 36.36 ppm (CH_2_). Dept-135; 129.32, 125.50, 121.40 (CH Arom), 36.27 ppm (CH_2_). Anal. Calcd. for C_11_H_9_N_3_O_3_S_2_ (295.34): C, 44.73; H, 3.07; N, 14.23; S, 21.71% Found: C, 44.33; H, 3.37; N, 14.43; S, 21.53%**.**

**
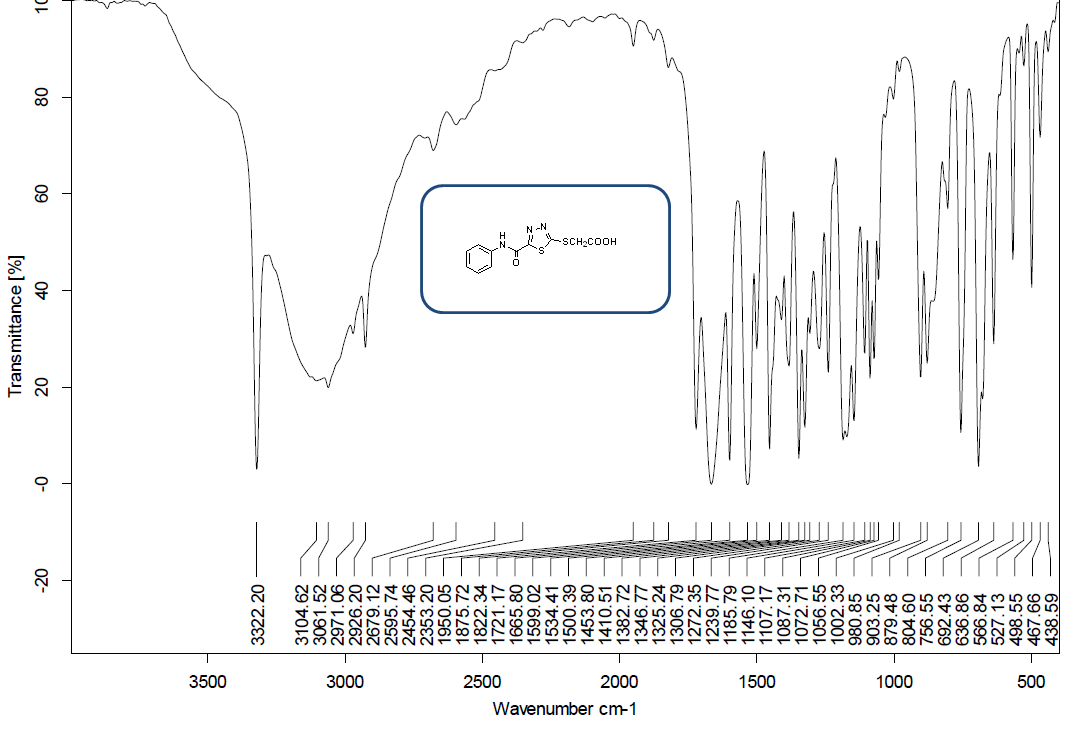
**

Figure S 48

**
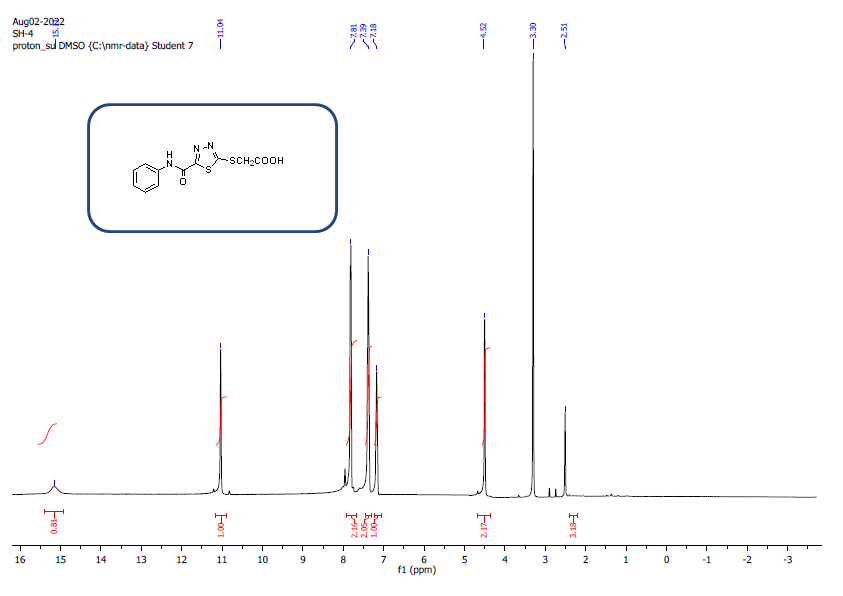
**

Figure S **49**

**
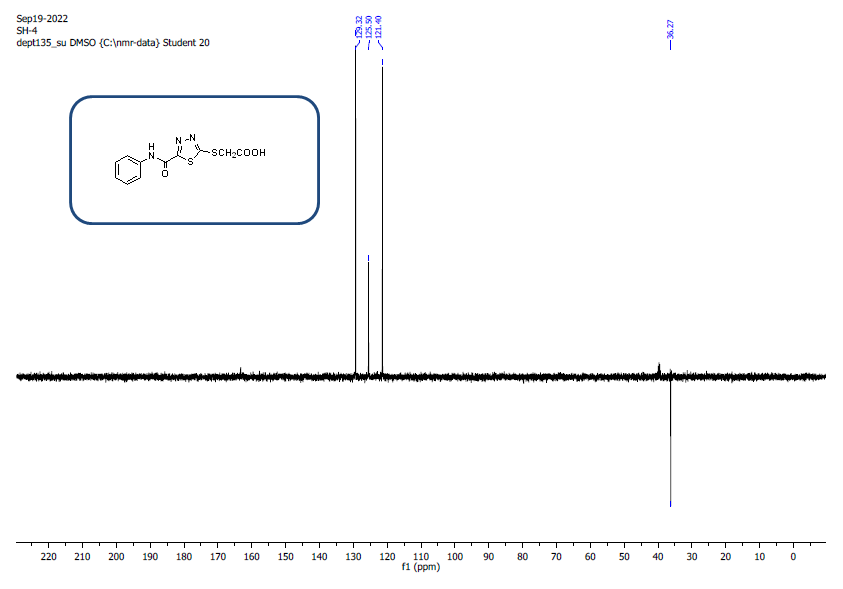
**

Figure S **50**

**
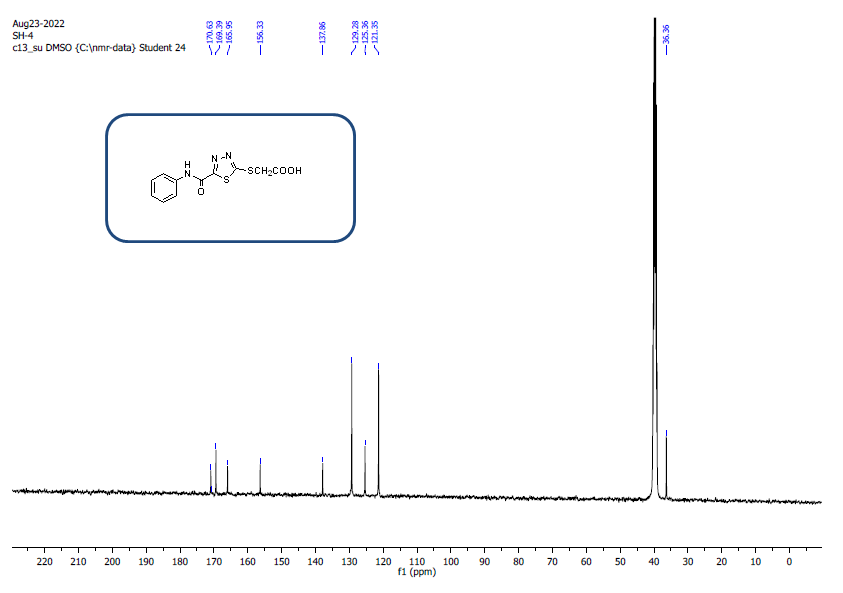
**

Figure S 51

**O-ethyl *S*-(5-(phenylcarbamoyl)-1,3,4-thiadiazol-2-yl) carbonothioate (11a):**

White powder, yield 53%, mp. 166-168 ºC; FT-IR (ATR) *δ*_max_: 3349 (NH_amidic_ str.), 3055 (CH_arom_ str.), 2984-2870 (CH_2_CH_3alip_ str.), 1729.32 (C=O str.) 1682 (C=O_amidic_ str.), 1641 (C=N str.); ^1^H NMR: *δ* 11.23 (s, H, NH), 7.82 -7.15 (m, 5H, ArH ), 4.76-4.67 (q, 2H, CH_2_, *J* =10.88Hz) , 1.05-1.00 ppm (t, 3H, CH_3_, *J* =10.88Hz); ^13^C NMR: *δ* 168.75 (C=O), 164.29 (C=O), 158.51, 155.86 (2C,Thiadiazole), 137.87, 129.20, 125.20, 121.35 (Arom.), 56.53 (CH_2_), 18.86 ppm (CH_3_). Anal. Calcd. for C_12_H_11_N_3_O_3_S_2_ (309.36): C, 46.59; H, 3.58; N, 13.58; S, 20.73% Found: C, 46.44; H, 3.81; N, 13.78; S, 20.55%.


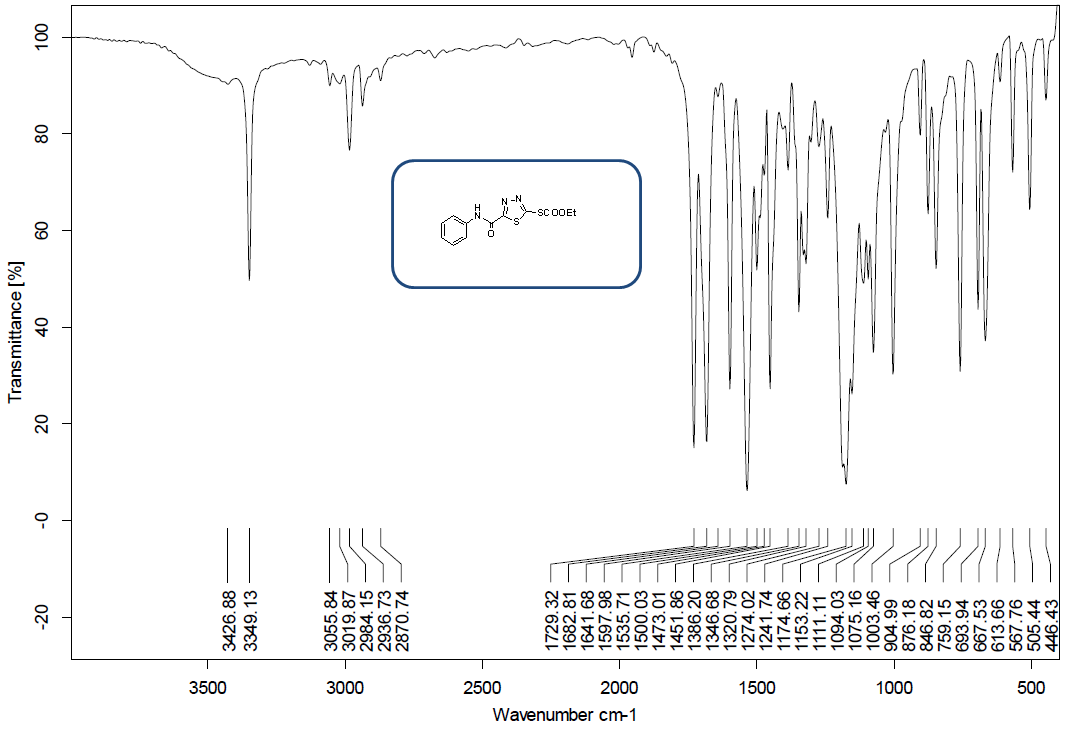


Figure S 52


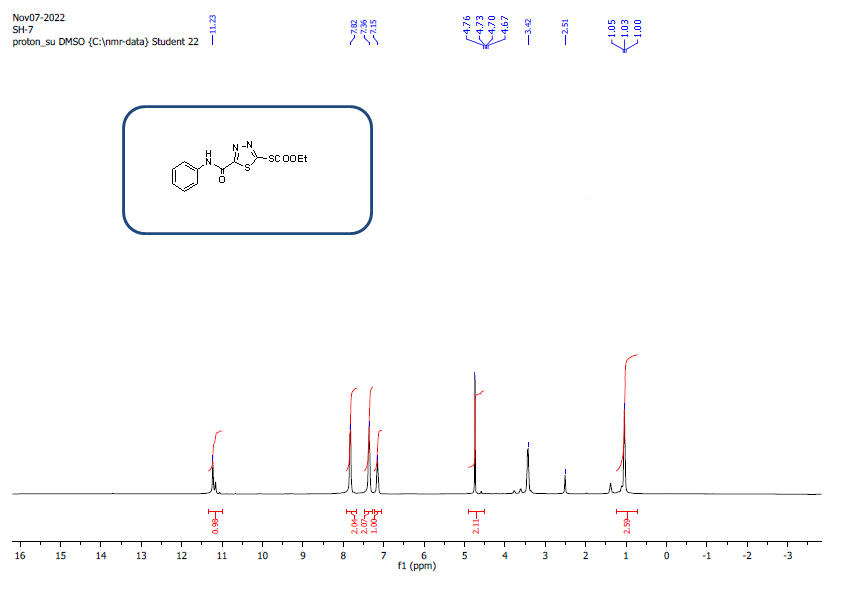


Figure S 53


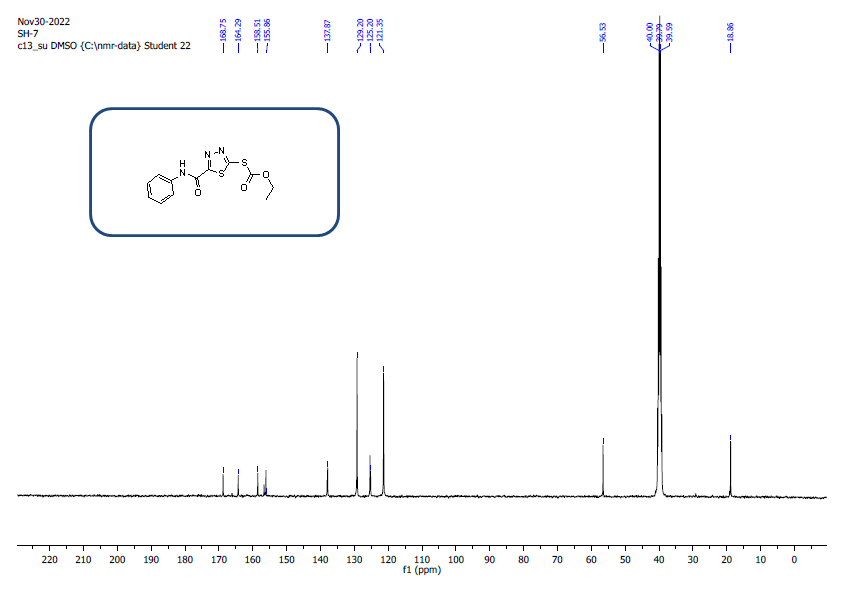


Figure S 54

***N*-phenyl-5-styryl-1,3,4-thiadiazole-2-carboxamide (12a)*:***

Yellow crystals, yield 71%, mp. 226-228 ºC FT-IR (ATR) *λ* max: 3328 ( NH str.), 3108 (CH_arom_ str.), 3057-2923 (CH=CH_alip_ str.), 1685 (C=O str.), 1599 (C=N str.); ^1^H NMR: *δ* 10.70 (s, H, NH, exchangeable by D_2_O), 7.85- 7.08 (m, 10H, ArH), 6.68 (s, 2H, CH=CH) ppm; ^13^C NMR: *δ* 167.81 (C=O), 163.20, 158.13 (2C, Thiadiazole), 144.36, 141.98, 139.27, 139.04, 138.66, 133.38, 129.05, 124.05, 120.74, 112.13 (Arom.) ppm . Anal. Calcd. for C_17_H_13_N_3_OS (307.37): C, 66.43; H, 4.26; N, 13.67; S, 10.43% Found: C, 66.38; H, 4.31; N, 13.61; S, 10.45%.


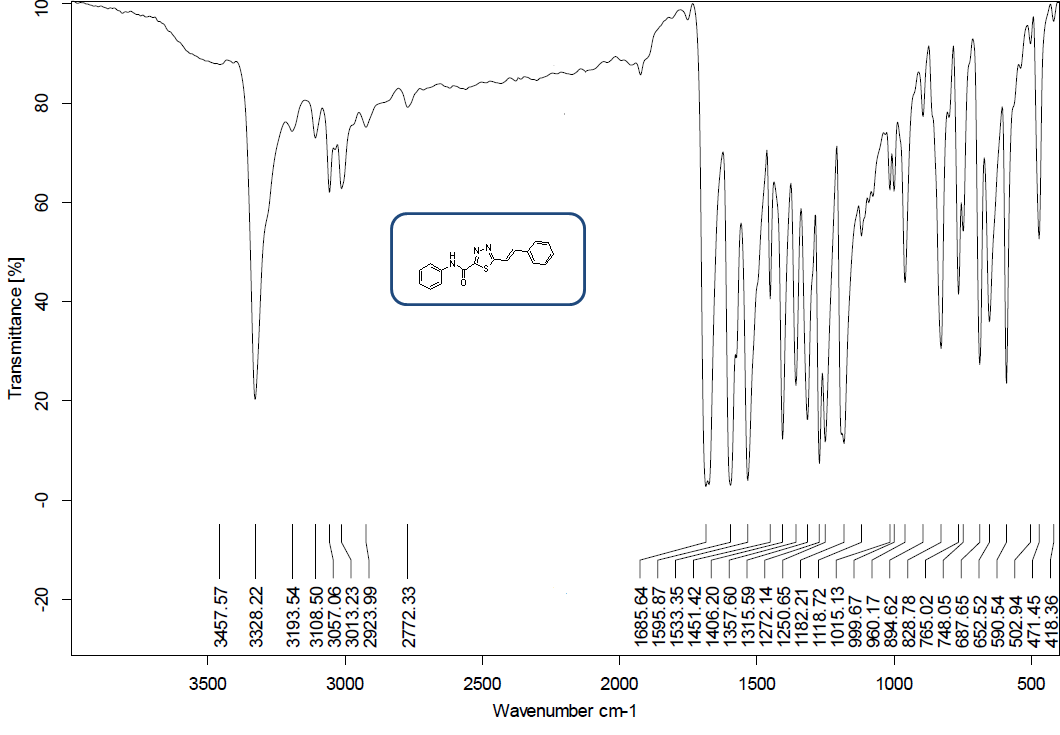


Figure S 55


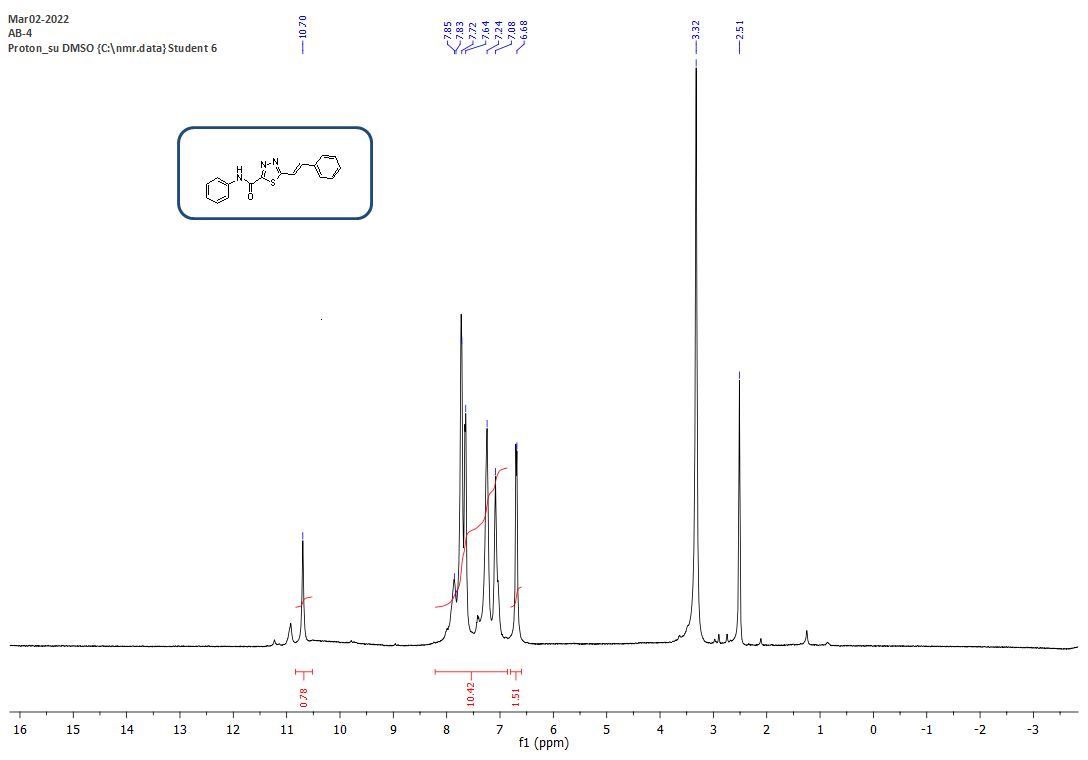


Figure S 56


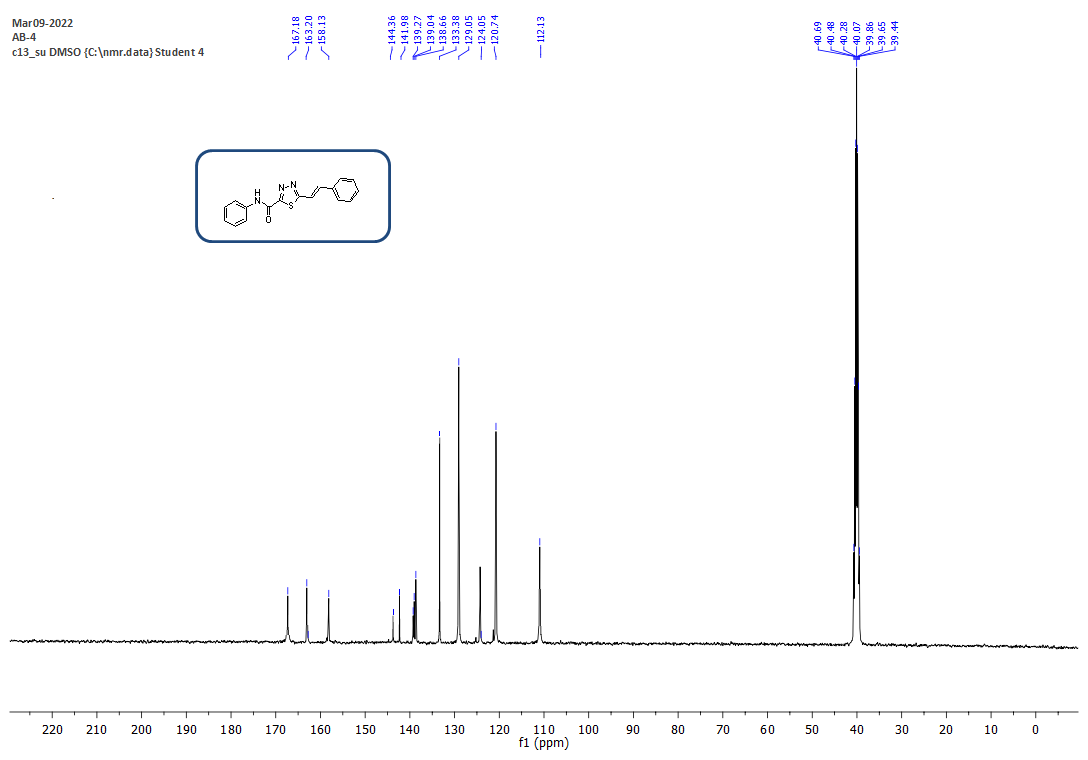


Figure S 57

**5-[4-(Dimethylamino)phenyl]-*N*-phenyl-1,3,4-thiadiazole-2-carboxamide (13a):**

Red crystals, yield 91%, mp. 210-212 ºC FT-IR (ATR) *λ* max: 3327 (NH str.), 3087 (CH_arom_ str.), 2983-2874 (CH_3_ str.), 1664 (C=O str.), 1625 (C=N str.); ^1^H NMR: *δ* 11.31 (s, H, NH_amidic_), exchangeable by D_2_O), 8.23 - 7.09 (m, 9H, ArH), 2.84 (s, 6H, 2CH_3_); ^13^C NMR: *δ* 171.33 (C=O), 166.60, 162.53 (2C, Thiadiazole), 156.24, 137.00, 135.08, 133.21, 129.07, 125.90, 125.34, 121.20 (Arom.), 36.42 ppm (2CH_3_). Anal. Calcd. for C_17_H_16_N_4_OS (324.40): C, 62.94; H, 4.97; N, 17.27; S, 9.88% Found: C, 62.85; H, 4.98; N, 17.29; S, 9.89%.


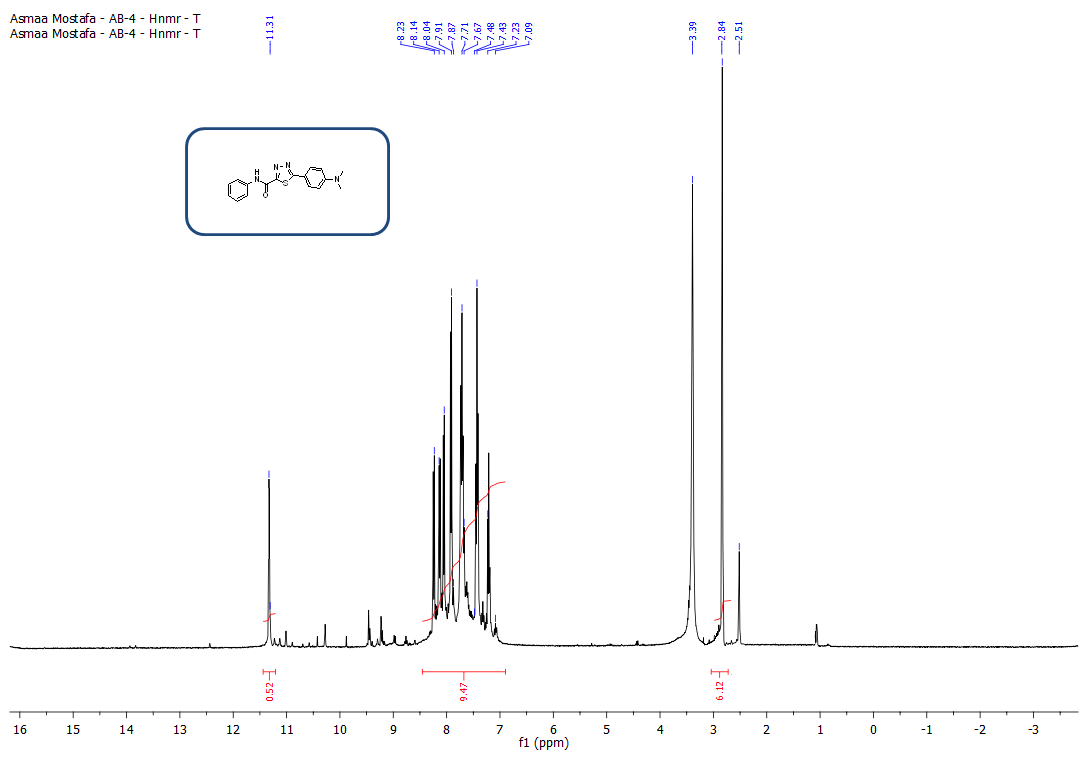


Figure S 58


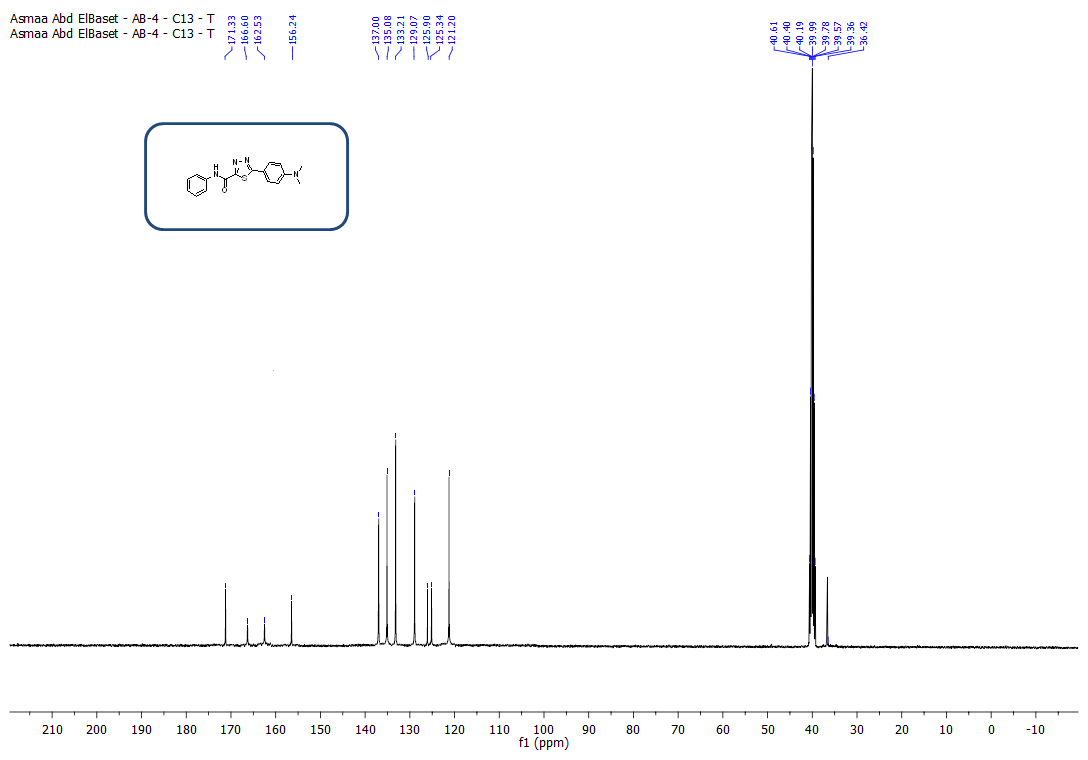


Figure S 59

***N*-Phenyl-5-(3,4,5-trimethoxyphenyl)-1,3,4-thiadiazole-2-carboxamide (14a):**

White crystals, yield 76%, mp. 199-201 ºC FT-IR (ATR) *λ* max: 3278 (NH str.), 3146 (CH_arom_ str.), 2998-2874 (OCH_3_ str.), 1671 (C=O str.), 1625 (C=N str.); ^1^H NMR: *δ* 10.21 (s, H, NH, exchangeable by D_2_O), 7.99- 7.12 (m, 7H, ArH), 3.94 (s, 9H, 3OCH_3_) ppm; ^13^C NMR: *δ* 173.54 (C=O), 167.53, 162.81 (2C, Thiadiazole), 151.80, 147.37, 135.70, 130.09, 126.28, 124.31, 122.13, 120.55 (Arom.), 56.56, 48.97 (3OCH_3_) ppm. Anal. Calcd. for C1_8_H_17_N_3_O_4_S (371.41): C, 58.21; H, 4.61; N, 11.31; S, 8.63% Found: C, 58.25; H, 4.62; N, 11.28; S, 8.58%.


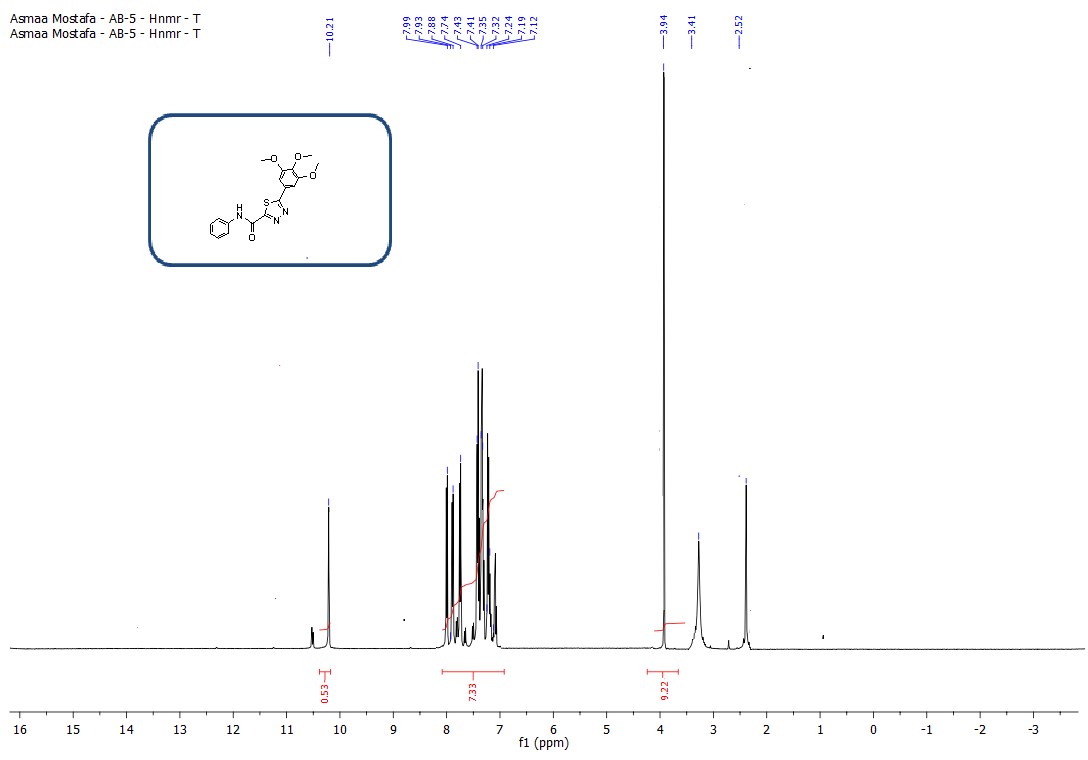


Figure S 60


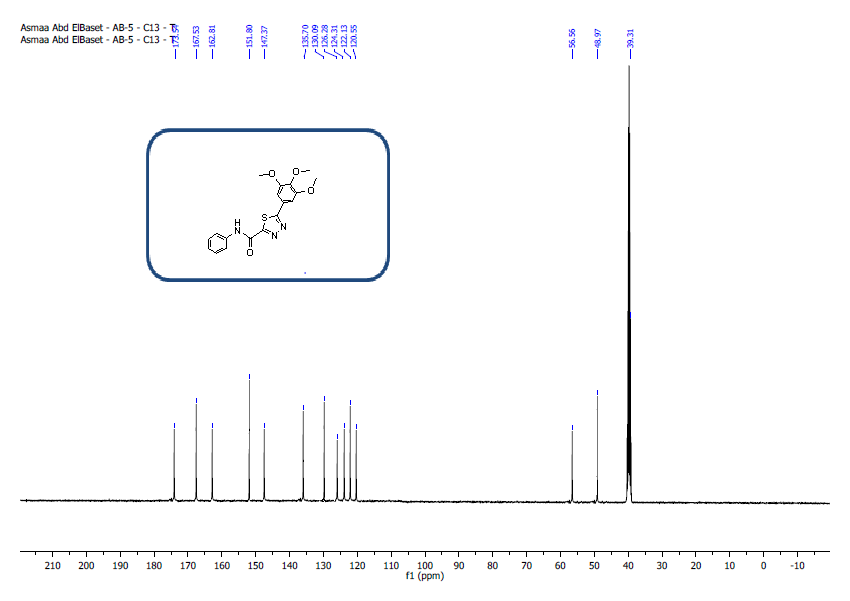


Figure S 61

**5-(Naphthalen-1-yl)-*N*-phenyl-1,3,4-thiadiazole-2-carboxamide (15a):**

Pale yellow crystals, yield 67%, mp. 245-247 ºC FT-IR (ATR) *λ* max: 3317 (NH str.), 3037-2985 (CH_arom_ str.), 1673 (C=O str.), 1625 (C=N str.); ^1^H NMR: *δ* 10.52 (s, H, NH_amidic_), exchangeable by D_2_O), 7.81- 7.09 ppm (m, 12H, ArH) ppm; ^13^C NMR: *δ* 171.96 (C=O), 164.76, 156.81 (2C, Thiadiazole), 135.95, 135.38, 133.68, 132.99, 129.43, 129.21, 129.25, 128.36, 128.21, 128.05, 121.29, 120.61, 119.76, 117.78 (Arom.) ppm. Anal. Calcd. For C_19_H_13_N_3_OS (331.39): C, 68.91; H, 3.93; N, 12.64; S, 9.68% Found: C, 68.86; H, 3.95; N, 12.68; S, 9.66%.


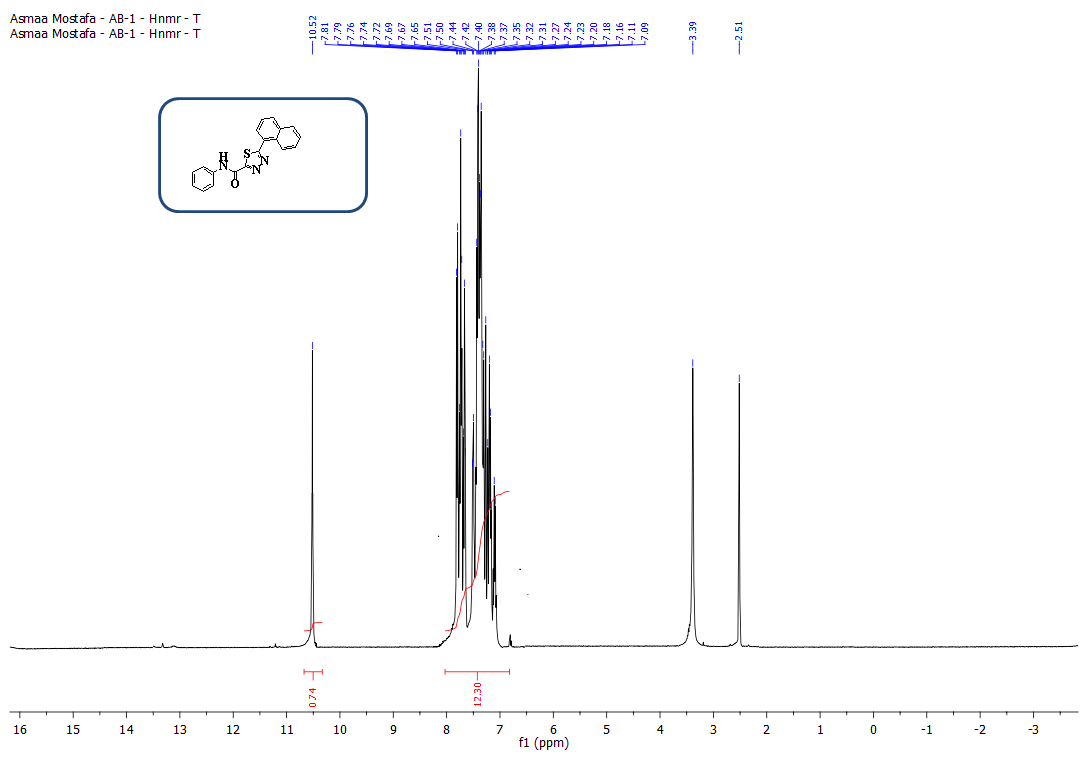


Figure S 62


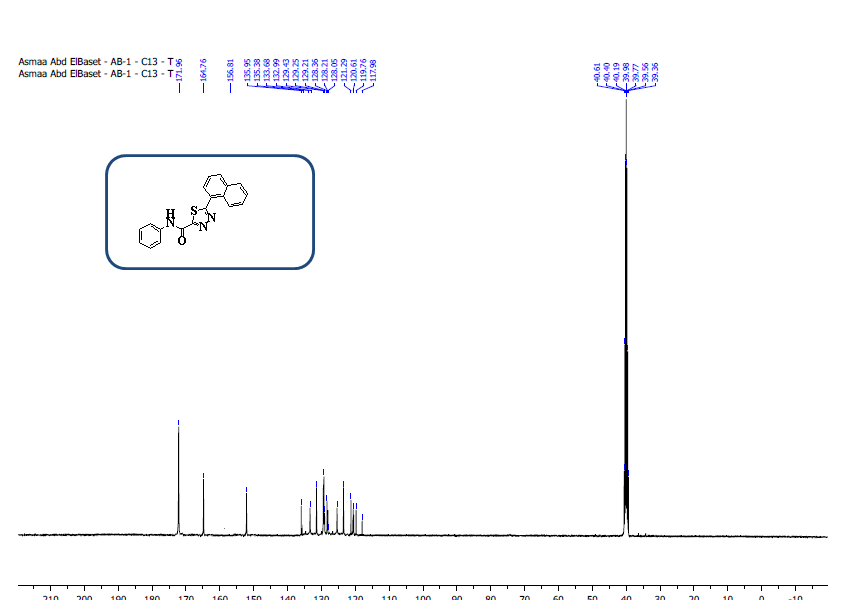


Figure S 63

**5-(Benzo[d][1,3]dioxol-5-yl)-*N*-phenyl-1,3,4-thiadiazole-2-carboxamide (16a):**

White crystals, yield 74%, 225-227 ºC FT-IR (ATR) *λ* max: 3330 (NH str.), 3059 (CH_arom_ str.), 2962 (CH_2alip_ str.), 1690 (C=O str.), 1622 (C=N str.); ^1^H NMR: *δ* 10.20 (s, H, NH, exchangeable by D_2_O), 7.75- 6.93 (m, 8H, ArH), 6.58 (s, 2H, CH_2_ piprenal) ppm; ^13^C NMR: *δ* 171.05 (C=O), 161.88, 158.45 (2C, Thiadiazole), 147.37, 146.15, 141.08, 139.21, 129.71, 127.85, 125.36, 121.57, 133.05, 111.04 (Arom.), 101.04 ( CH_2_ piprenal) ppm. Anal. Calcd. for C_16_H_11_N_3_O_3_S (325.34): C, 59.07; H, 3.41; N, 12.92; S, 9.86% Found: C, 59.20; H, 3.38; N, 12.81; S, 9.84%.


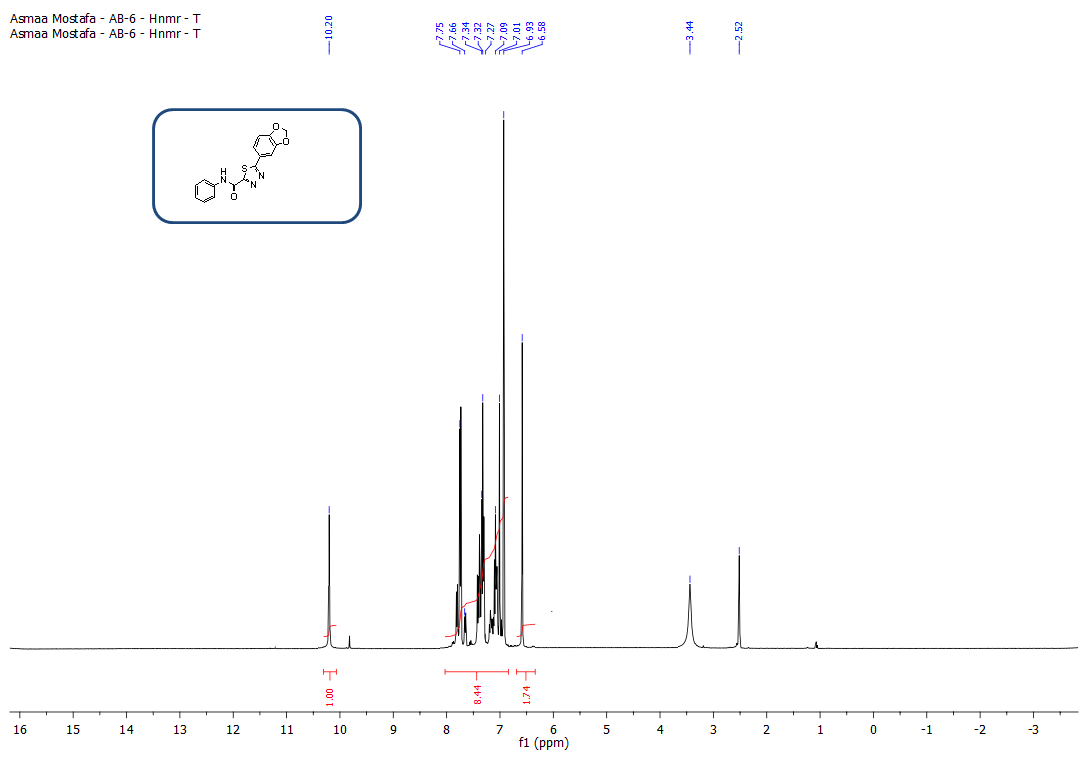


Figure S 64


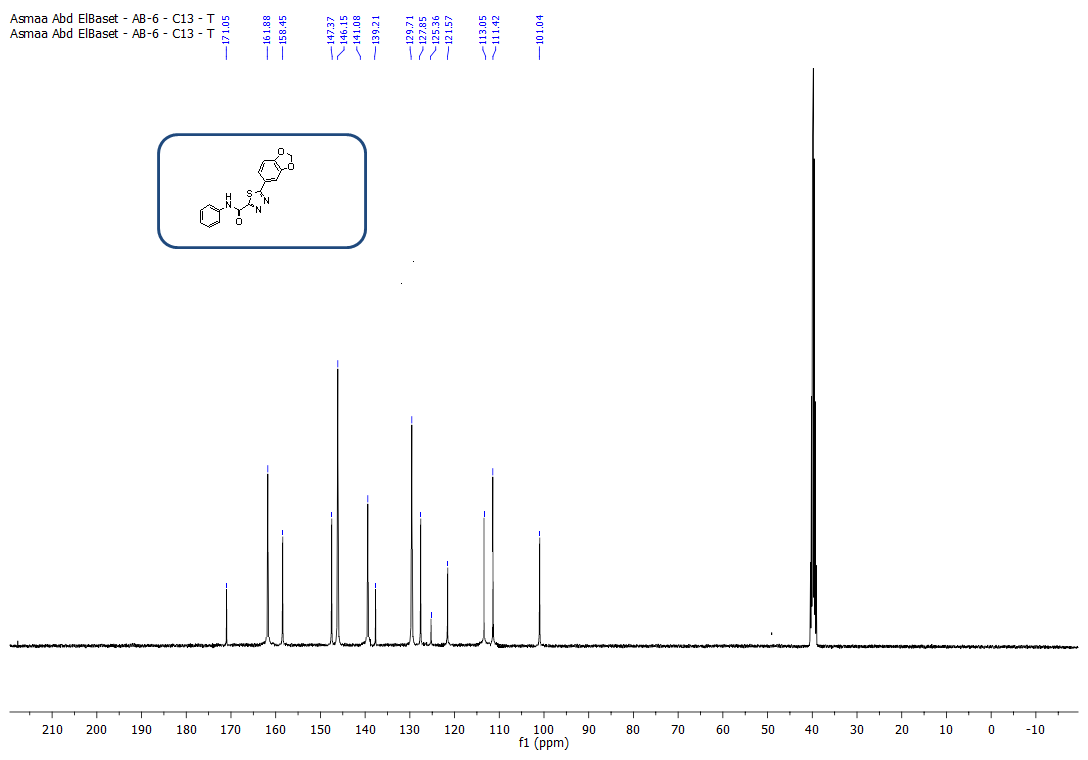


Figure S 65

***N-*phenyl*-5-(p-*tolyl)*-1*,3,4-thiadiazole*-*2-carboxamide (17a)*:***

Pale yellow, yield 77%, mp. 233-235ºC FT-IR (ATR) *λ* max: 3322 (NH str.), 3125 (CH_arom_ str.), 2976 (CH_3alip_ str.), 1677 (C=O str.), 1625 (C=N str.); ^1^H NMR: *δ* 10.20 (s, H, NH, exchangeable by D_2_O), 7.89- 7.13 (m, 9H, ArH), 3.41 (s, 3H, CH_3_) ppm; ^13^C NMR: *δ* 172.26 (C=O), 166.02, 156.66 (2C, Thiadiazole), 153.95, 141.17, 138.14, 129.24, 125.22, 124.85, 121.40, 105.40 (Arom.), 19.02 ppm (CH_3_). Anal. Calcd. For C_16_H_13_N_3_OS (295.36): C, 65.06; H, 4.44; N, 14.23; S, 10.86% Found: C, 64.98; H, 4.46; N, 14.21; S, 10.88%.


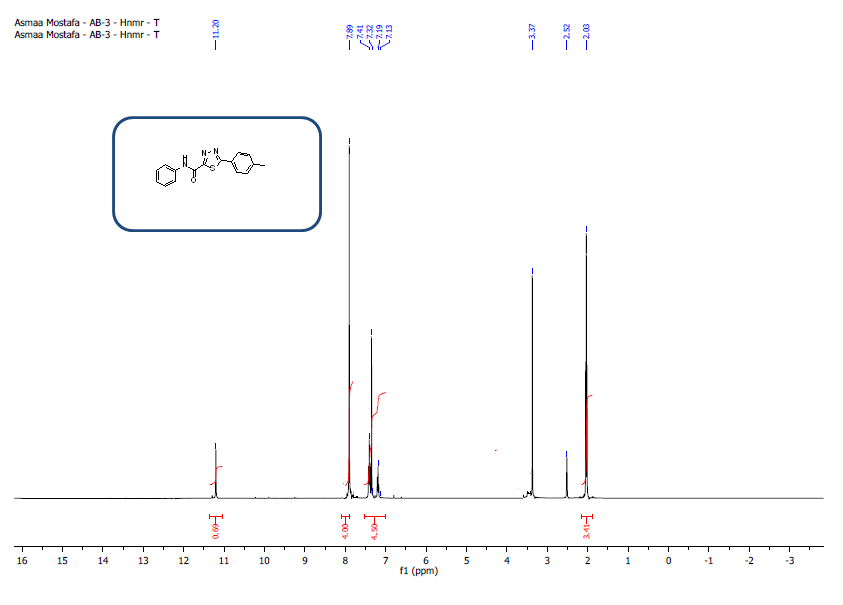


Figure S 66


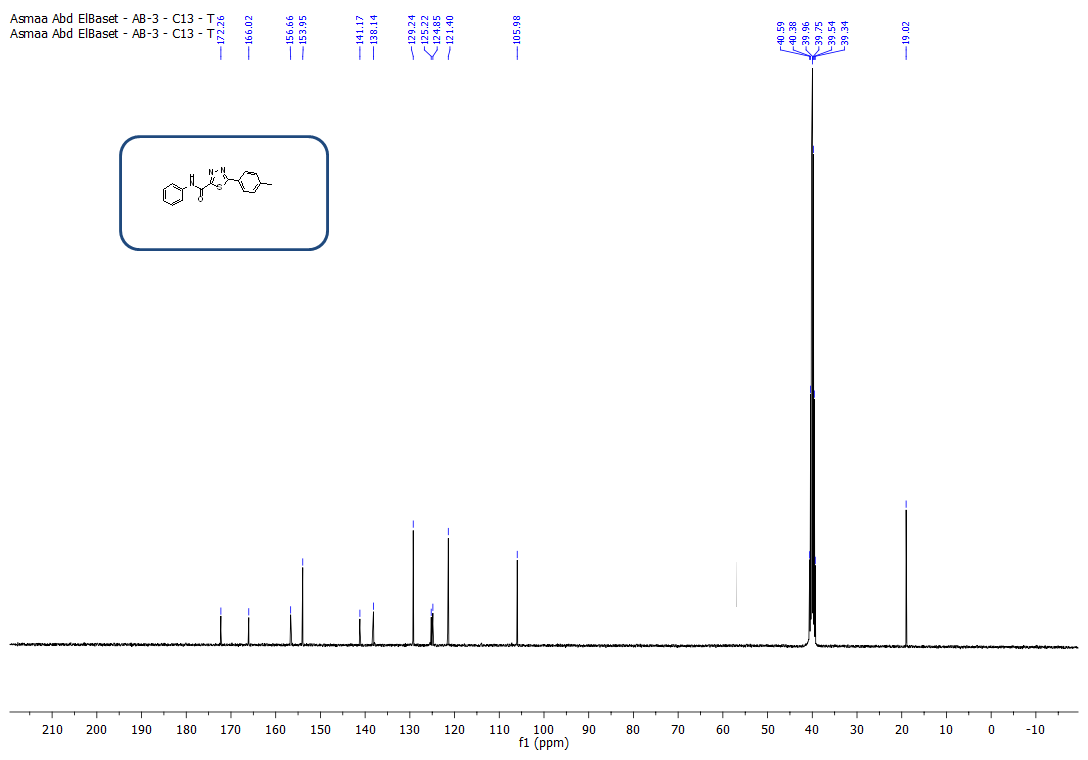


Figure S 67

***N*^5^,*N*^5'^-diphenyl-2,2',3,3'-tetrahydro[2,2'-bi(1,3,4-thiadiazole)]-5,5'-dicarboxamide** **(18a)**:

Orange crystals, yield 81%, mp. 187-189 ºC FT-IR (ATR) *λ* max: 3321, 3255 (2NH str.), 3079 (CH_arom_ str.), 2968 (CH_thiadiazole_ str.), 1693 (C=O str.), 1625 (C=N str.); ^1^H NMR: *δ* 10.19 (s, H, NH_amidic_, exchangeable by D_2_O), 9.04 (s, H, NH_thiadiazole_, exchangeable by D_2_O), 7.74- 7.06 (m, 5H, ArH), 5.53 (s, 2H, CH_thiadiazole_) ppm; ^13^C NMR: *δ* 159.13 (C=O), 139.04 (C, Thiadiazole), 138.66, 129.05, 124.29, 120.74 (Arom.), 76.13 ppm (CH_thiadiazole_); Dept-135 NMR; 129.09, 129.25, 124.31, 120.74 (Arom.), 75.98 ppm (CH_thiadiazole_) Anal. Calcd. for C_18_H_16_N_6_O_2_S_2_ (412.49): C, 52.41; H, 3.91; N, 20.37; S, 15.55% Found: C, 52.46; H, 3.90; N, 20.39; S, 15.52%.


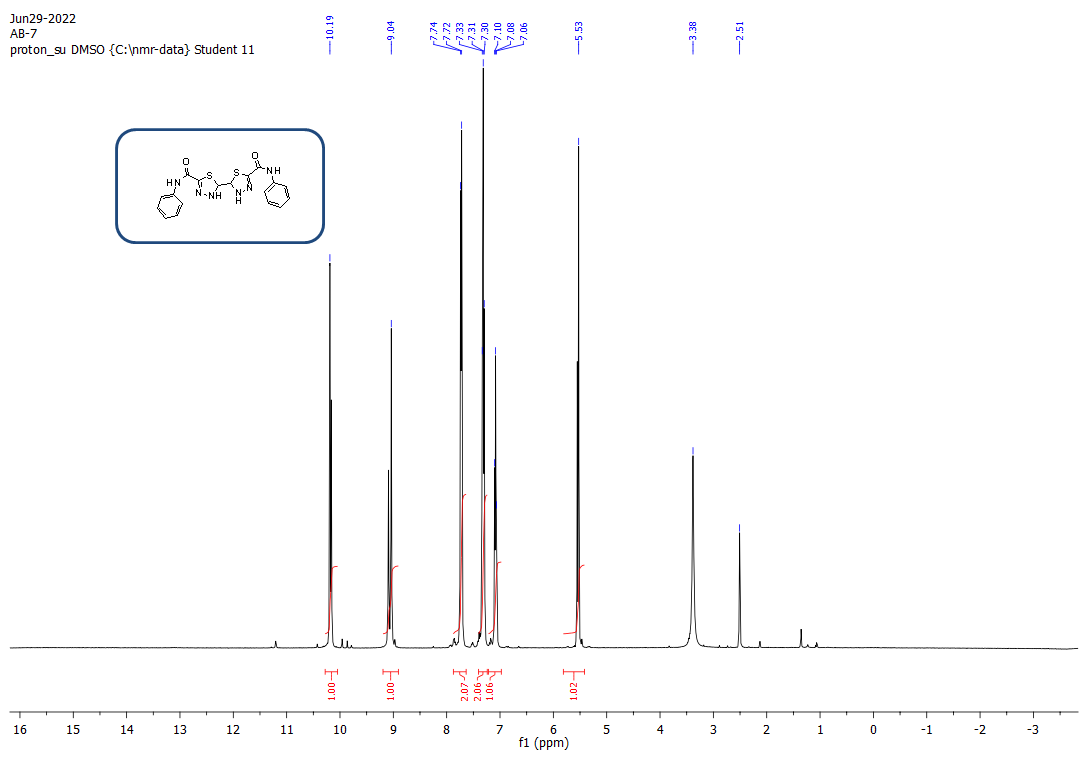


Figure S 68


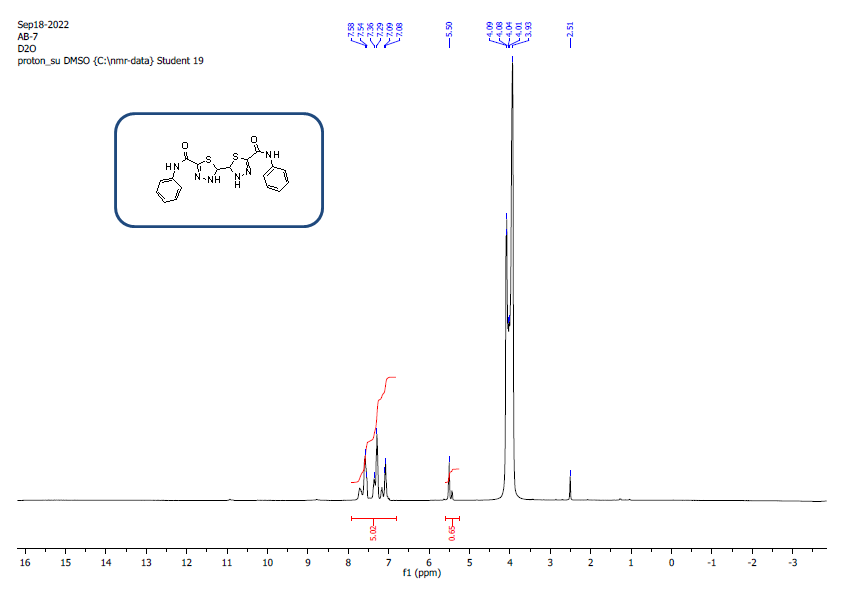


Figure S 69


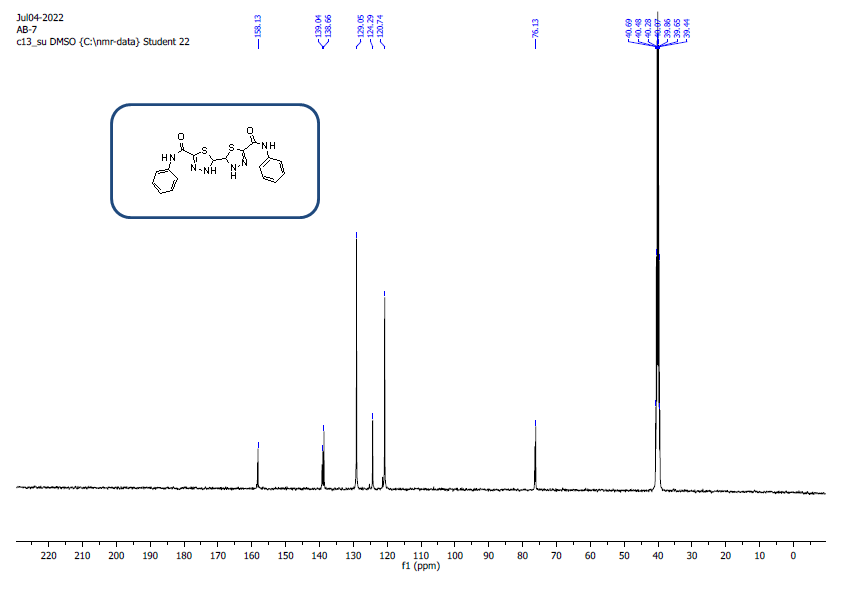


Figure S 70


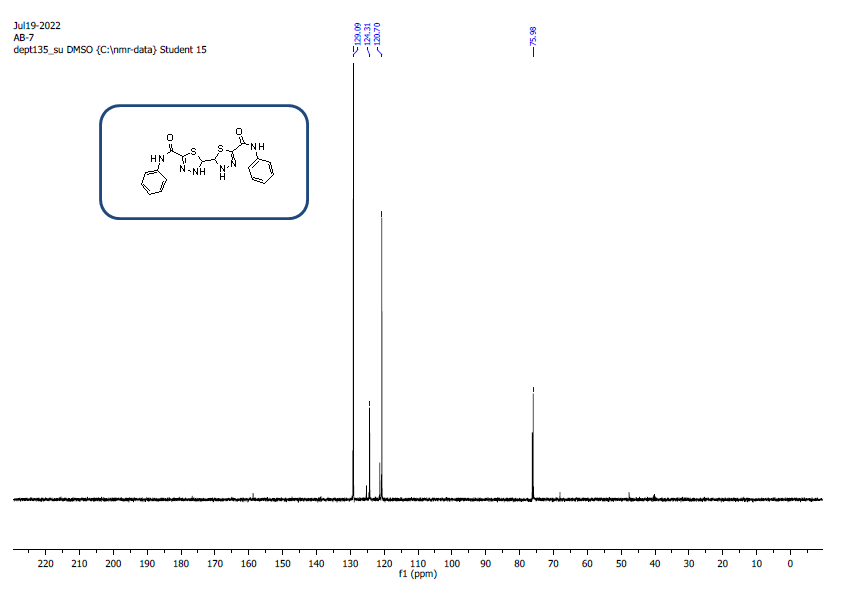


Figure S 71
